# Aggresome assembly at the centrosome is driven by CP110–CEP97–CEP290 and centriolar satellites

**DOI:** 10.1038/s41556-022-00869-0

**Published:** 2022-04-11

**Authors:** Suzanna L. Prosser, Johnny Tkach, Ladan Gheiratmand, Jaeyoun Kim, Brian Raught, Ciaran G. Morrison, Laurence Pelletier

**Affiliations:** 1 Lunenfeld-Tanenbaum Research Institute, Sinai Health System, Toronto, Ontario Canada; 2 Princess Margaret Cancer Centre, University Health Network, Toronto, Ontario Canada; 3 Department of Medical Biophysics, University of Toronto, Toronto, Ontario Canada; 4 Centre for Chromosome Biology, School of Natural Sciences, National University of Ireland Galway, Galway, Ireland; 5 Department of Molecular Genetics, University of Toronto, Toronto, Ontario Canada

**Keywords:** Centrosome, Protein aggregation, Proteasome, Autophagy

## Abstract

Protein degradation is critical to maintaining cellular homeostasis, and perturbation of the ubiquitin proteasome system leads to the accumulation of protein aggregates. These aggregates are either directed towards autophagy for destruction or sequestered into an inclusion, termed the aggresome, at the centrosome. Utilizing high-resolution quantitative analysis, here, we define aggresome assembly at the centrosome in human cells. Centriolar satellites are proteinaceous granules implicated in the trafficking of proteins to the centrosome. During aggresome assembly, satellites were required for the growth of the aggresomal structure from an initial ring of phosphorylated HSP27 deposited around the centrioles. The seeding of this phosphorylated HSP27 ring depended on the centrosomal proteins CP110, CEP97 and CEP290. Owing to limiting amounts of CP110, senescent cells, which are characterized by the accumulation of protein aggregates, were defective in aggresome formation. Furthermore, satellites and CP110–CEP97–CEP290 were required for the aggregation of mutant huntingtin. Together, these data reveal roles for CP110–CEP97–CEP290 and satellites in the control of cellular proteostasis and the aggregation of disease-relevant proteins.

## Abstract (web summary)

Prosser et al. report that centriolar satellite and centrosomal proteins seed aggresomes, perinuclear inclusions of misfolded proteins, and may play a role in aggresome formation during senescence and huntingtin aggregation.

## Main

The centrosome consists of a pair of centrioles surrounded by pericentriolar material (PCM) and serves as the main microtubule organizing centre (MTOC) of the cell^1^. It also provides a platform for localized protein degradation via the ubiquitin proteasome system (UPS). However, the contribution of centrosomal components to proteostasis remains unclear.

If the capacity of the UPS is exceeded, proteins accumulate into an inclusion termed the aggresome^2^. Aggresomes are membrane-free, juxta-nuclear, cytoplasmic inclusions that concentrate misfolded proteins, ubiquitin, molecular chaperones and proteasomes at the centrosome and the MTOC^3^. The organization of potentially toxic proteins into a single location is protective while also facilitating their clearance by autophagy^4–6^. Aggresome assembly is an active process that requires microtubules, HDAC6 and dynein^7–9^. Some centrosomal proteins can accumulate into aggresomes^2,10–12^ along with PCM1, which is a key component of centriolar satellites^13^. Satellites are proteinaceous granules associated with the centrosome that contribute to its assembly and function. Satellites also require microtubules and dynein for their association with centrosomes^14,15^. Growing evidence implicates satellites as mediators of protein stability via autophagy and the UPS^16–19^, and changes in the cellular proteome of satellite-deficient cells highlight satellites as regulators of global proteostasis^20^. However, the role of satellites in aggresome formation has yet to be explored.

Centrosome biology itself is tightly regulated by proteasomal degradation, and stringent regulation of centriole numbers is achieved through control of the abundance of centriole duplication factors^21–24^. Prolonged proteasome inhibition leads to centriole amplification^25^ and the appearance of elongated centrioles^26^. A number of proteins regulate centriole length, including CP110, which together with CEP97 forms a cap at the centriole distal end that restricts centriole elongation^27–29^. CP110 levels are regulated via the antagonistic actions of the SCF^CyclinF^ ubiquitin ligase complex^30^ and the deubiquitinating enzyme USP33 (ref. ^31^). CP110 also interacts with the satellite protein CEP290 (ref. ^32^), and both CP110 and CEP97 are found in the satellite proteome and interactome^33,34^. Whether CP110 function extends beyond the control of centriole elongation is unknown.

Senescent cells cease dividing and undergo distinctive phenotypic changes^35^. Proteasomal activity decreases during senescence, which can result in the accumulation of protein aggregates that are associated with late-onset pathologies^36–39^. Neurodegenerative disorders such as Huntington’s disease (HD) are characterized by inclusions that contain misfolded proteins, with expanded polyglutamine (polyQ) tracts in the HD protein huntingtin (HTT) associated with its aggregation^40^. HTT interacts with PCM1 through HAP1, and expression of HTT-polyQ causes PCM1 to aggregate at the centrosome^41^. However, the role of satellites in HTT-polyQ inclusion formation remains to be determined.

Here, we describe the association of centriolar and PCM proteins with the aggresome. Centriolar satellites also accumulated into this structure, and aggresome assembly was reduced in cells depleted of the satellite proteins AZI1 (also known as CEP131), CCDC14, KIAA0753 and PIBF1 (also known as CEP90). In these cells, protein aggregates were directed towards autophagy rather than the aggresome. However, in cells devoid of satellites (via depletion of PCM1), inhibition of autophagy failed to rescue aggresome formation, which places satellites at the intersection of the aggresome and autophagy pathways. Furthermore, a module consisting of CP110, CEP97 and CEP290 operates early in the aggresome pathway, and aggresome formation was defective in senescent cells due to limiting levels of CP110. Finally, satellites and the CP110–CEP97–CEP290 module function in the formation of HTT-polyQ inclusions.

## Results

### Centrosomal proteins localize to the aggresome

**Fig. 1:**
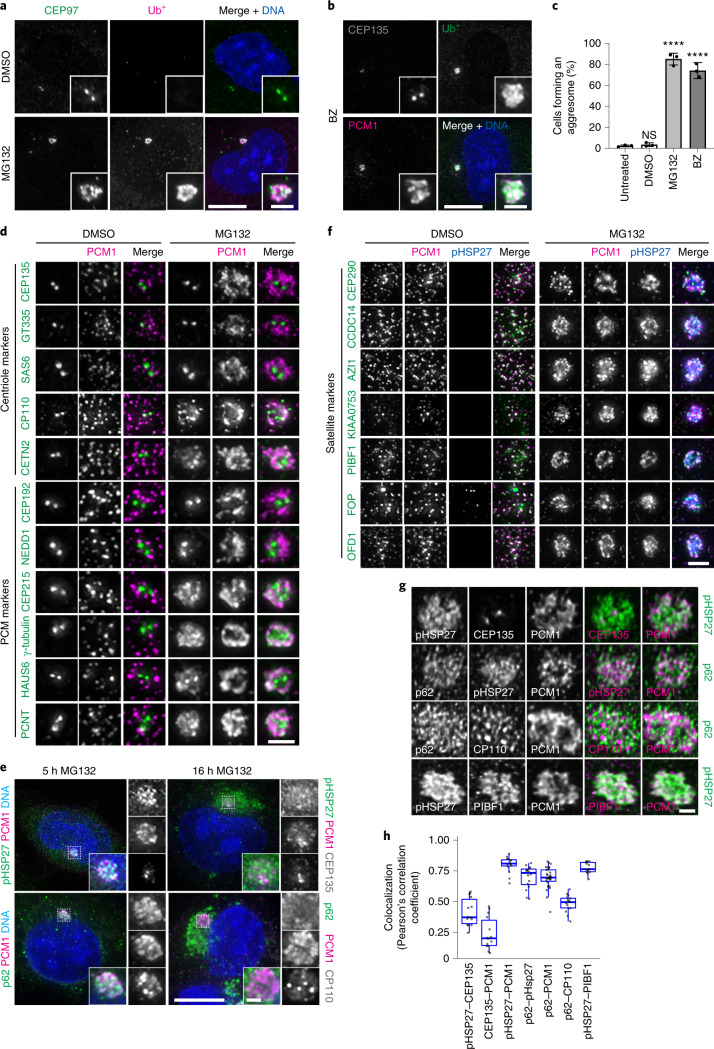
Centrosome and centriolar satellite proteins localize to the aggresome after proteasome inhibition. **a**, RPE-1 cells treated with DMSO or MG132 were stained for CEP97, ubiquitinated proteins (Ub^+^) and DNA (4,6-diamidino-2-phenylindole (DAPI)). **b**, RPE-1 cells treated with bortezomib (BZ) were stained for CEP135, Ub^+^, PCM1 and DNA (DAPI). **c**, The percentage of cells that formed an aggresome in untreated cells and in cells treated with DMSO, MG132 or BZ as revealed by Ub^+^ staining. Data displayed as the mean ± s.d., *n* = 3 independent experiments. ****P < 0.0001 or not significant (NS) by two-tailed unpaired Student’s *t*-test. **d**, RPE-1 cells treated with DMSO or MG132 were stained for PCM1 and the indicated protein. **e**, A-375 cells treated with MG132 for 5 or 16 h were stained as indicated. **f**, RPE-1 cells treated with DMSO or MG132 were stained for PCM1, pHSP27 and the indicated protein. **g**, Super-resolution images of aggresomes in MG132-treated RPE-1 cells stained as indicated. **h**, Colocalization between the indicated protein pairs from individual *z*-planes of super-resolution images of RPE-1 cells treated with MG132 displayed using Pearson’s correlation co-efficient. Boxes represent the median, upper and lower quartiles, whiskers represent 1.5× the interquartile range, with individual values from two independent experiments superimposed. Scale bars, 1 μm (**g**), 2 μm (**d**,**f**; insets of **a**,**b**,**e**) or 10 μm (**a**,**b**,**e**). Numerical data and *P* values are provided as source data. Source data

**Extended Data Fig. 1:**
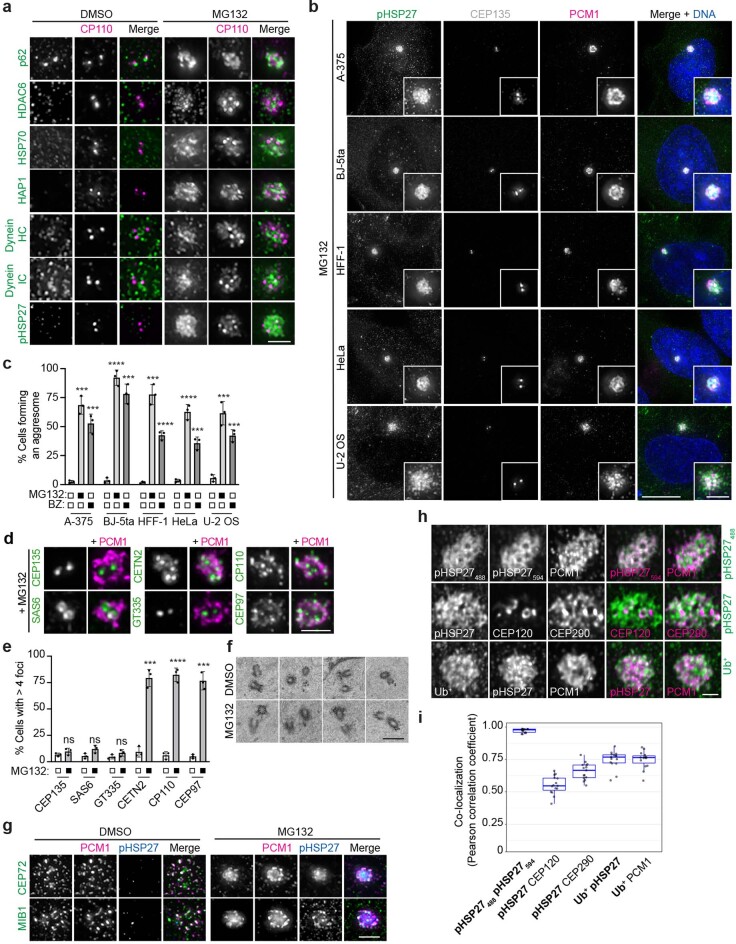
Centrosomal proteins localize to the aggresome upon proteasome inhibition. (**a)** RPE-1 cells treated with DMSO or MG132 were stained for CP110 and the indicated protein. Scale bar 2 μm. (**b**) The indicated cell lines were treated with MG132 and stained for pHSP27, CEP135 and PCM1. DNA was stained with DAPI. Scale bar 10 μm, inset 2 μm. (**c**) Quantification of the percentage cells forming an aggresome upon DMSO, MG132 and BZ treatment, as indicated. Data displayed as mean ± s.d., *n* = 3 independent experiments. ****p < 0.0001; ***p < 0.001. (**d**) RPE-1 cells treated with MG132 were stained for PCM1 and the indicated protein. Scale bar 2 μm. (**e**) Histogram showing the percentage of cells with greater than 4 foci of CEP135, SAS6, GT335, CETN2, CP110 and CEP97 following MG132 treatment. Data displayed as mean ± s.d., *n* = 3 independent experiments. ****p < 0.0001; ***p < 0.001; ns, not significant. p values: 0.218071 (CEP135), 0.058423 (SAS6), 0.160943 (GT335), 0.000219 (CETN2), 0.000055 (CP110), and 0.000150 (CEP97). (**f**) TEM images of centrioles from RPE-1 cells treated with DMSO or MG132. Scale bar 500 nm. (**g**) RPE-1 cells treated with DMSO or MG132 for 5 hours were stained for PCM1, pHSP27 and the indicated protein. Scale bar 2 μm. (**h**) Super resolution images of aggresomes in MG132 treated RPE-1 cells stained as indicated. Scale bar 1 μm. (**i**) Co-localization between the indicated protein pairs from individual z-planes of super resolution images of RPE-1 cells treated with MG132 displayed using Pearson correlation co-efficient. Boxes represent the median, upper and lower quartiles, whiskers represent 1.5x the interquartile range, with individual values from two independent experiments superimposed. For c,e: p determined by two-tailed unpaired Student’s t-test. Numerical data and p values are provided as source data. Source data

Treatment of RPE-1 cells with proteasome inhibitors (MG132 and bortezomib) led to the collection of ubiquitinated proteins (Ub^+^) around the centrosome (Fig. 1a,b). The accumulation of aggresome markers, including phosphorylated HSP27 (pHSP27)^42^, HDAC6 (ref. ^9^), p62 (ref. ^43^), HSP70 (ref. ^44^), HAP1 (ref. ^45^) and dynein^8^, confirmed aggresome assembly (Extended Data Fig. 1a). More than 75% of cells formed an aggresome (Fig. 1c), which was universal to additional cell lines tested (Extended Data Fig. 1b,c).

Given that CEP97 and PCM1 are recruited to the aggresome (Fig. 1a,b), and that γ-tubulin, PCNT and ninein accumulate during proteasome inhibition^13^, we surveyed the localization of centrosomal proteins following MG132 treatment (Fig. 1d). The centriole markers CP110 and CETN2 associated with the aggresome; however, CEP135, glutamylated tubulin (GT335) and SAS6 remained restricted to the centrioles. The PCM markers CEP215, γ-tubulin, HAUS6 and PCNT localized to the aggresome, whereas CEP192 and NEDD1 stayed limited to the PCM.

Proteolysis regulates centriole number^46^, and centriole amplification occurs in U-2 OS cells following prolonged proteasome inhibition^25^. Centriolar staining typically reveals two or four foci that correspond to two or four centrioles depending on the cell cycle phase. CEP135, SAS6 and GT335 staining revealed normal centriole complement during MG132 treatment (Extended Data Fig. 1d,e), which suggests that the extra foci of CETN2, CP110 and CEP97 did not correspond to supernumerary centrioles. This result was confirmed by transmission electron microscopy (TEM; *n* = 50; Fig. 5c and Extended Data Fig. 1f).

Overnight treatment with MG132 was not tolerated by RPE-1 cells; however, larger accumulations of pHSP27 and p62 occurred at the centrosome in A-375 cells (Fig. 1e). CP110 and PCM1 were limited to an area within the aggresome similar in size to the 5-h inclusions, which suggests that this structure forms first and may potentiate the further accumulation of protein aggregates.

In addition to PCM1, other satellite proteins localized to the aggresome (Fig. 1f and Extended Data Fig. 1g). Super-resolution imaging and colocalization analysis revealed that protein aggregates (pHSP27, Ub^+^ and p62), satellite proteins (PCM1 and PIBF1) and CP110 occupy overlapping domains within the aggresome (Fig. 1g,h and Extended Data Fig. 1h,i). However, the centrioles themselves (CEP135) were mostly devoid of protein aggregates.

### Satellites accumulate in response to proteasome inhibition

**Extended Data Fig. 2:**
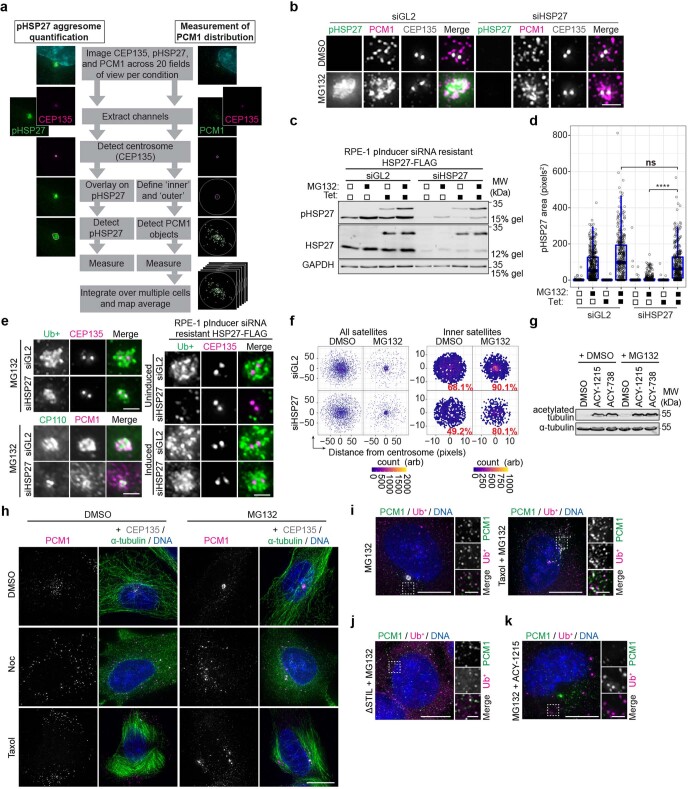
High-resolution quantitative analysis confirms a requirement for protein translation, HDAC6 and microtubules in aggresome formation. (**a**) Schematic representation of the automated aggresome quantification and satellite mapping pipeline utilized in this study (see Methods). (**b**) RPE-1 cells were transfected with iRNAs and treated and stained as indicated. Scale bar 2 μm. (**c**) Immunoblots of extracts from MG132 treated RPE-1 cells expressing an inducible siRNA resistant HSP27-FLAG construct following treatment with control (GL2) or HSP27 siRNAs. Blots were probed as indicated. (**d**) Box-and-whisker plot showing the area occupied by pHSP27 in RPE-1 pInducer siRNA resistant HSP27-FLAG cells treated as indicated. Boxes represent the median, upper and lower quartiles, whiskers represent 1.5x the interquartile range, with individual values superimposed. *n* = siGL2: 482 (un-induced DMSO), 449 (un-induced MG132), 358 (induced DMSO), 386 (induced MG132); siHSP27 437 (un-induced DMSO), 360 (un-induced MG132), 399 (induced DMSO) and 333 (induced MG132) aggresomes examined over 2 independent experiments. Data were compared using a Kruskal-Wallis ANOVA test and a post-hoc Dunn multiple comparison test performed to calculate p-values. ****p < 0.0001. (**e**) Cells transfected with siRNAs were treated and stained as indicated. Scale bar 2 μm. (**f**) Intensity maps of PCM1 distribution relative to the centrosome in cells treated as indicated. The percentage PCM1 signal residing in the ‘inner’ region is indicated. Arb, arbitrary units. (**g**) Immunoblot of extracts from cells treated and probed as indicated. (**h**) RPE-1 cells were treated and stained as indicated. Scale bar 10 μm, inset 2 μm. (**i**) RPE-1 cells treated with MG132 and taxol were stained as indicated. Scale bar 10 μm, inset 2 μm. (**j**) STIL KO cells were treated with MG132 then stained for PCM1 and Ub^+^. Scale bar 10 μm, inset 2 μm. (**k**) RPE-1 cells treated with MG132 and the HDAC6 inhibitor ACY-1215 were stained as indicated. Scale bar 10 μm, inset 2 μm. Unprocessed immunoblots, numerical data and p values are provided as source data. Source data

To examine the aggresome pathway, we established image analysis pipelines to measure aggresome size (pHSP27) and satellite distribution (PCM1; Extended Data Fig. 2a and Methods). Knockdown of HSP27 demonstrated the specificity of the pHSP27 signal (Extended Data Fig. 2b–d) and blocked aggresome assembly, as seen by Ub^+^ and CP110 staining, which was rescued following the expression of short interfering RNA (siRNA)-resistant HSP27 (Extended Data Fig. 2b–e). Notably, satellites accumulated in the pericentrosomal region during proteasome inhibition, even when HSP27 was depleted (Extended Data Fig. 2f).

**Fig. 2:**
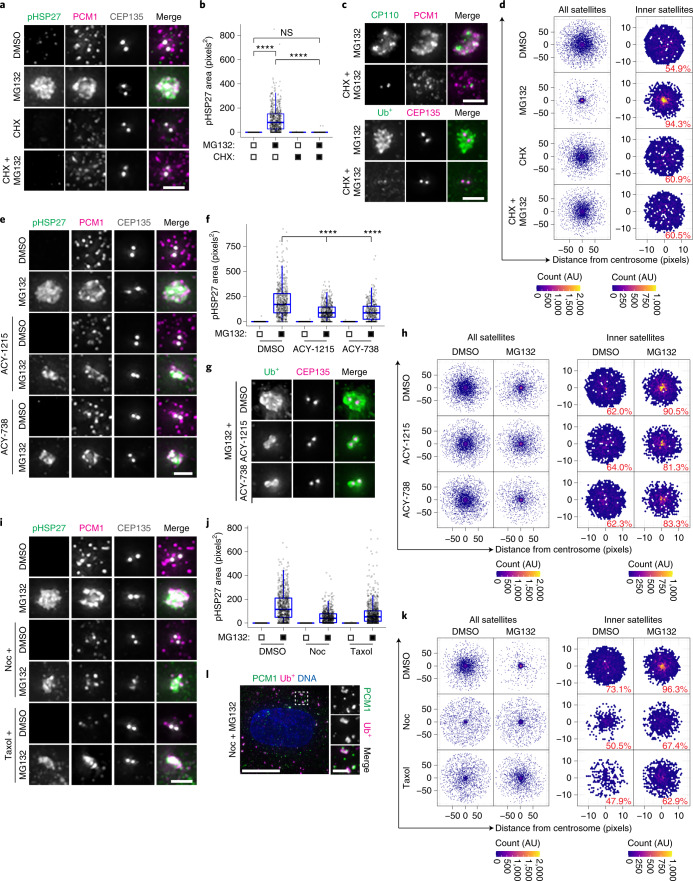
High-resolution quantitative analysis confirms the requirement for protein translation, HDAC6 and microtubules in aggresome formation. **a**, RPE-1 cells treated with DMSO, MG132, cycloheximide (CHX), or CHX plus MG132 were stained as indicated. **b**, The area occupied by pHSP27 in cells treated as in **a**. *n* = 493 (DMSO), 630 (MG132), 459 (CHX) and 437 (CHX + MG132) aggresomes examined over 2 independent experiments. ****P < 0.0001. **c**, Cells treated with MG132 with or without pretreatment with CHX were stained as indicated. **d**, Intensity maps of PCM1 distribution relative to the centrosome in cells treated as in **a**. The percentage of PCM1 signal residing in the defined inner region is indicated. AU, arbitrary units. **e**, RPE-1 cells treated with DMSO or MG132 were treated concurrently with ACY-1215 or ACY-738 and stained as indicated. **f**, The area occupied by pHSP27 in cells treated as in **e**. *n* = 586 (DMSO), 566 (MG132), 470 (ACY-1215), 504 (ACY-1215 + MG132), 470 (ACY-738) and 395 (ACY-738 + MG132) aggresomes examined over 2 independent experiments. ****P < 0.0001. **g**, RPE-1 cells treated with MG132 with or without ACY-1215 or ACY-738 were stained as indicated. **h**, PCM1 distribution relative to the centrosome in cells treated as in **e**. **i**, RPE-1 cells were pretreated with nocodazole (Noc) or taxol followed by DMSO or MG132 and stained as indicated. **j**, The area occupied by pHSP27 in cells treated as in **i**. *n* = 554 (DMSO), 499 (MG132), 413 (Noc), 422 (Noc + MG132), 510 (taxol) and 485 (taxol + MG132) aggresomes examined over 2 independent experiments. ****P < 0.0001. **k**, PCM1 distribution relative to the centrosome in cells treated as in **i**. **l**, RPE-1 cells treated with MG132 and nocodazole were stained as indicated. For **b**, **f** and **j**, boxes represent the median, upper and lower quartiles, whiskers represent 1.5× the interquartile range, with individual values superimposed. Data were compared using Kruskal–Wallis analysis of variance (ANOVA) test, and post-hoc Dunn multiple comparison test was performed to calculate *P* values. Scale bars, 2 μm (**a**,**c**,**e**,**g**,**i**; inset of **l**) or 10 μm (**l**). Numerical data and *P* values are provided as source data. AU, arbitrary units. Source data

Next, we examined approaches reported to disrupt aggresome formation. Active protein translation is required for aggresome assembly^47,48^, and pretreatment with cycloheximide to inhibit translation before MG132 addition completely blocked pHSP27 and Ub^+^ recruitment to the aggresome and satellite accumulation (Fig. 2a–d). Aggresome formation also requires HDAC6 activity^9^, so we analysed the effects of the HDAC6 inhibitors ACY-1215 and ACY-738 (ref. ^49^). Increased levels of acetylated tubulin confirmed HDAC6 inhibition (Extended Data Fig. 2g), and we observed a moderate, but significant, reduction in aggresome size rather than a complete block to aggresome assembly as reported for HDAC6 depletion^9^, with pHSP27 and Ub^+^ restricted to the area immediately around the centrioles (Fig. 2e–g). The accumulation of satellites was largely unaffected (Fig. 2h).

Finally, as aggresome formation depends on the instability of microtubule dynamics^50^, we used nocodazole and taxol to disrupt microtubules. These compounds led to satellite dispersion throughout the cytoplasm^15,51^ (Extended Data Fig. 2h). Treatment of cells with MG132 led to reduced accumulation of pHSP27 and restricted recruitment of satellites (Fig. 2i–k). Ub^+^ in these cells formed multiple cytoplasmic aggregates that colocalized with PCM1 (Fig. 2l and Extended Data Fig. 2i), which places satellites with these proteins before their recruitment to the aggresome. In cells that lacked centrioles through disruption of STIL, Ub^+^ similarly colocalized with cytoplasmic PCM1 foci (Extended Data Fig. 2j). However, Ub^+^ aggregates in ACY-1215-treated cells did not contain PCM1 (Extended Data Fig. 2k), which suggests that this association requires HDAC6.

Together, these results demonstrate the robustness of our analysis for quantitative morphometric measurements of aggresomes and for distinguishing between complete and partial blocks to aggresome assembly. Furthermore, satellites accumulate around the centrosome in response to proteasomal inhibition, downstream of the requirement for active protein translation in aggresome biogenesis.

### Centriolar satellites are required for aggresome formation

**Fig. 3:**
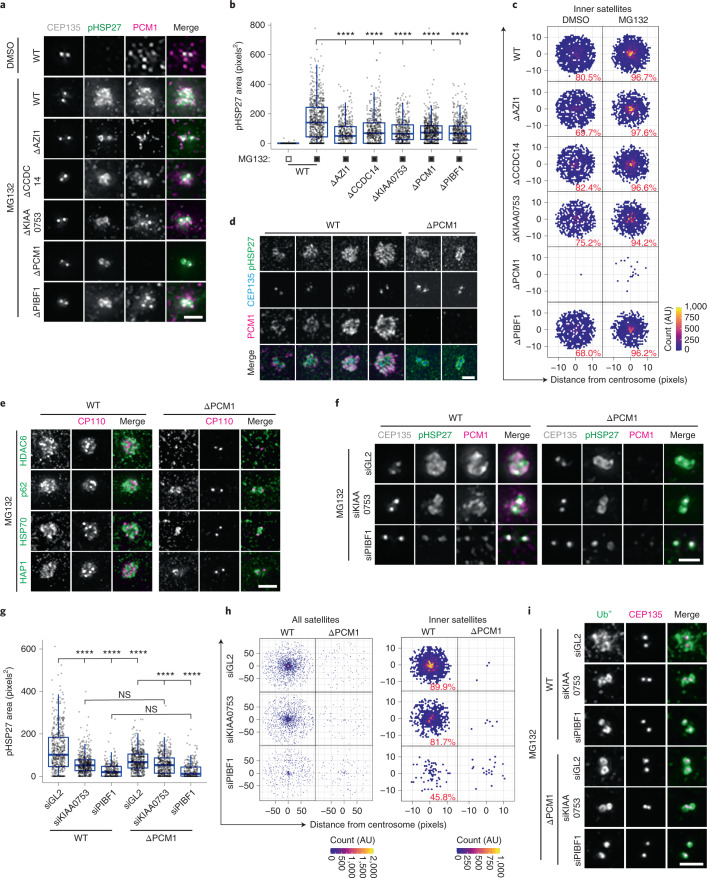
Centriolar satellites are required for aggresome formation. **a**, WT cells and ΔAZI1, ΔCCDC14, ΔKIAA0753, ΔPCM1 and ΔPIBF1 RPE-1 cells were treated with MG132 and stained as indicated. **b**, The area occupied by pHSP27 in cells treated as in **a**. *n* = 518 (WT-DMSO), 705 (WT-MG132), 498 (ΔAZI1), 560 (ΔCCDC14), 468 (ΔKIAA0753), 682 (ΔPCM1) and 550 (ΔPIBF1) aggresomes examined over 2 independent experiments. ****P < 0.0001. **c**, Intensity maps of PCM1 distribution relative to the centrosome in cells treated as treated in **a**. The percentage PCM1 signal residing in the defined inner region is indicated. **d**, Super-resolution images of pHSP27, CEP135 and PCM1 staining in WT and ΔPCM1 cells treated with MG132. **e**, WT and ΔPCM1 cells treated with MG132 were stained for CP110 and the indicated protein. **f**, WT and ΔPCM1 cells were treated with control (GL2), KIAA0753 or PIBF1 siRNAs (siGL2, siKIAA0753 and siPIBF1, respectively) as indicated for 48 h, then treated with MG132. Cells were stained for CEP135, pHSP27 and PCM1. **g**, The area occupied by pHSP27 in cells treated as in **f**. *n* = 511 (WT-siGL2), 493 (WT-siKIAA0753), 324 (WT-siPIBF1), 519 (ΔPCM1-siGL2), 452 (ΔPCM1-siKIAA0753) and 291 (ΔPCM1-siPIBF1) aggresomes examined over 2 independent experiments. ****P < 0.0001. **h**, Intensity maps of PCM1 distribution relative to the centrosome in cells treated as in **f**. The percentage PCM1 signal residing in the defined ‘inner’ region is indicated. **i**, Ub^+^ and CEP135 staining in WT and PCM1 KO cells treated as in **f**. For **b** and **g**, boxes represent the median, upper and lower quartiles, whiskers represent 1.5× the interquartile range, with individual values superimposed. Data were compared using Kruskal–Wallis ANOVA test, and post-hoc Dunn multiple comparison test was performed to calculate *P* values. Scale bars, 1 μm (**d**) or 2 μm (**a**,**e**,**f**,**i**). Numerical data and *P* values are provided as source data. AU, arbitrary units. Source data

**Extended Data Fig. 3:**
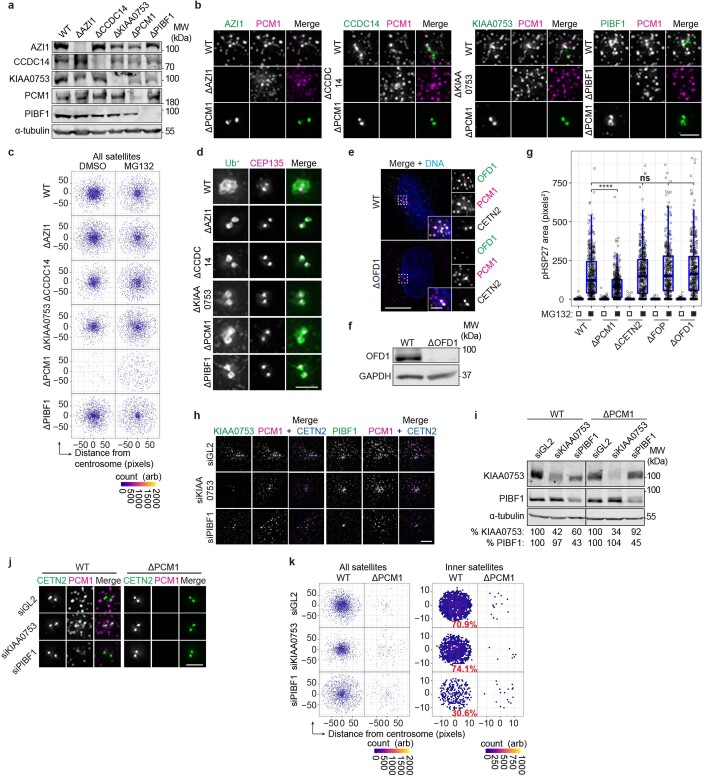
Centriolar satellites are required for aggresome formation. (**a**) CRISPR/Cas9 mediated gene disruption was used to generate AZI1, CCDC14, KIAA0753, PCM1 and PIBF1 knockout (KO) RPE-1 cell lines. Extracts from WT and KO cells were probed as indicated. (**b**) Loss of protein in AZI1, CCDC14, KIAA0753, PCM1 and PIBF1 KO cells was also confirmed by IF microscopy. Scale bar 2 μm. (**c**) Intensity maps of PCM1 distribution relative to the centrosome in WT and KO cells treated as indicated for 5 hours. Arb, arbitrary units. (**d**) Ub^+^ and CEP135 staining in WT and satellite protein KO cells treated with MG132. Scale bar 2 μm. (**e**) WT and OFD1 KO cells were stained for OFD1, PCM1 and CETN2. Scale bar 10 μm, inset 2 μm. (**f**) Immunoblot of extracts from WT and OFD1 KO cells probed as indicated. (**g**) Box-and-whisker plot showing the area occupied by pHSP27 in the indicated cell lines treated with MG132. Boxes represent the median, upper and lower quartiles, whiskers represent 1.5x the interquartile range, with individual values superimposed. *n* = WT: 400 (DMSO), 414 (MG132); ΔCETN2: 411 (DMSO), 434 (MG132); ΔFOP: 456 (DMSO), 352 (MG132); ΔOFD1: 422 (DMSO), 377 (MG132); ΔPCM1: 519 (DMSO) and 429 (MG132) aggresomes examined over 2 independent experiments. Data were compared using a Kruskal-Wallis ANOVA test and a post-hoc Dunn multiple comparison test performed to calculate p-values. ****p < 0.0001; n.s. not significant. (**h**) WT cells were transfected with siRNAs and stained as indicated. Scale bar 2 μm. (**i**) Immunoblot of extracts from WT and PCM1 KO cell lines transfected with siRNAs. All samples were run on the same gels; however, an intervening lane between the WT and PCM1 KO cell lines was excised, thereby placing non-adjacent lanes next to each other in the panel. Numbers at the bottom indicate the mean band intensity expressed as a percentage of signal of the corresponding control for the indicated proteins. (**j**) WT and PCM1 KO cells treated with control, KIAA0753 or PIBF1 siRNAs were stained as indicated. Scale bar 2 μm. (**k**) Intensity maps of PCM1 distribution relative to the centrosome in WT and PCM1 KO cells treated and stained as in **j**. Arb, arbitrary units. Unprocessed immunoblots, numerical data and p values are provided as source data. Source data

To test the requirement for satellites in aggresome assembly, we used CRISPR–Cas9 to disrupt *AZI1*, *CCDC14*, *KIAA0753* and *PIBF1* expression. Sequence analysis confirmed gene disruption (Supplementary Table 1), and immunoblotting and immunofluorescence (IF) microscopy confirmed protein loss (Extended Data Fig. 3a,b). In each knockout (KO) line, PCM1-positive satellites persisted, whereas AZI1, CCDC14, KIAA0753 and PIBF1 were restricted to the centrioles in a previously generated PCM1 KO line^33^ (Extended Data Fig. 3b). This result indicated satellite loss and is in line with previous studies^17,20,52^. Aggresome assembly was reduced to similar extents in each KO line despite PCM1-positive satellites accumulating in the pericentrosomal region of AZI1, CCDC14, KIAA0753 and PIBF1 KO cells (Fig. 3a–c and Extended Data Fig. 3c). Conversely, aggresome formation was not impeded in cells depleted of CETN2 (ref. ^53^), FOP^54^ or OFD1 (this study; Extended Data Fig. 3e–g and Supplementary Table 1), despite these proteins accumulating within the aggresome (Fig. 1d,f).

pHSP27 was restricted to a ring around each centriole in PCM1 KO cells, which corresponded to an early stage of aggresome biogenesis compared with wild-type (WT) cells at different points in aggresome assembly (Fig. 3d). Ub^+^ was similarly restricted (Extended Data Fig. 3d), and other aggresome markers failed to accumulate (Fig. 3e).

Despite lacking satellites, PCM1 KO cells retained a centrosomal pool of AZI1, CCDC14, KIAA0753 and PIBF1 (Extended Data Fig. 3b). As each KO cell line had similarly impaired aggresome formation to the PCM1 KO cell line, this suggests that the satellite, rather than centrosomal, pool of these proteins is involved in aggresome assembly. To test this, we depleted KIAA0753 and PIBF1 by siRNA in WT and PCM1 KO cells. Knockdown was confirmed by IF microscopy and immunoblotting (Extended Data Fig. 3h,i). In untreated cells, satellite distribution in control cells and in KIAA0753-depleted cells was comparable, but PIBF1 depletion reduced satellite abundance (Extended Data Fig. 3j,k). In WT cells, KIAA0753 and PIBF1 knockdown reduced aggresome size, with the greatest effect observed for PIBF1 depletion (Fig. 3f–h). The accumulation of Ub^+^ was also reduced (Fig. 3i). In PCM1 KO cells, KIAA0753 and PIBF1 knockdown further modestly reduced aggresome size, with PIBF1 again having the greatest effect (Fig. 3f,g); however, this was not significantly different to WT cells. This result suggests that the centrosomal pools of these proteins have a minor role in aggresome formation.

### Centriolar satellites link aggresome assembly and autophagy

**Extended Data Fig. 4:**
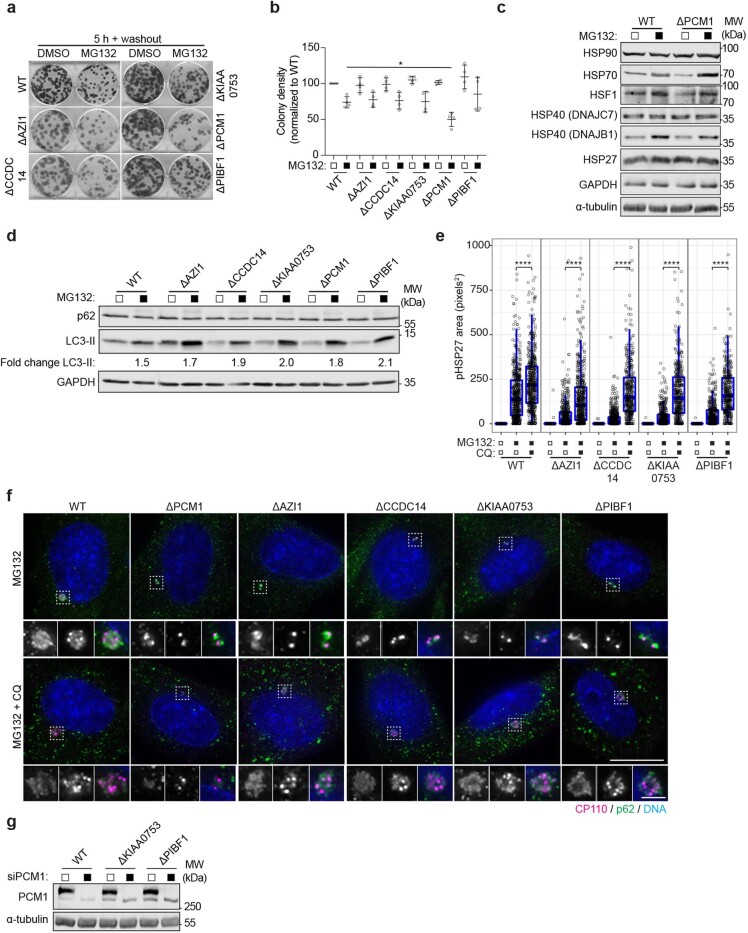
Centriolar satellites direct protein aggregates to the aggresome in the absence of autophagy. (**a**) WT, AZI1, CCDC14, KIAA0753, PCM1 and PIBF1 KO cells were seeded for clonogenic assays and treated with MG132 for 5 hours. The drug was then extensively washed out and the cells left to grow for 12 days. (**b**) Colony density was quantified and growth normalized to WT. Mean ± s.d. is shown, n = 4 from two independent experiments. *p < 0.05 by two-tailed unpaired Student’s t-test. (**c**) Immunoblots of extracts from WT and PCM1 KO cells treated with DMSO or MG132 probed as indicated. α-tubulin was used as a loading control. (**d**) Immunoblots of extracts from WT, AZI1, CCDC14, KIAA0753, PCM1 and PIBF1 KO cells treated with DMSO or MG132 and probed as indicated. The fold change in the amount of LC3-II present in MG132 treated cells as compared to the DMSO treated counterpart is indicated. GAPDH was used as a loading control. (**e**) Box-and-whisker plot showing the area occupied by pHSP27 in WT, AZI1, CCDC14, KIAA0753 and PIBF1 KO cells treated with DMSO, MG132 or MG132 plus chloroquine (CQ). Boxes represent the median, upper and lower quartiles, whiskers represent 1.5x the interquartile range, with individual values superimposed. *n* = WT: 470 (DMSO), 529 (MG132), 513 (MG132/CQ); ΔAZI1: 484 (DMSO), 458 (MG132), 524 (MG132/CQ); ΔCCDC14: 387 (DMSO), 739 (MG132), 404 (MG132/CQ); ΔKIAA0753: 381 (DMSO), 426 (MG132), 411 (MG132/CQ); ΔPIBF1: 470 (DMSO), 529 (MG132) and 513 (MG132/CQ) aggresomes examined over 2 independent experiments. Data were compared using a Kruskal-Wallis ANOVA test and a post-hoc Dunn multiple comparison test performed to calculate p-values. ****p < 0.0001. (**f**) WT, PCM1, AZI1, CCDC14, KIAA0753 and PIBF1 cells were treated with MG132 or MG132 plus CQ and stained for CP110 and p62. Scale bar 10 μm, 2 μm. (**g**) Immunoblot of extracts from WT, KIAA0753 and PIBF1 KO cells transfected with siRNAs against PCM1. α-tubulin was used as a loading control. Unprocessed immunoblots, numerical data and p values are provided as source data. Source data

Given that they have different effects on satellites, it was surprising that AZI1, CCDC14, KIAA0753 and PIBF1 KO affected aggresome assembly to the same degree as PCM1 KO. To examine the consequence of impeded aggresome formation in these cell lines, we performed clonogenic survival assays following treatment with MG132. PCM1 KO cells displayed decreased fitness compared with the other KO cell lines (Extended Data Fig. 4a,b). However, there was no evidence of increased baseline proteotoxic stress in the PCM1 KO cells (Extended Data Fig. 4c).

**Fig. 4:**
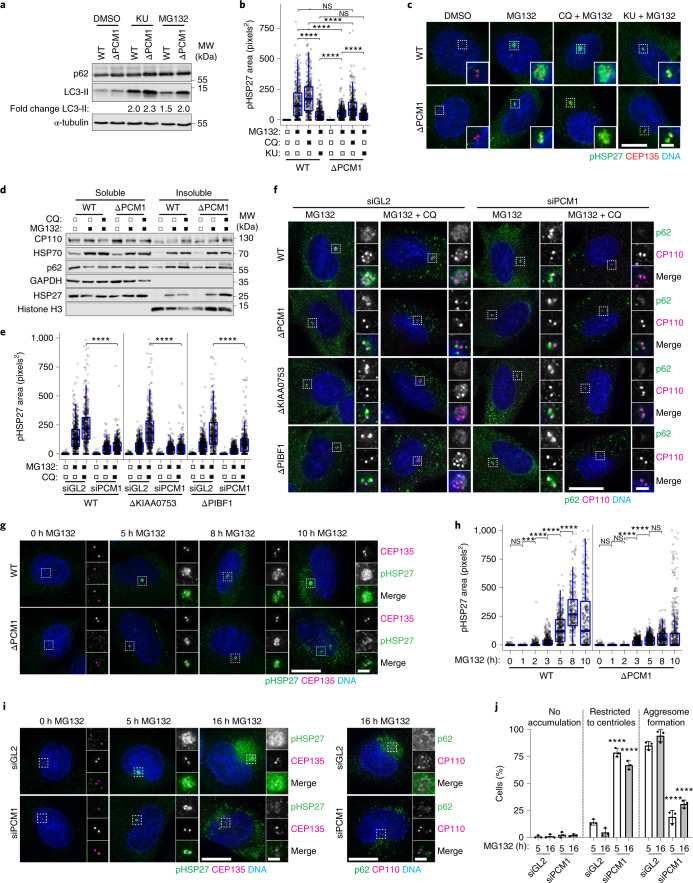
Centriolar satellites direct protein aggregates to the aggresome in the absence of autophagy. **a**, WT and ΔPCM1 cells treated and probed as indicated. KU, KU-0063794. **b**, pHSP27 area in WT and ΔPCM1 cells treated as indicated. *n* = 360 (WT-DMSO), 368 (WT-MG132), 323 (WT-MG132 + CQ), 386 (WT-MG132 + KU), 360 (ΔPCM1-DMSO), 375 (ΔPCM1-MG132), 366 (ΔPCM1-MG132 + CQ) and 418 (ΔPCM1-MG132 + KU) aggresomes examined over 2 independent experiments. ****P < 0.0001. **c**, Cells treated as in **b** were stained as indicated. **d**, Fractions from WT and ΔPCM1 cells treated and probed as indicated. **e**, pHSP27 area in siRNA-transfected WT, ΔKIAA0753 and ΔPIBF1 cells treated as indicated. Number of aggresomes examined over 2 experiments: WT: *n* = 522 (siGL2-DMSO), 525 (siGL2-MG132), 433 (siGL2-MG132 + CQ), 466 (siPCM1-DMSO), 422 (siPCM1-MG132) and 460 (siPCM1-MG132 + CQ); ΔKIAA0753: *n* = 509 (siGL2-DMSO), 563 (siGL2-MG132), 503 (siGL2-MG132 + CQ), 474 (siPCM1-DMSO), 442 (siPCM1-MG132) and 380 (siPCM1-MG132 + CQ); ΔPIBF1: *n* = 680 (siGL2-DMSO), 608 (siGL2-MG132), 478 (siGL2-MG132/CQ), 497 (siPCM1-DMSO), 531 (siPCM1-MG132) and 427 (siPCM1-MG132/CQ). ****P < 0.0001. **f**, siRNA-transfected WT cells and ΔPCM1, ΔKIAA0753 and ΔPIBF1 cells were treated and stained as indicated. **g**, WT and ΔPCM1 cells were treated and stained as indicated. **h**, pHSP27 area in WT and ΔPCMI cells treated as indicated. Number of aggresomes examined over 2 independent experiments: WT: *n* = 423 (0 h), 387 (1 h), 428 (2 h), 470 (3 h), 470 (4 h), 497 (5 h), 392 (8 h) and 287 (10 h); ΔPCM1: *n* = 524 (0 h), 581 (1 h), 580 (2 h), 598 (3 h), 640 (4 h), 635 (5 h), 593 (8 h) and 394 (10 h). ****P < 0.0001, **P < 0.01. **i**, siRNA-transfected A-375 cells were treated and stained as indicated. **j**, Quantitation of aggresome formation in A-375 cells transfected with control or PCM1 siRNAs, then treated with MG132 as indicated. Data displayed as the mean ± s.d., *n* = 3 independent experiments. ****P < 0.0001 by two-tailed unpaired Student’s *t*-test. For **b**, **e** and **h**, boxes represent the median, upper and lower quartiles, whiskers represent 1.5× the interquartile range, with individual values superimposed. Data compared using Kruskal–Wallis ANOVA test and post-hoc Dunn multiple comparison test. Scale bars, 10 μm (**c**,**f**,**g**,**i**) or 2 μm (insets of **c**,**f**,**g**,**i**). Unprocessed immunoblots, numerical data and *P* values are provided as source data. Source data

The UPS and autophagy are interconnected, with disruption of one causing upregulation of the other^55,56^. Aggresome size is a function of the production, transport and clearance of protein aggregates, and satellites can regulate autophagy^16^; therefore we examined autophagy in PCM1 KO cells. Detection of LC3-II by immunoblotting showed that there was a stronger induction of autophagy in the PCM1 KO cells than in WT cells (Fig. 4a). Notably, activation of autophagy in WT cells reduced aggresome size to below levels observed in PCM1 KO cells treated with MG132 (Fig. 4a–c). It is probable that pre-activation of autophagy before the addition of MG132 accounts for this stronger effect. Inhibition of autophagy however, only moderately rescued aggresome size in the PCM1 KO cells, with aggresomes failing to reach the size seen in MG132-treated WT cells. The appearance of cytoplasmic pHSP27 aggregates in PCM1 KO cells suggested that aggregate production was not impeded (Fig. 4c), which was supported by the presence of HSP70, p62 and HSP27 in the insoluble fraction of PCM1 KO cells (Fig. 4d).

The other KO cell lines also displayed stronger autophagy induction than WT cells (Extended Data Fig. 4d). However, inhibition of autophagy rescued aggresome size and led to the accumulation of CP110 and p62 at the centrosome in these cells (Extended Data Fig. 4e,f). Knockdown of PCM1 demonstrated that this rescue depended on satellites (Fig. 4e,f and Extended Data Fig. 4g).

To determine whether aggresomes can grow larger in PCM1 KO cells, we conducted a time course of MG132 treatment. In WT cells, pHSP27 began to accumulate around the centrosome at 2 h and continued to increase in size until 8–10 h (Fig. 4g,h). By contrast, in PCM1 KOs, pHSP27 reached its maximum size by 3–5 h. Depletion of PCM1 similarly affected aggresome formation in A-375 cells treated with MG132 overnight (Fig. 4i,j), with pHSP27 and p62 failing to assemble into a single inclusion despite aggregates accumulating in these cells. Together, these data support the requirement for centriolar satellites in aggresome formation and that protein aggregates are directed towards autophagy in their absence.

### The CP110 module is required for aggresome formation

**Fig. 5:**
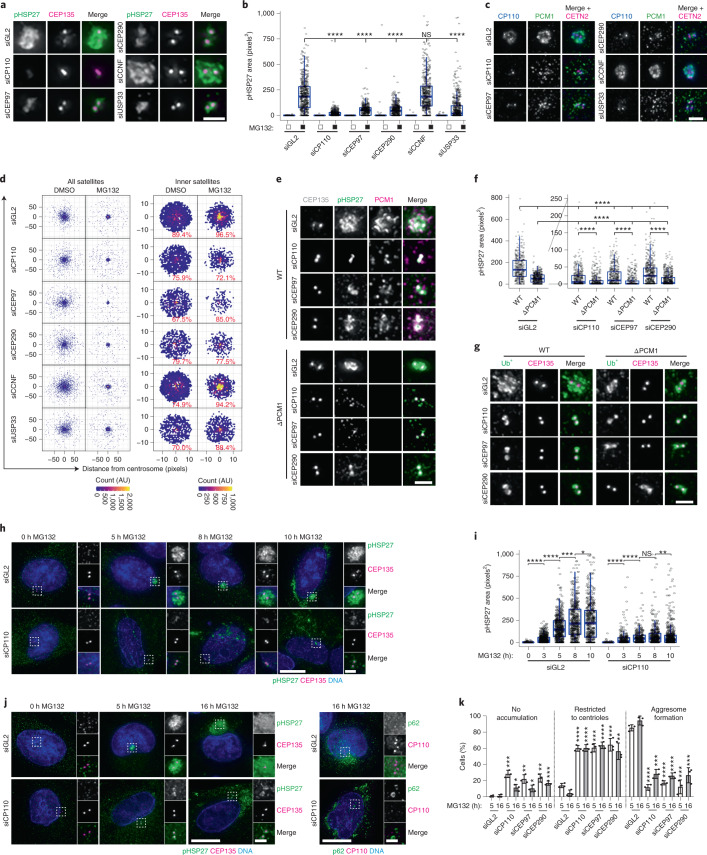
A CP110–CEP97–CEP290 module is required for aggresome formation. **a**, siRNA-transfected cells were treated and stained as indicated. **b**, Quantitation of pHSP27 area in cells treated as in **a**. *n* = DMSO: 705 (siGL2), 376 (siCP110), 372 (siCEP97), 691 (siCEP290), 579 (siCCNF), 472 (siUSP33); MG132: 517 (siGL2), 455 (siCP110), 396 (siCEP97), 449 (siCEP290), 475 (siCCNF) and 555 (siUSP33) aggresomes examined over 2 independent experiments. ****P < 0.0001. **c**, Cells treated as in **a** were stained as indicated. **d**, PCM1 distribution relative to the centrosome in cells treated as in **a**. The percentage of PCM1 in the inner region is indicated. **e**, siRNA-transfected WT and ΔPCM1 cells were treated and stained as indicated. **f**, pHSP27 area in cells treated as in **e**, with data from the same experiment displayed with different *y* axis ranges to ease comparison between knockdowns. Number of aggresomes examined over 2 independent experiments: WT: *n* = 408 (siGL2), 491 (siCP110), 518 (siCEP97) and 578 (siCEP290); ΔPCM1: *n* = 518 (siGL2), 510 (siCP110), 494 (siCEP97) and 699 (siCEP290). ****P < 0.0001. **g**, Cells treated as in **e** were stained as indicated. **h**, siRNA-transfected cells were treated and stained as indicated. **i**, pHSP27 area in siRNA-transfected WT cells treated with MG132. Number of aggresomes examined over 2 independent experiments: siGL2: *n* = 524 (0 h), 664 (3 h), 533 (5 h), 510 (8 h) and 519 (10 h); siCP110: *n* = 413 (0 h), 229 (3 h), 312 (5 h), 430 (8 h) and 550 (10 h). ****P < 0.0001, **P < 0.01, *P < 0.05. **j**, A-375 cells transfected with siGL2 or siCP110 were treated and stained as indicated. **k**, Aggresome formation in A-375 cells transfected and treated as indicated. Data displayed as the mean ± s.d., *n* = 3 independent experiments. ****P < 0.0001, **P < 0.01, *P < 0.05 by two-tailed unpaired Student’s *t*-test. For **b**, **f** and **i**, boxes represent the median, upper and lower quartiles, whiskers represent 1.5× the interquartile range, with individual values superimposed. Data were compared using Kruskal–Wallis ANOVA test and post-hoc Dunn multiple comparison test. Scale bars, 2 μm (**a**,**c**,**e**,**g**; insets of **h**,**j**) or 10 μm (**h**,**j**). Numerical data and *P* values are provided as source data. AU, arbitrary units. Source data

**Extended Data Fig. 5:**
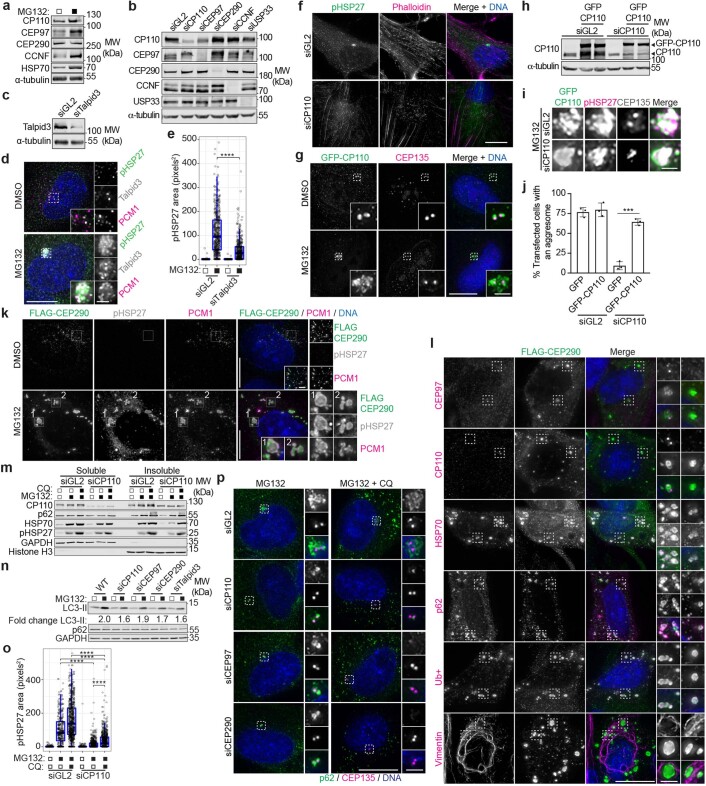
A CP110-CEP97-CEP290 module is required for aggresome formation. (**a**) MG132 treated extracts were probed as indicated. (**b**) Extracts from siRNA transfected cells were probed as indicated. (**c**) Extracts from control or Talpid3 siRNAs treated cells were probed as indicated. (**d**) Cells treated with DMSO or MG132 were stained as indicated. Scale bar 10 μm, inset 2 μm. (**e**) Plot of pHSP27 area in cells transfected with siRNAs and treated with MG132. Boxes represent the median, upper and lower quartiles, whiskers represent 1.5x the interquartile range, with individual values superimposed. *n* = siGL2: 459 (DMSO), 463 (MG132); siTalpid3: 372 (DMSO) and 388 (MG132) aggresomes examined over 2 independent experiments. Data were compared using a Kruskal-Wallis ANOVA test and a post-hoc Dunn multiple comparison test performed to calculate p-values. ****p < 0.0001. (**f**) RPE-1 cells were treated and stained as indicated. Scale bar 10 μm. (**g**) RPE-1 cells transfected with GFP-CP110 were treated and stained as indicated. Scale bar 10 μm, inset 2 μm. (**h**) Extracts from RPE-1 cells treated with CP110 siRNAs and transiently transfected with RNAi-resistant GFP-CP110 were probed as indicated. (**i**) Cells transfected as in **h** were treated with MG132 and stained as indicated. Scale bar 2 μm. (**j**) Percentage cells treated as in **i** that formed an aggresome. Data displayed as mean ± s.d., *n* = 3 independent experiments. ****p < 0.0001 by two-tailed unpaired Student’s t-test. (**k, l**) Cells transfected with FLAG-CEP290 were treated and stained as indicated. Scale bars 10 μm, inset 2 μm. (**m**) Fractions from cells transfected with siRNAs and treated as indicated (CQ, chloroquine). (**n**) Extracts from cells transfected with siRNAs were treated and probed as indicated. The fold change in LC3-II levels in MG132 treated cells is shown as compared to their control treated counterparts. (**o**) Plot showing pHSP27 area in WT cells transfected siRNAs and treated with MG132 or MG132 and CQ. Boxes represent the median, upper and lower quartiles, whiskers represent 1.5x the interquartile range, with individual values superimposed. *n* = siGL2: 766 (DMSO), 719 (MG132), 741 (MG132/CQ); siCP110: 810 (DMSO), 577 (MG132) and 659 (MG132/CQ) aggresomes examined across 2 independent experiments. Data were compared using a Kruskal-Wallis ANOVA test and a post-hoc Dunn multiple comparison test performed to calculate p-values. ****p < 0.0001. (**p**) siRNA transfected cells were treated with MG132 or MG132 plus CQ and stained as indicated. Scale bar 10 μm, inset 2 μm. Unprocessed immunoblots, numerical data and p values are provided as source data. Source data

Despite localizing to the aggresome, CETN2 was not required for aggresome formation (Extended Data Fig. 3g). To further assess the contribution of centriolar proteins to aggresome formation, we analysed CP110, CEP97 and CEP290. CP110 is stabilized by USP33 and degraded by the UPS via cyclin F (CCNF), with proteasome inhibition stabilizing both CP110 (ref. ^31^) and CEP97 levels (Extended Data Fig. 5a). Knockdown of CP110, CEP97, CEP290 and USP33 reduced aggresome assembly (Fig. 5a,b and Extended Data Fig. 5b). Depletion of the CP110 interactor TALPID3 (ref. ^57^), which also localizes to the aggresome, similarly reduced aggresome size (Extended Data Fig. 5c–e). CP110 depletion had the greatest effect, causing mis-localization of pHSP27 to actin filaments (Extended Data Fig. 5f), and RNA interference (RNAi)-resistant CP110 rescued aggresome formation in CP110-depleted cells (Extended Data Fig. 5g–j). Conversely, MG132 treatment of CEP290-transfected cells resulted in multiple inclusions that contained pHSP27, HSP70, p62, Ub^+^ and PCM1 throughout the cytoplasm (Extended Data Fig. 5k,l). Although CP110 and CEP97 did not localize to these inclusions, their accumulation at the centrosome was blocked.

Satellite accumulation was reduced in cells depleted in CP110, CEP97 and CEP290 (Fig. 5c,d). In PCM1 KO cells, depletion of CP110, CEP97 or CEP290 had an additive effect on aggresome assembly (Fig. 5e,f), which suggests that satellites are required for a fraction of the recruitment of pHSP27 to the centrioles in the absence of CP110, CEP97 or CEP290. Moreover, CP110, CEP97 and CEP290 are responsible for the accumulation of a proportion of pHSP27 in the absence of satellites. The recruitment of Ub^+^ was restricted accordingly in these cells (Fig. 5g).

Fractionation of CP110-depleted cells revealed p62, HSP70 and pHSP27 in the insoluble fraction (Extended Data Fig. 5m), which demonstrates that protein aggregation occurs in these cells. However, autophagy in CP110-depleted cells was not more strongly induced (Extended Data Fig. 5n), and autophagy inhibition failed to rescue pHSP27 and p62 accumulation (Extended Data Fig. 5o,p). Time course analysis revealed that aggresomes do not grow in size beyond 5 h in CP110-depleted cells (Fig. 5h,i), and pHSP27 remained restricted to the centrioles even after overnight treatment in A-375 cells depleted of CP110, CEP97 or CEP290 (Fig. 5j,k). Together, these data assign a function to the CP110–CEP97–CEP290 module in aggresome formation, upstream of satellites.

### CP110 levels limit aggresome formation in senescent cells

**Fig. 6:**
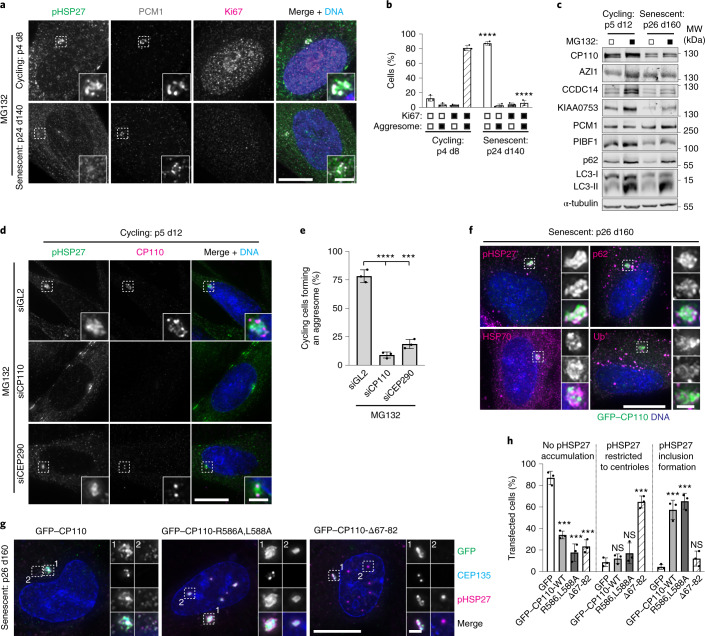
Senescent cells have a reduced capacity to form aggresomes. **a**, Cycling and senescent HFF-1 cells were treated with MG132 and stained as indicated. The number of passages (p) and total number of days in culture (d) are indicated. **b**, Quantitation of aggresome formation in Ki67 positive/negative cycling and senescent HFF-1 cells. **c**, Extracts from cycling and senescent HFF-1 cells treated with or without MG132 were probed as indicated. α-tubulin was used as the loading control. **d**, Cycling HFF-1 cells were treated with control (GL2), CP110 or CEP290 siRNAs, treated with MG132 and stained as indicated. **e**, Quantitation of aggresome formation in cycling HFF-1 cells treated as in **d**. **f**, Senescent HFF-1 cells were transfected with GFP–CP110 and treated with MG132. Cells were stained as indicated. **g**, Senescent HFF-1 cells were transfected with GFP–CP110, GFP–CP110-R586A,L588A or GFP–CP110-Δ67-82 and treated with MG132. Cells were stained for pHSP27. **h**, Quantification of senescent HFF-1 cells treated as in **g** that formed an aggresome. For **b**, **e** and **h**, data are displayed as the mean ± s.d., *n* = 3 independent experiments, and *P* values were calculated by two-tailed unpaired Student’s *t*-test; ****P 0.0001, ***P 0.001. Scale bars, 2 μm (insets of **a**,**d**,**f**,**g**) or 10 μm (**a**,**d**,**f**,**g**). Unprocessed immunoblots, numerical data and *P* values are provided as source data. Source data

**Extended Data Fig. 6:**
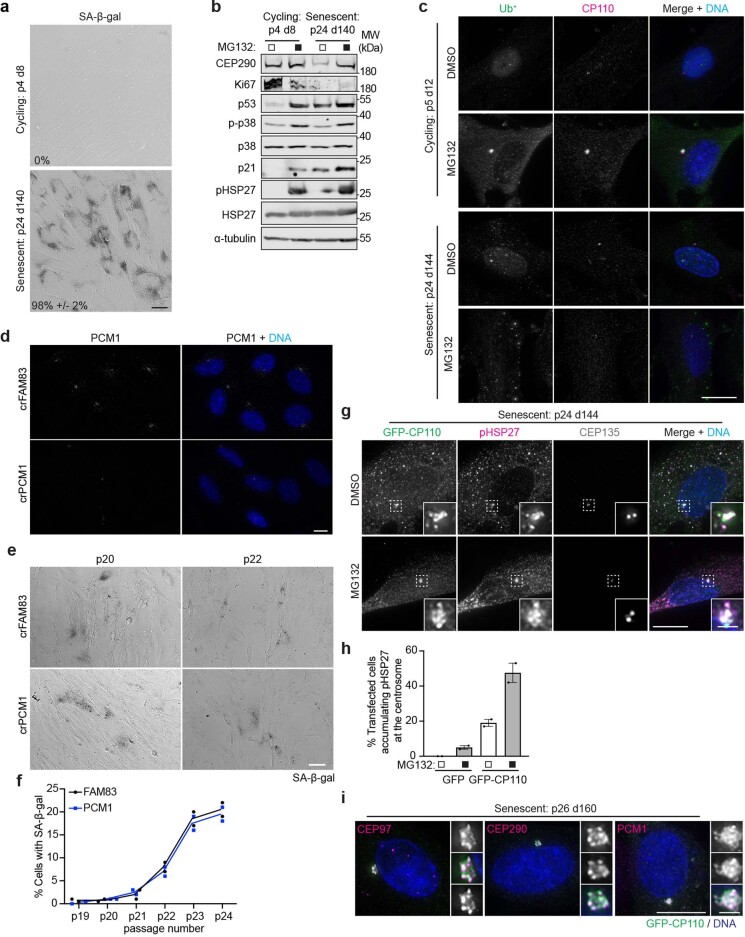
Senescent cells have a reduced capacity to form aggresomes. (**a**) Bright-field image of cycling and senescent HFF-1 cells that were subjected to a senescence associated β-galactosidase (SA-β-gal) assay. The percentage of cells displaying SA-β-gal activity is indicated. Scale bar 10 μm. (**b**) Extracts from cycling and senescent HFF-1 cells treated with or without MG132 were probed for CP110, CEP290, Ki67, p53, phospho-p38, p38, p21, pHSP27 and total HSP27, as indicated. α-tubulin was used as a loading control. (**c**) Cycling and senescent HFF-1 cells were treated with DMSO or MG132, then stained for Ub^+^ and CP110. DNA was stained with DAPI. Scale bar 10 μm. (**d**) crFAM83 and crPCM1 IMR-90 cells were stained for PCM1. Scale bar 10 μm. (**e**) Bright-field image of crFAM83G and crPCM1 IMR-90 cells that were subjected to a senescence associated β-galactosidase (SA-β-gal) assay. Scale bar 20 μm (**f**) Quantification of the percentage of senescent cells as determined by SA-β-gal activity in crFAM83 and crPCM1 IMR-90 cells, *n* = 2 biological replicates. (**g**) Senescent HFF-1 cells were transfected with GFP-CP110 for 48 hours before treatment with MG132. Cells were stained as indicated. Scale bar 10 μm, inset 2 μm. (**h**) Histogram of the percentage of senescent HFF-1 cells transfected with GFP or GFP-CP110 that formed an aggresome. Data displayed as mean and range. *n* = 2 independent experiments. (**i**) Senescent HFF-1 cells were transfected with GFP-CP110, treated with MG132 and stained for CEP97, CEP290 and PCM1. Scale bar 10 μm, inset 2 μm. Unprocessed immunoblots and numerical data are provided as source data. Source data

Protein aggregates accumulate in senescent cells due to decreased proteasomal activity^37,39^. We therefore wondered whether senescent cells utilize the aggresome pathway. Somatic cells in culture undergo replicative senescence after a finite number of divisions^58^. To obtain senescent cells, we grew low-passage primary HFF-1 cells for at least 140 days. Senescence-associated β-galactosidase activity^59^ and increased p53 and p21 levels^60^ confirmed the induction of senescencence (Extended Data Fig. 6a,b). Senescent cells were unable to form aggresomes, which correlated with cells staining negative for the proliferation marker Ki67 (Fig. 6a,b). Aggregates of Ub^+^ were observed throughout the cytoplasm (Extended Data Fig. 6c).

Senescent cells had decreased levels of many satellite proteins, although AZI1 and PCM1 levels were maintained (Fig. 6c), and disruption of PCM1 had no effect on the progression of IMR-90 cells towards senescence (Extended Data Fig. 6d–f). As CP110 levels were reduced, in line with a previous study^61^, we questioned whether this limited aggresome assembly in senescent cells. First, depeletion of CP110 and CEP290 in cycling HFF-1 cells recapitulated our earlier findings (Fig. 6d,e). Conversely, CP110 overexpression rescued aggresome assembly in senescent cells (Extended Data Fig. 6g,h). A possible explanation for this finding is that we observed the formation of green fluorescent protein (GFP)–CP110 aggregates that associated with pHSP27 rather than true aggresomes. However, pHSP27, p62, HSP70 and Ub^+^ (Fig. 6f), along with CEP97, CEP290 and PCM1 (Extended Data Fig. 6i), localization to these structures supported that they are aggresomes. Furthermore, a CP110 construct lacking the CEP290-binding domain (Δ67-82)^32^ failed to rescue aggresome formation, whereas mutation of the CCNF-binding domain (R586A,L588A)^30^ had little effect (Fig. 6g,h). This suggests that CP110 promotes aggresome formation through its interaction with CEP290. Together, these results demonstrate that senescent cells have a reduced capacity to form aggresomes due to reduced CP110 levels.

### Satellites and the CP110 module drive HTT inclusion assembly

**Fig. 7:**
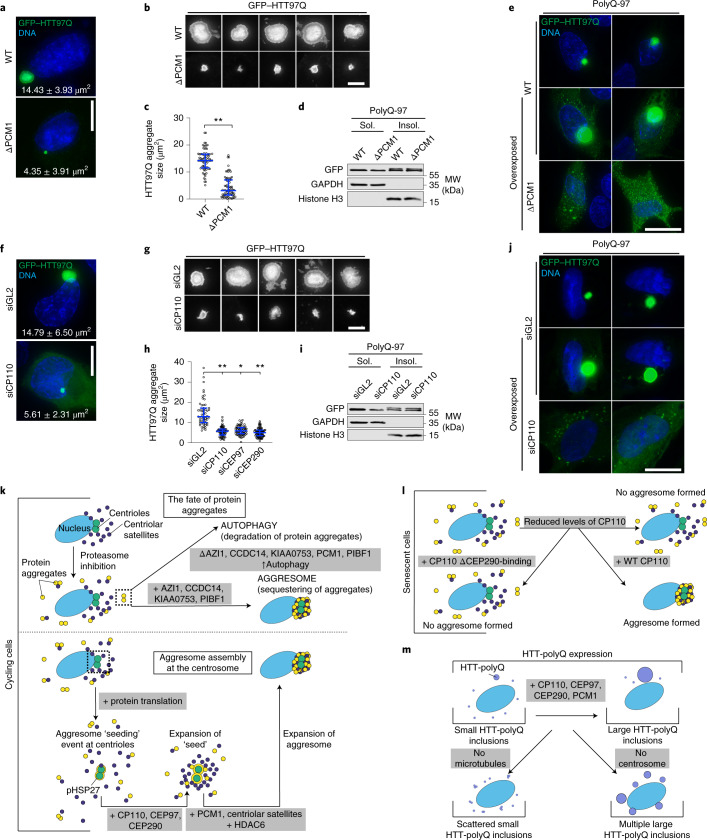
HTT-polyQ inclusion formation requires centriolar satellites and the CP110–CEP97–CEP290 module. **a**, Images of WT and ΔPCM1 cells transfected with GFP–HTT97Q for 24 h. Mean aggregate size ± s.d. is indicated. **b**, Example GFP–HTT97Q aggregates in WT and ΔPCM1 cells. **c**, GFP–HTT97Q aggregate size in WT and ΔPCM1 cells. *n* = 75 cells for each condition over 2 independent experiments. ****P < 0.0001. **d**, Immunoblot of soluble (Sol.) and insoluble (Insol.) fractions from WT and ΔPCM1 cells transfected with GFP–HTT97Q. **e**, GFP–HTT97Q transfected WT and ΔPCM1 cells were imaged with overexposed conditions. **f**, Cells treated with siRNA then transfected with GFP–HTT97Q. Mean aggregate size ± s.d. is indicated. **g**, GFP–HTT97Q aggregates in cells treated with siGL2 or siCP110. **h**, GFP–HTT97Q aggregate size in WT cells treated with the indicated siRNAs. *n* = 57 (siGL2), 62 (siCP110), 68 (siCEP97) and 85 (siCEP290) over 2 independent experiments. ****P < 0.0001. **i**, Immunoblot of fractions from WT cells treated with siGL2 or siCP110, then transfected with GFP–HTT97Q. **j**, GFP–HTT97Q transfected siGL2 or siCP110 cells were imaged with overexposed conditions. **k**, Proteasome inhibition causes protein aggregates to accumulate in the cytoplasm. These aggregates are either directed towards autophagy or sequestered to the aggresome. Without satellites, autophagy is upregulated and becomes the predominant pathway. The earliest event in aggresome formation at the centrosome is the accumulation of pHSP27 on the centrioles and the recruitment of proteins to the aggresome. pHSP27 expansion requires CP110, CEP97 and CEP290, satellites and HDAC6. **l**, Senescent cells have a limited capacity to form aggresomes due to reduced levels of CP110. **m**, Assembly of GFP–HTT97Q aggregates into single inclusions requires the CP110 module, satellites, microtubules and a functional centrosome. For **c** and **h**, bars represent median and interquartile range, with individual values superimposed. *P* values were calculated by two-tailed unpaired Student’s *t*-test. Scale bars, 2 μm (insets of **b**,**g**) or 10 μm (**a**,**e**,**f**,**j**). Unprocessed immunoblots, numerical data and *P* values are provided as source data. Source data

**Extended Data Fig. 7:**
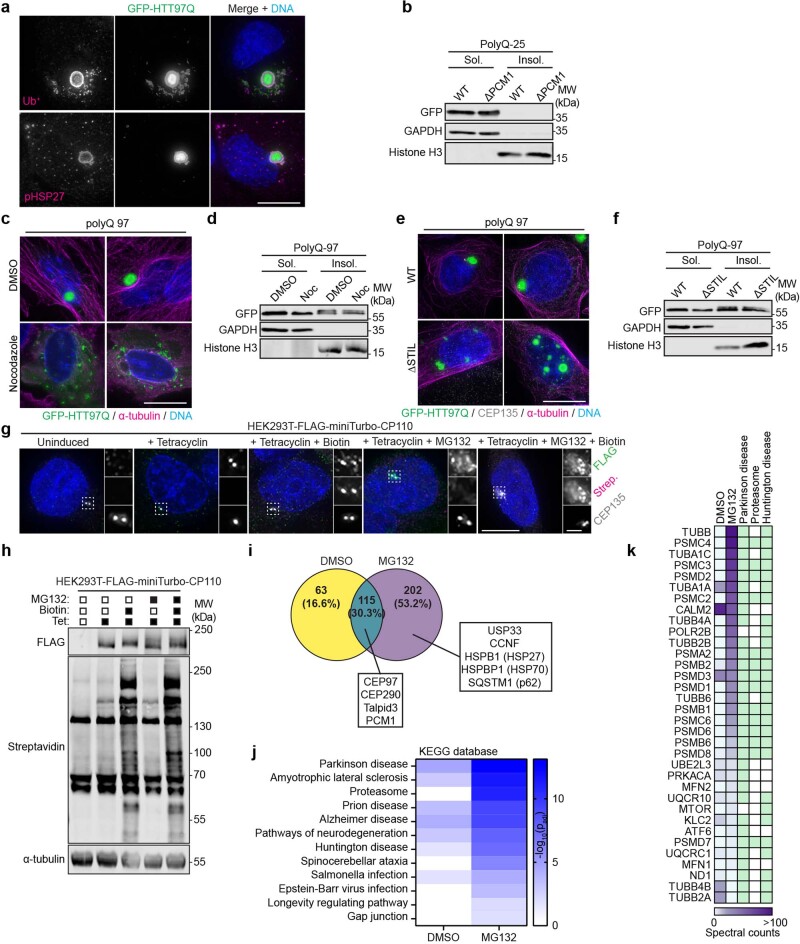
HTT-polyQ inclusion formation requires microtubules and a functional centrosome. (**a**) RPE-1 cells were transiently transfected with GFP-HTT97Q and stained for pHSP27 or Ub^+^. DNA was stained with DAPI. Scale bar 10 μm. (**b**) Immunoblot of soluble and insoluble fractions from WT and PCM1 KO cells transfected with GFP-HTT25Q. GAPDH and Histone H3 were used controls for the soluble and insoluble fractions, respectively. (**c**) Cells were transfected with GFP-HTT97Q for 5 hours, then treated with DMSO or nocodazole for 5 hours before being fixed and stained for GFP and α-tubulin. Scale bar 10 μm. (**d**) Immunoblot of soluble and insoluble fractions from cells prepared as in **c**. GAPDH and Histone H3 were used controls for the soluble and insoluble fractions, respectively. (**e**) WT and STIL KO U-2 OS cells were transiently transfected with GFP-HTT97Q then stained for GFP, CEP135 and α-tubulin. Scale bar 10 μm. (**f**) Immunoblot of soluble and insoluble fractions from cells prepared as in **e**. GAPDH and Histone H3 were used controls for the soluble and insoluble fractions, respectively. (**g**) HEK293T-FLAG-miniTurbo-CP110 cells were treated and stained as indicated. Scale bar 10 μm, 2 μm. (**h**) Immunoblot of HEK293T-FLAG-miniTurbo-CP110 cells treated and probed as indicated. α-tubulin was used as a loading control. (**i**) The number of preys from DMSO- or MG132-treated groups. Preys were defined as detailed in the Methods. (**j**) Functional enrichment analysis was performed with the preys from **i**. using g:Profiler and the KEGG database. (**k**) Spectral counts from the genes which are implicated in Parkinson’s disease, proteasome function or Huntington’s disease are shown. Genes which are related to the respective pathways are marked in green. Unprocessed immunoblots are provided as source data. For **i**,**j**,**k**: the raw mass spectrometry data and analysis giving rise to these panels is available in Supplementary Table 2. Source data

Enrichment of HTT into inclusions is a pathological feature of HD^62^. As HTT interacts with PCM1 through HAP1 (ref. ^41^), we asked whether satellites, or the CP110–CEP97–CEP290 module, are required for a HTT fusion protein containing 97 polyQ repeats (GFP–HTT97Q) to form inclusions. In a proportion of WT cells, GFP–HTT97Q formed a single large inclusion to which pHSP27 and Ub^+^ localized (Extended Data Fig. 7a,b). Measurement of inclusion size in PCM1 KO cells revealed that they were restricted to 4.35 ± 3.91 μm^2^ compared with 14.43 ± 3.93 μm^2^ in WT cells (Fig. 7a–c). However, polyQ-97 still accumulated in the insoluble fraction of PCM1 KO cells (Fig. 7d), which suggests that insoluble aggregates form in these cells. polyQ-25 was used to demonstrate the failure of a non-aggregating polyQ expansion to accumulate in the insoluble fraction (Extended Data Fig. 7b). IF microscopy using overexposed conditions revealed the presence of multiple small aggregates in PCM1 KO cells, thereby demonstrating aggregate formation in these cells, but that they failed to accumulate into a single inclusion. Similar results were obtained for cells depleted of CP110, CEP97 or CEP290 (Fig. 7f–j).

HTT can bind to microtubules and localize to the centrosome^63^, and microtubule depolymerization disrupts inclusion formation^64,65^. In line with this, we observed multiple small polyQ-97 aggregates in cells treated with nocodazole (Extended Data Fig. 7c,d). By contrast, in cells lacking functional centrosomes, multiple larger inclusions formed (Extended Data Fig. 7e,f). Taken together, these results suggest that satellites and the CP110 module participate in the concentration of polyQ-97 aggregates to the centrosome to facilitate the formation of a large single inclusion.

Finally, identification of CP110 proximity interactors during proteasome inhibition revealed an enrichment in pathways associated with protein aggregation diseases, including HD, and the proteasome (Extended Data Fig. 7g–k and Supplementary Table 2). This result therefore supports a role for CP110 in the regulation of proteostasis and aggregation in diseases.

## Discussion

Here, we described the requirement for centriolar satellites and the CP110–CEP97–CEP290 module in aggresome formation (Fig. 7k). In the absence of protein translation, aggresomes were completely blocked. The initial recruitment of pHSP27 to the centrioles seeds aggresome assembly, which expands further depending on the CP110–CEP97–CEP290 module and satellites.

The formation of a ring of pHSP27 around the centrioles suggests this is an early event that lays the foundation for the assembly of aggresomal particles into a single structure (Fig. 7k). This implies that aggregate recruitment is regulated and that aggresome assembly is controlled to enable an ordered structure to form. Previous TEM studies revealed multiple particles loosely associating with each other within aggresomes^2,7,66^, and the images produced in this current study support the notion that aggresomes have higher-order structure. Aggregated proteins are sticky due to exposed hydrophobic residues. Order within the aggresome would bury these residues, which prevents further aggregation and interaction with other cellular components^66,67^.

The exact role that CP110 plays in aggresome assembly remains to be determined. One possibility is that during proteasomal inhibition, components of the CP110 module aggregate and their transport to the centrosome potentiates aggresome formation. Alternatively, as centriolar proteins, they could form a site on the centriole that facilitates aggresome seeding. Indeed, TALPID3 forms ring-like structures on the centriole^57^, and depletion of this CP110 interactor reduced aggresome formation. Notably, CEP120, which also interacts with TALPID3 (ref. ^68^), localized to two ring structures in the centre of the aggresome (Extended Data Fig. 1h), which provides support for the idea that a ring of centriolar proteins resides at the core of aggresome assembly. Finally, as CP110, CEP97 and CEP290 are reported in satellites^33,34^, they may act as adaptors between satellites and aggresomal cargo. Exactly how CP110 functions to target or anchor aggresomal particles to the centrosome will be an important focus of future studies.

Satellites regulate protein stability via the UPS and autophagy^16,17^, and these pathways regulate satellite composition and turnover^17,69–73^, which therefore places satellites at the intersection of these two pathways. The satellite protein KO lines had increased autophagy activity in response to proteasome inhibition, which accounted for the reduction in aggresome size in these cells (presumably because protein aggregates are directed towards autophagy rather than the aggresome). However, depletion of PCM1, which in turn reduces the level of satellites, prevented rescue of aggresome assembly in cells with inhibited autophagy, thereby providing strong evidence for the involvement of satellites in aggresome formation (Fig. 7k).

Sequestration of proteins at the MTOC allows proteins to be transported from throughout the cytoplasm to a central location. Both aggresome assembly and satellite movement depends on dynein-dependent transport along microtubules^7,8,14,15^, so it therefore fits that aggregates and satellites traffic to the aggresome together. Indeed, placement of satellites at the aggresome centre suggests that their disruption would affect aggresome formation by interfering with the homing of aggregate-loaded dynein motors. A key function of satellites is the recruitment of proteins to the centrosome via dynein–dynactin^14^, with several satellite proteins interacting directly with dynein–dynactin. Future studies will focus on identifying the interactions between protein aggregates, satellites and the aggresomal machinery during aggresome assembly.

The localization of satellites around the pHSP27 ring that assembles early during aggresome formation (Fig. 3d) supports their association with the aggresome as it grows (Fig. 7k). PCM1 KO cells assemble a pHSP27 ring, but its expansion is impeded, which suggests that satellites are required for aggresome growth. The degree of expansion that occurred in the absence of satellites depended on the status of autophagic activity. Although slightly more pHSP27 was recruited when autophagy was inhibited in PCM1 KO cells, it was not completely rescued, which suggests that some aggresome expansion can occur in the absence of satellites and autophagy. Conversely, autophagy activation before proteasomal inhibition caused less pHSP27 to assemble. This therefore places satellites at the nexus between centrosomes and proteostasis networks. Strikingly, satellite accumulation did not depend on interactions with aggresomal cargo, as they still accumulated in the pericentrosomal region when HDAC6 was inhibited or HSP27 was depleted.

Ageing is the biggest risk factor associated with neurodegenerative disorders^74^, and protein aggregation, due to decreased proteasomal activity^75^, is a common feature of aged cells^76^. Senescent cells were unable to form aggresomes due to reduced amounts of CP110 (Fig. 7l). CP110 lacking the CEP290 binding domain could not rescue aggresome formation; therefore, these proteins function together to promote aggresome assembly. Satellites and the CP110–CEP97–CEP290 module also facilitated HTT-polyQ inclusion formation (Fig. 7m). The role of proteinaceous inclusions to disease pathology remains unclear. Primary cilia are crucial to neuron function^77^, and cilium structure and function are altered in HD^78^. Expression of HTT-polyQ caused centrosomal accumulation of PCM1 and altered cilium structure^41^, which suggests a link between protein aggregation and cilium function in neurodegeneration. Given the emergent connection between satellites and aggregate processing, and the requirement for satellites in cilia formation and function^17,20^, future studies that elucidate the role of satellites in these processes will provide valuable insight into the pathophysiology of neurodegenerative diseases. Indeed, the identification of proteins associated with aggregation-prone diseases as CP110 interactors raises the tantalizing possibility that targeting CP110 might be a potential therapeutic avenue for such diseases.

## Methods

### Cell culture and drug treatments

All cell lines were cultured in a 5% CO_2_ humidified atmosphere at 37 °C. hTERT RPE-1 (female, human epithelial cells immortalized with hTERT; CRL-4000), A-375 (female, human malignant melanoma epithelial; CRL-1619), BJ-5ta (male, human fibroblasts immortalized with hTERT; CRL-4001), HFF-1 (male, human primary fibroblasts; SCRC-1041), HeLa (female, human adenocarcinoma epithelial; CRM-CCL-2) and U-2 OS (female, human osteosarcoma epithelial; HTB-96) cells from American Type Culture Collection (ATCC) were grown in Dulbecco’s modified Eagle’s medium (DMEM; Life Technologies) supplemented with 10% v/v fetal bovine serum (FBS). IMR-90 (female, human diploid fibroblasts; CCL-186) were cultured in Eagle’s minimum essential medium (EMEM, ATCC) supplemented with 10% v/v FBS. To inhibit the proteasome, cells were treated with 10 μM MG132 (Millipore-Sigma) or 1 μM bortezomib (Santa Cruz Biotechnology) for 5 h. To disrupt microtubules, cells were treated with nocodazole (Millipore-Sigma) at 10 μM or taxol (paclitaxel; Millipore-Sigma) at 5 μM for 2 h before the addition of MG132. Cycloheximide (Enzo Life Sciences) was used to inhibit protein translation at 5 μg ml^–1^ for 30 min before the addition of MG132. ACY-1215 and ACY-738 (Selleck Chemicals) at 50 μM each were used to inhibit HDAC6. To activate autophagy, cells were treated with the mTOR inhibitor KU-0063794 (Selleck Chemicals) at 10 μM for 2 h before the addition of MG132. To inhibit autophagy, cells were treated with 40 μM chloroquine (Millipore-Sigma) for 2 h before MG132 addition. As control, cells were treated with vehicle (dimethylsulfoxide (DMSO)) alone. To verify senescent populations, senescence associated β-galactosidase activity was assayed using a senescence detection kit (Abcam) according to the manufacturer’s instructions.

### Lentiviral methods

To produce lentivirus, 4 × 10^6^ HEK293T (female, human kidney; ATCC, ACS-4500) cells were seeded in a T-75 flask and transfected with the relevant lentiviral transfer vector, 3 μg psPAX2 and 2 μg pCMV-VSV-G using Lipofectamine 3000 (Invitrogen). After 24 h, the growth medium was replaced with fresh medium containing 30% FBS, and the virus supernatant collected after a further 48 h, centrifuged to remove cells and stored at −80 °C. To generate cell lines, 200,000 cells were seeded with varying amounts of virus supernatant and 4 μg ml^–1^ polybrene. Medium was changed after 24 h and cells selected with 3 μg ml^–1^ puromycin (IMR-90) or 600 μg ml^–1^ G418 (RPE-1) until uninfected control cells were dead. Samples with approximately 30% survival or less compared with unselected cells were used to enrich for cells with a single integration event.

### Generation of KO cell lines

To generate hTERT RPE-1 AZI1, CCDC14, KIAA0753, PIBF1 and OFD1 KO cell lines, guide RNA targeting the respective gene (Supplementary Table 1) was selected and transcribed in vitro before being transfected into WT hTERT RPE-1 cells constitutively expressing Cas9 using Lipofectamine RNAiMAX (Invitrogen) according to the manufacturer’s instructions. After 5 days, the transfected cells were diluted to isolate clones. Gene disruption was confirmed by PCR amplification of genomic DNA with the primers detailed in Supplementary Table 1 and sequence analysis using inference of CRISPR edits (Synthego; Supplementary Table 1). Loss of signal of the respective protein was demonstrated by immunoblotting and IF microscopy. To disrupt PCM1 in IMR-90 cells, we used lentivirus-mediated CRISPR gene disruption. *PCM1* guide RNA (Supplementary Table 1) was cloned into pLentiCRISPRv2 plasmid^33,79^, and KOs were generated using the lentiviral methods described above. FAM83G (guide sequence: agcccgggtcacgccgcggt; genomic forward primer: aacaataaagaggccctttgagacgg; genomic reverse primer: ctcatcaggtctttcccgcagatt) was targeted as a control^80^. The U-2 OS STIL KO^81^, RPE-1 CETN2 KO^53^ and RPE-1 FOP KO^54^ have previously been published.

### Clonogenic survival assays

For RPE-1 cells, 250 cells per well were seeded in 6-well dishes. The next day, cells were treated with MG132 for 5 h before drug washout with three changes of medium. After 12 days, plates were rinsed once with PBS, then fixed and stained with 0.5% w/v crystal violet in 20% v/v methanol for 20 min. Plates were washed extensively with water, dried and scanned. Images were segmented using the Trainable Weka Segmentation tool in ImageJ. A new model was built for each replicate. The resulting segmentation image was thresholded and used as a mask to overlay the original image that was inverted and background subtracted using a 50-pixel rolling circle. The colony intensity per well was then measured within the masked region. Data in Extended Data Fig. 4b are presented as the mean and standard deviation of four replicates from two independent experiments.

### RNA-mediated interference

All siRNA transfections were performed using Lipofectamine RNAiMAX (Invitrogen) according to the manufacturer’s instructions. Details of the siRNA oligonucleotides utilized in this study are provided in Supplementary Table 3. hTERT RPE-1 cells were transfected with 20 nM (final concentration) of the respective siRNA for 48 or 72 h, as indicated. Effective knockdown was confirmed by immunoblotting and/or IF microscopy. The GFP–CP110 expression construct has been previously published^61^, and the HSP27 entry clone was a gift from B. Piette and M. Taipale^82^. RNAi-resistant mutants of CP110 and HSP27, and the CP110-Δ67-82 and CP110-R586A,L588A mutants, were generated using site-directed mutagenesis (primers detailed in Supplementary Table 3). To rescue the CP110 RNAi phenotype, cells were transfected with 1 μg of DNA using Lipofectamine 3000 (Invitrogen) 24 h after siRNA transfection. After a further 24 h, cells were treated with MG132 for 5 h. To rescue the HSP27 RNAi phenotype, the siRNA-resistant HSP27 complementary DNA was cloned into a pInducer20, a tetracycline-inducible lentiviral vector^83^, using Gateway technology (Invitrogen). siRNA infection was performed as detailed above for 24 h, before expression of siRNA-resistant HSP27 was induced by the addition of 0.5 μg ml^–1^ tetracycline to the culture medium. Cells were treated with MG132 24 h after induction and collected for analysis.

### Transient transfections

Transfections were performed using Lipofectamine 3000 (Invitrogen) according to the manufacturer’s instructions. In brief, 1 μg plasmid DNA was complexed with Lipofectamine 3000 in serum-free Opti-MEM and added to cells at 70–80% confluency. After 24 h of transfection, cells were treated with MG132 for 5 h before analysis by IF microscopy. The HTT-polyQ constructs (25Q and 97Q) were a gift from G. Hesketh, who modified them from ref. ^84^ to use in human cells, and the FLAG–CEP290 construct has been previously published^33^.

### IF microscopy

Details of the primary and secondary antibodies used for IF microscopy are provided in Supplementary Table 3. For IF microscopy, cells were fixed with ice-cold methanol for 10 min at −20 °C, with the exception of the cells presented in Fig. 6a and Extended Data Fig. 5f, which were fixed with 4% paraformaldehyde for 5 min at room temperature before permeabilization with methanol for 2 min. All cells were blocked with 2% BSA in PBS for 10 min, then incubated with primary antibody in blocking solution for 50 min at room temperature. After washing with PBS, cells were incubated with fluorophore-conjugated secondary antibodies (Supplementary Table 3) and 4,6-diamidino-2-phenylindole (0.1 μg ml^–1^) in blocking solution for 50 min at room temperature. After washing with PBS, coverslips were mounted onto glass slides using ProLong Gold antifade (Molecular Probes). Cells were imaged using a DeltaVision Elite high-resolution imaging system equipped with a sCMOS 2,048 × 2,048 pixel camera (GE Healthcare). *Z*-stacks (0.2-μm step) were collected using a ×60, 1.42 NA plan apochromat oil-immersion objective (Olympus) and deconvolved using softWoRx (v.6.0, GE Healthcare). Images are shown as maximum intensity projections. The images presented in Figs. 1g and 3d and Extended Data Fig. 1h were captured using a Nikon CSU-W1 SoRa spinning disk super-resolution system equipped with a LUN-F XL laser unit and a Hamamatsu FusionBT sCMOS camera. *Z*-stacks (0.125-μm step) were collected using a ×60, 1.4 NA plan apochromat λ oil-immersion objective (Nikon) and deconvolved in NIS-Elements (Nikon). Images are presented as maximum intensity projections. All images were processed for publication using Adobe Photoshop 2020 and figures prepared using Adobe Illustrator 2020.

### Colocalization analysis from super-resolution images

Super-resolution SoRa images were used for colocalization analysis. Each image was cropped using a 7.5 × 7.5 μm area centred around the aggresome. The Pearson’s correlation co-efficient for each image pair was determined using the JACoP plug-in for ImageJ^85^. At least ten images were used for each pair of antibody stainings.

### Automated quantitative image analysis

Images were analysed using CellProfiler^86^ (v.3.0; https://cellprofiler.org) and R (https://www.r-project.org). We chose pHSP27 staining as the marker for measurement of aggresome size due to its low background in control-treated cells and distinct signal around the centrosome in cells treated with MG132. Knockdown of HSP27 demonstrated the specificity of this signal. For pHSP27 analysis, centrosomes were identified using the CEP135 signal. Foci closer than 8 pixels were merged, and all centrosomes identified were shrunk to a single pixel. The aggresome area was found using the centrosome coordinates as seed points and expanding outwards on the pHSP27 channel using the Watershed algorithm. The lower quartile of each pHSP27 image was used as a background subtraction value before the total pHSP27 intensity in the aggresome area was determined. The results from two independent experiments, each using at least 291 cells, were pooled and plotted (source data are provided). Box-and-whisker plots show the median, upper and lower quartiles (boxes), and 1.5× the interquartile range (whiskers), with individual data points plotted. Data distribution was assumed to be normal, but this was not formally tested. The data were analysed using a Kruskal–Wallis analysis of variance (ANOVA) to determine whether at least one distribution was statistically different from the others. A post-hoc Dunn test was then performed to assess pairwise differences and to calculate *P* values. To measure centriolar satellites, we used PCM1 staining, as per convention of the field. For PCM1 analysis, the same images were analysed and centrosomes were identified as above using the CEP135 signal (or CETN2 in the case of Extended Data Fig. 3j). The centrosome points were expanded 13 pixels to define the ‘inner PCM’ area that approximates the average size of an aggresome. The inner region was further expanded 100 pixels to create an annulus defining the ‘outer PCM’ area that generally encompassed the entire cell and were non-overlapping between cells. The inner satellites were segmented using an adaptive Otsu method and the outer satellites segmented with a global Otsu algorithm. The (*x*,*y*) position for every satellite was calculated relative to the cell centrosome position. We assumed that the cells were rotationally similar and overlaid all cells in each condition centred on the centrosome. The PCM1 signal was background subtracted using the lower quartile of each image and further subtracted based on PCM1 KO images to correct for spurious objects detected in the absence of PCM1. The centroid of each satellite was weighted by the total corrected PCM1 intensity of the object and visualized using a hexbin plot normalized to the total number of cells and replicates. On the inner satellite plots, the percentage of total PCM1 intensity within this pericentrosomal region is displayed.

### Soluble/insoluble fractionation

The following protocol was adapted from that used by Runwal et al.^87^. Cells grown in 60-mm dishes and treated as indicated were washed twice with PBS then scraped into 360 μl RIPA (50 mM Tris-HCl pH 8, 1 mM EDTA, 150 mM NaCl, 0.25% sodium-deoxycholate, 0.1% SDS and 1% NP40) supplemented with complete mini protease inhibitor cocktail (Sigma) and 1:100 phosphatase inhibitor cocktail 3 (Sigma). The lysates were then passed through 20 gauge needles 8 times, incubated on ice for 15 min and then centrifuged at 16,000*g* for 15 min at 4 °C. The supernatant was collected as the soluble fraction, to which 200 μl of 2× SDS–PAGE buffer was added before boiling at 95 °C for 5 min. The pellet was washed once with 250 μl RIPA and centrifuged again for 5 min at 4 °C. The supernatant was discarded and the pellet resuspended in 72 μl RIPA containing 2 M urea (Sigma). An equal volume of 2× SDS–PAGE buffer was added, and the insoluble fractions boiled at 95 °C for 5 min. All samples were stored at −80 °C before being re-boiled for 5 min and resolved by SDS–PAGE and subjected to immunoblotting as detailed below.

### Immunoblotting

Details of the primary and secondary antibodies used for immunoblotting are provided in Supplementary Table 3. For immunoblotting, total cell lysates were collected in 2× SDS–PAGE buffer and treated with benzonase nuclease (Millipore-Sigma). Proteins were separated on SDS–PAGE gels and transferred to polyvinylidenedifluoride membrane (Amersham Hybond P, Cytiva). Membranes were blocked for 30 min with 5% w/v milk powder (BioShop) in TBS, then incubated with primary antibodies in 5% w/v milk powder-TBST (TBS, 0.1% Tween-20) at 4 °C overnight. Following washing in TBST, blots were incubated with IRDye-conjugated secondary antibodies (LI-COR) in the dark for 1 h at room temperature. Blots were then washed three times in TBST and once in TBS, before imaging on a LI-COR Odyssey CLx Infrared Imager. Unprocessed blots are presented as source data.

### TEM

For thin-section TEM, hTERT RPE-1 cells were grown in 10-cm dishes and treated with DMSO or MG132 for 5 h. Cells were then pelleted and washed twice with PBS, before primary fixation in 2% glutaraldehyde and 2% paraformaldehyde in 0.1 M sodium cacodylate buffer overnight at 4 °C, and secondary fixation in 2% osmium tetroxide. Samples were dehydrated through an ethanol gradient, followed by propylene oxide and embedded in EMbed 812 resin (Electron Microscopy Sciences). Ultra-thin sections were cut on a RMC MT6000 ultramicrotome and stained with 2% uranyl acetate in 70% methanol and aqueous lead citrate. Sections were viewed on a FEI Tecnai 20 transmission electron microscope.

### Generation of Flp-In T-REx 293 cells for miniTurbo proximity labelling

Proximity labelling was carried out utilizing miniTurbo. Full-length *CP110* cDNA was cloned into a pcDNA5-FRT/TO-miniTurbo-FLAG destination vector via the Gateway cloning system. Using the Flp-In system, HEK293 cells stably expressing miniTurbo-FLAG (control) and miniTurbo-FLAG–CP110 were generated. After selection with 200 μg ml^–1^ hygromycin B, 5 × 150-cm^2^ plates of subconfluent (~70%) cells per biological replicate were incubated in complete medium supplemented with 1 μg ml^–1^ tetracycline (BioShop) to induce expression of the miniTurbo construct. The next day, cells were treated with DMSO or MG132 as indicated for 5 h, with 50 μM biotin (BioShop) added for the final hour. Cells were scraped into PBS and pelleted at 500*g* for 5 min at 4 °C. Cell pellets were washed twice with PBS and then snap-frozen.

### Sample preparation for mass spectrometry

Each cell pellet was resuspended in 10 ml of lysis buffer (50 mM Tris–HCl pH 7.5, 150 mM NaCl, 1 mM EDTA, 1 mM EGTA, 1% Triton X-100, 0.1% SDS, 1:500 protease inhibitor cocktail (Sigma-Aldrich) and 1:1,000 benzonase nuclease (Novagen)), incubated on an end-over-end rotator at 4 °C for 1 h, briefly sonicated to disrupt any visible aggregates and then centrifuged at 45,000*g* for 30 min at 4 °C. The supernatant was transferred to a fresh 15-ml conical tube. A total of 30 μl of packed, pre-equilibrated streptavidin sepharose beads (GE Healthcare) was added and the mixture was incubated for 3 h at 4 °C with end-over-end rotation. Beads were pelleted by centrifugation at 820*g* for 2 min and transferred with 1 ml of lysis buffer to a fresh Eppendorf tube. Beads were washed once with 1 ml lysis buffer and twice with 1 ml 50 mM ammonium bicarbonate (pH 8.3). Beads were transferred in ammonium bicarbonate to a fresh centrifuge tube and washed twice more with 1 ml ammonium bicarbonate buffer. Tryptic digestion was performed by incubating the beads with 1 μg MS-grade TPCK trypsin (Promega) dissolved in 200 μl 50 mM ammonium bicarbonate (pH 8.3) overnight at 37 °C. The following morning, 0.5 μg MS-grade TPCK trypsin was added, and beads were incubated for an additional 2 h at 37 °C. Beads were pelleted by centrifugation at 2,000*g* for 2 min, and the supernatant transferred to a fresh Eppendorf tube. Beads were washed twice with 150 μl of 50 mM ammonium bicarbonate, and these washes were pooled with the first eluate. The sample was lyophilized and resuspended in buffer A (0.1% formic acid). One-fifth of the sample was analysed per mass spectometry (MS) run.

### MS analysis

MS/MS was done as previously described^88^. In brief, high-performance liquid chromatography was conducted using a 2 cm pre-column (Acclaim PepMap, 50 mm × 100 μm inner diameter) and 50 cm analytical column (Acclaim PepMap, 500 mm × 75 μm diameter, C18, 2 μm, 100 Å; Thermo Fisher Scientific), running a 120-min reversed-phase buffer gradient at 225 nl min^–1^ on a Proxeon EASY-nLC 1000 pump in-line with a Thermo Q Exactive HF Quadrupole-Orbitrap mass spectrometer. A parent ion scan was performed using a resolving power of 60,000, then up to the 20 most intense peaks were selected for MS/MS (minimum ion count of 1,000 for activation), using higher energy collision-induced dissociation (HCD) fragmentation. Dynamic exclusion was activated such that MS/MS of the same *m/z* (within a range of 10 ppm; exclusion list size of 500) detected twice within 5 s was excluded from analysis for 15 s. For protein identification, Thermo .RAW files were converted to the .mzXML format using ProteoWizard^89^, then searched using X!Tandem^90^ and Comet^91^ against the human Human RefSeq Version 45 database (containing 36,113 entries). Search parameters specified a parent ion mass tolerance of 10 ppm and a MS/MS fragment ion tolerance of 0.4 Da, with up to 2 missed cleavages allowed for trypsin. Variable modifications of +16 at methionine and tryptophan residues, +32 at methionine and tryptophan residues, +42 at the amino terminus and +1 at asparagine and glutamine residues were allowed.

### Protein identification and functional enrichment analysis

Both SAINT express (v.3.6.1)^92^ and MiST^93^ analyses were performed with proteins detected with an iProphet Probability of ≥0.9 and number of unique peptides ≥2. High-confidence interactors were defined when SAINT’s BFDR value was <0.01 and MiST score was >0.75. Functional enrichment analysis was performed with the high-confidence interactors using g:Profiler with the KEGG Pathway database^94,95^. Terms with adjusted enrichment *P* < 0.05 are presented.

### Statistics and reproducibility

All experiments were independently repeated at least twice, with similar results obtained. No statistical method was used to predetermine the sample size. No data were excluded from the analyses. For statistical analysis, data distribution was assumed to be normal, but this was not formally tested; individual data points are presented on all plots to show data distribution. Samples were not randomized to specific treatment groups because samples depended on specific genetic cell lines or knockdown conditions; therefore, randomization was not appropriate. Data collection and analysis were not performed blind to the conditions of the experiments. Source data and exact sample sizes are provided as source data files for each figure. Statistical analyses were performed using two-tailed unpaired Student’s *t*-tests and significance was assumed by *P* < 0.05. *P* values are defined as follows: ****P < 0.0001, ***P < 0.001, **P < 0.01, *P < 0.05, NS, not significant. Statistical analysis of aggresome size was performed using Kruskal–Wallis ANOVA with post-hoc Dunn test as detailed in the ‘Automated quantitative image analysis’ section.

Data presented in Figs. 1c, 4j, 5k and 6b,e,h and Extended Data Figs. 1c,e, 4b and 5j were collected over three independent experiments. Data presented in Figs. 1h, 2b,d,f,h,j,k, 3b,c,g,h, 4b,d,h, 5b,d,f,i and 7c,h and Extended Data Figs. 1i, 2d,f, 3c,g,k, 4e, 5e,o and 6f,h were collected over two independent experiments. Immunoblots presented in Figs. 4a,d, 6c and 7d,i and Extended Data Figs. 2c,g, 3a,f,i, 4c,d,g, 5a–c,h,m,n, 6b and 7,b,d,f,h are representative of at least two independent experiments. Micrographs presented in Figs. 1a,b,d–f, 2a,c,e,g,i,l, 3a,d–f,i, 4c,f,g,i, 5a,c,e,g,h,j, 6a,d,f,g and 7e,j and Extended Data Figs. 1a,b,d,g, 2b,e,h–k, 3b,d,e,h,k, 4f, 5d,f,g,i,k,l,p, 6c,d,i and 7a,c,e,g are representative of at least three independent experiments. Micrographs presented in Figs. 1g and 7a,b,f,g and Extended Data Figs. 1f,g and, 6g are representative of two independent experiments.

### Reporting Summary

Further information on research design is available in the Nature Research Reporting Summary linked to this article.

## Online content

Any methods, additional references, Nature Research reporting summaries, source data, extended data, supplementary information, acknowledgements, peer review information; details of author contributions and competing interests; and statements of data and code availability are available at 10.1038/s41556-022-00869-0.

## Supplementary information

Reporting Summary

Peer Review Information

Supplementary Tables File contains a workbook with multiple tabs assigned to Supplementary Tables 1, 2 and 3.

## Data Availability

All mass spectrometry data regarding CP110 proximity interactors have been deposited in the MassIVE (https://massive.ucsd.edu) repository under accession MSV000088076. The previously published KEGG Pathway database^95^ (https://www.genome.jp/kegg/pathway.html) that was re-analysed here was accessed via the web-based toolset gProfiler^94^ (https://biit.cs.ut.ee/gprofiler/gost, version e104_eg51_p15_3922dba, updated on 2021-05-07). All other data supporting the findings of this study are available from the corresponding authors on reasonable request. Source data are provided with this paper.

## Source data

Source Data Fig. 1 Statistical source data.
Source Data Fig. 2 Statistical source data.
Source Data Fig. 3 Statistical source data.
Source Data Fig. 4 Statistical source data.
Source Data Fig. 4 Unprocessed western blots.
Source Data Fig. 5 Statistical source data.
Source Data Fig. 6 Statistical source data.
Source Data Fig. 6 Unprocessed western blots.
Source Data Fig. 7 Statistical source data.
Source Data Fig. 7 Unprocessed western blots.
Source Data Extended Data Fig. 1 Statistical source data.
Source Data Extended Data Fig. 2 Statistical source data.
Source Data Extended Data Fig. 2 Unprocessed western blots.
Source Data Extended Data Fig. 3 Statistical source data.
Source Data Extended Data Fig. 3 Unprocessed western blots.
Source Data Extended Data Fig. 4 Statistical source data.
Source Data Extended Data Fig. 4 Unprocessed western blots.
Source Data Extended Data Fig. 5 Statistical source data.
Source Data Extended Data Fig. 5 Unprocessed western blots.
Source Data Extended Data Fig. 6 Statistical source data.
Source Data Extended Data Fig. 6 Unprocessed western blots.
Source Data Extended Data Fig. 7 Unprocessed western blots.

## Code availability

The analysis pipelines for measurement of aggresomes and centriolar satellites were generated using CellProfiler^86^ (https://cellprofiler.org). The pipelines are available on Zenodo (https://www.zenodo.org/record/5834143; 10.5281/zenodo.5834143) or from the corresponding authors on reasonable request.
