# Supplementary material for: Aggresome assembly at the centrosome is driven by CP110–CEP97–CEP290 and centriolar satellites
Source: Nat Cell Biol. 2022 Apr 11;24(4):483–96. doi: 10.1038/s41556-022-00869-0 (PMC9033585; doi:10.1038/s41556-022-00869-0)
Supplement: Supplementary file 1 — Reporting Summary [file 41556_2022_869_MOESM1_ESM.pdf]

nature research

Corresponding author(s): Dr. Laurence Pelletier and Dr. Suzanna Prosser

Last updated by author(s): Jan 10, 2022

Reporting Summary

Nature Research wishes to improve the reproducibility of the work that we publish. This form provides structure for consistency and transparency in reporting. For further information on Nature Research policies, see our Editorial Policies and the Editorial Policy Checklist.

Statistics

For all statistical analyses, confirm that the following items are present in the figure legend, table legend, main text, or Methods section.

n/a	Confirmed	
☐	☒	The exact sample size (*n*) for each experimental group/condition, given as a discrete number and unit of measurement
☐	☒	A statement on whether measurements were taken from distinct samples or whether the same sample was measured repeatedly
☐	☒	The statistical test(s) used AND whether they are one- or two-sided<br>*Only common tests should be described solely by name; describe more complex techniques in the Methods section.*
☐	☒	A description of all covariates tested
☐	☒	A description of any assumptions or corrections, such as tests of normality and adjustment for multiple comparisons
☐	☒	A full description of the statistical parameters including central tendency (e.g. means) or other basic estimates (e.g. regression coefficient) AND variation (e.g. standard deviation) or associated estimates of uncertainty (e.g. confidence intervals)
☐	☒	For null hypothesis testing, the test statistic (e.g. *F*, *t*, *r*) with confidence intervals, effect sizes, degrees of freedom and *P* value noted<br>*Give P values as exact values whenever suitable.*
☒	☐	For Bayesian analysis, information on the choice of priors and Markov chain Monte Carlo settings
☒	☐	For hierarchical and complex designs, identification of the appropriate level for tests and full reporting of outcomes
☐	☒	Estimates of effect sizes (e.g. Cohen's *d*, Pearson's *r*), indicating how they were calculated

Our web collection on statistics for biologists contains articles on many of the points above.

Software and code

Policy information about availability of computer code

Data collection	Image J (https://imagej.net; v 1.53); CellProfiler (https://cellprofiler.org; v 4.2.1); R (https://www.r-project.org; v 3.6.1); RStudio (https://www.rstudio.com; v1.1.442); softWoRx (v6.0); NIS-Elements (v 5.30.02); GraphPad/Prism (v 9)
Data analysis	The CellProfiler pipelines developed for this study have been deposited on Zenodo URL: https://www.zenodo.org/record/5834143; DOI: 10.5281/zenodo.5834143

For manuscripts utilizing custom algorithms or software that are central to the research but not yet described in published literature, software must be made available to editors and reviewers. We strongly encourage code deposition in a community repository (e.g. GitHub). See the Nature Research guidelines for submitting code & software for further information.

Data

Policy information about availability of data

All manuscripts must include a data availability statement. This statement should provide the following information, where applicable:

- Accession codes, unique identifiers, or web links for publicly available datasets
- A list of figures that have associated raw data
- A description of any restrictions on data availability

Source data for all numerical data in this article have been provided in the Source Data file. All MS data has been deposited in the MassIVE (massive.ucsd.edu) repository under accession MSV000088076. The previously published KEGG PATHWAY database (genome.jp/kegg/pathway.html) that was re-analysed here was accessed via the web-based toolset gProfiler92 (biit.cs.ut.ee/gprofiler/gost, version e104_eg51_p15_3922dba, updated on 2021-05-07). All data generated and analyzed during this study are included in this published article (and its supplementary information files).

Field-specific reporting

Please select the one below that is the best fit for your research. If you are not sure, read the appropriate sections before making your selection.

☒ Life sciences ☐ Behavioural & social sciences ☐ Ecological, evolutionary & environmental sciences

For a reference copy of the document with all sections, see nature.com/documents/nr-reporting-summary-flat.pdf

Life sciences study design

All studies must disclose on these points even when the disclosure is negative.

Sample size	No statistical methods were used to pre-determine sample sizes but sample sizes are similar to those reported in previous publications. All experiments were independently repeated at least twice with similar results. Presented immuno-fluorescence images and immuno-blots are representative of 2 - 3 independent experiments.
Data exclusions	No data were excluded.
Replication	2 - 3 replicates were performed per experiment as stated. All attempts at replication were successful.
Randomization	There was no randomization of samples to specific treatment groups because samples depended upon specific genetic cell lines or knockdown conditions so randomization was not appropriate.
Blinding	Data collection and analysis was blinded between two investigators.

Reporting for specific materials, systems and methods

We require information from authors about some types of materials, experimental systems and methods used in many studies. Here, indicate whether each material, system or method listed is relevant to your study. If you are not sure if a list item applies to your research, read the appropriate section before selecting a response.

Materials & experimental systems

n/a	Involved in the study	
☐	☒	Antibodies
☐	☒	Eukaryotic cell lines
☒	☐	Palaeontology and archaeology
☒	☐	Animals and other organisms
☒	☐	Human research participants
☒	☐	Clinical data
☒	☐	Dual use research of concern

Methods

n/a	Involved in the study	
☒	☐	ChIP-seq
☒	☐	Flow cytometry
☒	☐	MRI-based neuroimaging

Antibodies

Antibodies used

α-tubulin (DM1A), Sigma, T6199
γ-tubulin (GTU-88), Sigma, T6557
AZI1(CEP131), Abcam, ab84864
CCDC14, GeneTex, GTX120754
CCNF, Santa Cruz, sc-952
CETN2 (20H5), Millipore, 04-1624
CEP72, Proteintech, 19928-1-AP
CEP97, Bethyl, A301-945A
CEP120, Betleja et al., 2018
CEP135, Bird and Hyman, 2008
CEP192, Bethyl, A302-324A
CEP215 (CDK5RAP2), Bethyl, IHC-00063
CEP290, Novus Biologicals, NB100-86991
CP110, Proteintech, 12780-1-AP
Dynein HC (C-5), Santa Cruz, sc-514579
Dynein IC1/2 (74-1), Santa Cruz, sc-13524
FLAG, Sigma, F7425
FLAG, Sigma, F3165
FGFR1OP (FOP), Proteintech, 11343-1-AP
GAPDH, Sigma, G9545
GFP (7.1 and 13.1), Roche, 11.814.460.001
GFP, homemade

HAP1 (G-3), Santa Cruz, sc-166245
HAUS6, Lawo et al., 2009,
HDAC6, Millipore, 07-732
Histone H3 K4me3 (MAb-152-050), Diagenode, C15200152
Histone H3 acetyl K27, Abcam, ab4729
HSP27 (F-4), Santa Cruz, sc-13132
p-HSP27 (B-3), Santa Cruz, sc-166693
p-HSP27, Cell Signaling, 9709
HSP40 (DNAJB1), Cell Signaling, 4868
HSP40 (DNAJC7), Abcam, ab179830
HSP70/HSC70 (W27), Santa Cruz, sc-24
HSP90 (C45G5), Cell Signaling, 4877
Ki67 (SP6), Abcam, ab16667
KIAA0753, Abcam, ab121736
MIB1, Sigma, M5948
mono- and poly-ubiquitinylated conjugates (FK2), Enzo, BML-PW8810-0100
NEDD1 (H-300), Santa Cruz, SC-67263
OFD1, Sigma-Atlas , HPA031103
p21 (F-5), Santa Cruz, sc-6246
p38α/β (A-12), Santa Cruz, sc-7972
p-p38 (E-1), Santa Cruz, sc-166182
p53 (DO-1), Santa Cruz, sc-126
p62 (SQSTM) (2C11), Abcam, ab56416
PCM1, Bethyl, A301-149A
PCM1, Santa Cruz, sc-50164
Pericentrin, Abcam, ab4448
PIBF1 (CEP90), Proteintech, 14413-1-AP
Anti-polyglutamylation modification (GT335), Adipogen, AG-20B-0020-C100
SAS6 (91.390.21), Santa Cruz, sc-81431
STIL, Abcam, ab89314
Talpid3, Proteintech, 24421-1-AP
USP33, Bethyl, A300-925A
Vimentin (V9), Sigma, V6389

Alexa Fluor 647 Phalloidin labelled peptide/protein Invitrogen A22287
anti-goat Alexa 488/594/647 donkey Life Technologies A-11055/A-11058/A-21447
anti-mouse Alexa 488/594/647 donkey Life Technologies A-21202/A-21203/A-31571
anti-rabbit Alexa488/594/647 donkey Life Technologies A-21206/A-21207/A-31573
anti-rat Alexa594 donkey Life Technologies A-21209
anti-Streptavidin Alexa594 donkey Life Technologies S-11227
anti-rabbit IRDye 800CW goat LI-COR 926-32211
anti-mouse IRDye 680RD goat LI-COR 926-68070
anti-Streptavidin IRDye 680RD goat LI-COR 926-68079

Validation

Antibodies against AZI1(CEP131), CCDC14, CCNF, CEP97, CEP290, CP110, HSP27, p-HSP27, KIAA0753, MIB1, OFD1, PCM1, PIBF1 (CEP90), Talpid3 and USP33 were validated by immunofluorescence and/or immunoblot of cells depleted of the corresponding protein by siRNA or CRISPR/Cas9 mediated knockdown. CEP135, CEP120 and HAUS6 were validated in the referenced source publications. The remaining antibodies are extensively cited in the literature and/or validated by the manufacturer.

α-tubulin (DM1A), Sigma, T6199
validation reference: https://www.sigmaaldrich.com/CA/en/product/sigma/t6199
https://www.sigmaaldrich.com/specification-sheets/260/142/T6199-BULK________SIGMA____.pdf

γ-tubulin (GTU-88), Sigma, T6557
validation reference: https://www.sigmaaldrich.com/CA/en/product/sigma/t6557
https://www.sigmaaldrich.com/specification-sheets/173/099/T6557-BULK________SIGMA____.pdf

AZI1(CEP131), Abcam, ab84864
validation reference: https://www.abcam.com/azi1-antibody-ab84864.html
WB/IF: KO in RPE-1 human cells; ED Fig 3a,b

CCDC14, GeneTex, GTX120754
validation reference: https://www.genetex.com/Product/Detail/CCDC14-antibody-C2C3-C-term/GTX120754
WB/IF: KO in RPE-1 human cells; ED Fig 3a,b

CCNF, Santa Cruz, sc-952
validation reference: https://www.scbt.com/p/cyclin-f-antibody-c-20
WB: siRNA in RPE-1 human cells; ED Fig. 5 b

CETN2 (20H5), Millipore, 04-1624
validation reference: https://www.emdmillipore.com/CA/en/product/Anti-Centrin-Antibody-clone-20H5,MM_NF-04-1624
WB/IF: KO in RPE-1 human cells - https://rupress.org/jcb/article/208/6/693/38101/Centrin2-regulates-CP110-removal-in-primary-cilium - Figure 3 b, c

CEP72, Proteintech, 19928-1-AP

validation reference: https://www.ptglab.com/products/CEP72-Antibody-19928-1-AP.htm

CEP97, Bethyl, A301-945A
validation reference: https://www.fortislife.com/products/primary-antibodies/rabbit-anti-cep97-antibody/BETHYL-A301-945
WB: siRNA in RPE-1 human cells; ED Fig. 5 b

CEP120, Betleja et al., 2018
validation reference: https://elifesciences.org/articles/35439
Secificity for Cep120 by immunoblot analysis of whole cell lysates (used at a dilution of 1:5,000-1:10,000; Figure 1—figure supplement 1) and by immunofluorescence microscopy (used at 1:2,000; Figures 1–7)

CEP135, Bird and Hyman, 2008
validation reference: https://rupress.org/jcb/article/182/2/289/34985/Building-a-spindle-of-the-correct-length-in-human

CEP192, Bethyl, A302-324A
validation reference: https://www.fortislife.com/products/primary-antibodies/rabbit-anti-cep192-antibody/BETHYL-A302-324

CEP215 (CDK5RAP2), Bethyl, IHC-00063
validation reference: https://www.fortislife.com/products/primary-antibodies/rabbit-anti-cdk5rap2-ihc-antibody/BETHYL-IHC-00063

CEP290, Novus Biologicals, NB100-86991
validation reference: https://www.novusbio.com/products/cep290-antibody_nb100-86991
WB: siRNA in RPE-1 human cells; ED Fig. 5 b

CP110, Proteintech, 12780-1-AP
validation reference: https://www.ptglab.com/products/CP110-Antibody-12780-1-AP.htm
WB: siRNA in RPE-1 human cells; ED Fig. 5 b

Dynein HC (C-5), Santa Cruz, sc-514579
validation reference: https://www.scbt.com/p/dynein-hc-antibody-c-5

Dynein IC1/2 (74-1), Santa Cruz, sc-13524
validation reference: https://www.scbt.com/p/dynein-ic1-2-cytosolic-antibody-74-1

FLAG, Sigma, F7425
validation reference: https://www.sigmaaldrich.com/CA/en/product/sigma/f7425
https://www.sigmaaldrich.com/specification-sheets/447/086/F7425-BULK________SIGMA____.pdf

FLAG, Sigma, F3165
validation reference: https://www.sigmaaldrich.com/CA/en/product/sigma/f3165
https://www.sigmaaldrich.com/specification-sheets/120/274/F3165-BULK.pdf

FGFR1OP (FOP), Proteintech, 11343-1-AP
validation reference: https://www.ptglab.com/products/FGFR1OP-Antibody-11343-1-AP.htm

GAPDH, Sigma, G9545
validation reference: https://www.sigmaaldrich.com/CA/en/product/sigma/g9545
https://www.sigmaaldrich.com/specification-sheets/421/966/G9545-BULK________SIGMA____.pdf

GFP (7.1 and 13.1), Roche, 11.814.460.001
validation reference: https://www.sigmaaldrich.com/deepweb/assets/sigmaaldrich/product/documents/294/951/11814460001bul.pdf

HAP1 (G-3), Santa Cruz, sc-166245
validation reference: https://www.scbt.com/p/hap1-antibody-c-3

HAUS6, Lawo et al., 2009
validation reference: https://doi.org/10.1016/j.cub.2009.04.033
WB/IF: esiRNA transfected HeLa cells; Fig S7 A,B

HDAC6, Millipore, 07-732
validation reference: https://www.emdmillipore.com/CA/en/product/Anti-HDAC6-Antibody-CT,MM_NF-07-732
Immunoblot Analysis: 0.1-1.0µg/ml of this lot detected HDAC6 in RIPA lysates from Jurkat, A431 and 3T3/A31 cells

Histone H3 K4me3 (MAb-152-050), Diagenode, C15200152
validation reference: https://www.diagenode.com/files/products/antibodies/Datasheet_H3K4me3_C15200152.pdf

Histone H3 acetyl K27, Abcam, ab4729
validation reference: https://www.abcam.com/histone-h3-acetyl-k27-antibody-chip-grade-ab4729.html

HSP27 (F-4), Santa Cruz, sc-13132
validation reference: https://www.scbt.com/p/hsp-27-antibody-f-4
WB: siRNA RPE-1 human cells – ED Fig 2C

p-HSP27 (B-3), Santa Cruz, sc-166693

validation reference: https://www.scbt.com/p/p-hsp-27-antibody-b-3
IF: siRNA RPE-1 human cells – ED Fig 2b

p-HSP27, Cell Signaling, 9709
validation reference: https://www.cellsignal.com/products/primary-antibodies/phospho-hsp27-ser82-d1h2f6-xp-rabbit-mab/9709
WB: siRNA RPE-1 human cells – ED Fig 2C

HSP40 (DNAJB1), Cell Signaling, 4868
validation reference: https://www.cellsignal.com/products/primary-antibodies/hsp40-antibody/4868

HSP40 (DNAJC7), Abcam, ab179830
validation reference: https://www.abcam.com/dnajc7-antibody-epr13349-n-terminal-ab179830.html

HSP70/HSC70 (W27), Santa Cruz, sc-24
validation reference: https://www.scbt.com/p/hsp-70-hsc-70-antibody-w27

HSP90 (C45G5), Cell Signaling, 4877
validation reference: https://www.cellsignal.com/products/primary-antibodies/hsp90-c45g5-rabbit-mab/4877

Ki67 (SP6), Abcam, ab16667
validation reference: https://www.abcam.com/ki67-antibody-sp6-ab16667.html

KIAA0753, Abcam, ab121736
validation reference: https://www.abcam.com/mnr-antibody-ab121736.html
WB/IF: KO in RPE-1 human cells; ED Fig 3a,b; siRNA in RPE-1 human cells; ED Fig 3h,i

MIB1, Sigma, M5948
validation reference: https://www.sigmaaldrich.com/CA/en/product/sigma/m5948
https://www.sigmaaldrich.com/certificates/Graphics/COfAInfo/sigmail01/pdf/M5948_SPEC.pdf

mono- and poly-ubiquitinylated conjugates (FK2), Enzo, BML-PW8810-0100
validation reference: https://www.epigentek.com/catalog/mono-and-polyubiquitinylated-conjugates-monoclonal-antibody-fk2-p-4653.html

NEDD1 (H-3), Santa Cruz, SC-398733
validation reference: https://www.scbt.com/p/nedd1-antibody-h-3

OFD1, Sigma-Atlas, HPA031103
validation reference: https://www.sigmaaldrich.com/CA/en/product/sigma/hpa031103
https://www.sigmaaldrich.com/CA/en/coa/SIGMA/HPA031103/A95451
WB/IF: KO in RPE-1 human cells; ED Fig 3e,f

p21 (F-5), Santa Cruz, sc-6246
validation reference: https://www.scbt.com/p/p21-antibody-f-5

p38α/β (A-12), Santa Cruz, sc-7972
validation reference: https://www.scbt.com/p/p38alpha-beta-antibody-a-12

p-p38 (E-1), Santa Cruz, sc-166182
validation reference: https://www.scbt.com/p/p-p38-antibody-e-1

p53 (DO-1), Santa Cruz, sc-126
validation reference: https://www.scbt.com/p/p53-antibody-do-1

p62 (SQSTM) (2C11), Abcam, ab56416
validation reference: https://www.abcam.com/sqstm1--p62-antibody-2c11-bsa-and-azide-free-ab56416.html

PCM1, Bethyl, A301-149A
validation reference: https://www.fortislife.com/products/primary-antibodies/rabbit-anti-pcm1-antibody/BETHYL-A301-149
WB: KO in RPE-1 human cells; ED Fig 3a

PCM1, Santa Cruz, sc-50164
validation reference: https://datasheets.scbt.com/sc-50164.pdf
IF: KO in RPE-1 human cells; ED Fig 3b

Pericentrin, Abcam, ab4448
validation reference: https://www.abcam.com/pericentrin-antibody-centrosome-marker-ab4448.html

PIBF1 (CEP90), Proteintech, 14413-1-AP
validation reference: https://www.ptglab.com/products/PIBF1-Antibody-14413-1-AP.htm
WB/IF: KO in RPE-1 human cells; ED Fig 3a,b; siRNA in RPE-1 human cells; ED Fig 3h,i

Anti-polyglutamylation modification (GT335), Adipogen, AG-20B-0020-C100
validation reference:

SAS6 (91.390.21), Santa Cruz, sc-81431

validation reference: https://www.scbt.com/p/sas-6-antibody-91-390-21

STIL, Abcam, ab89314
validation reference: https://www.abcam.com/stilsil-antibody-ab89314.html

Talpid3, Proteintech, 24421-1-AP
validation reference: https://www.ptglab.com/products/KIAA0586-Antibody-24421-1-AP.htm
WB/IF: siRNA in RPE-1 human cells; ED Fig. 5 c,d

USP33, Bethyl, A300-925A
validation reference: https://www.fortislife.com/products/primary-antibodies/rabbit-anti-usp33-antibody/BETHYL-A300-925
WB: siRNA in RPE-1 human cells; ED Fig. 5 b

Vimentin (V9), Sigma, V6389
validation reference: https://www.sigmaaldrich.com/CA/en/product/sigma/v6389
https://www.sigmaaldrich.com/specification-sheets/162/507/V6389-BULK________SIGMA____.pdf

Eukaryotic cell lines

Policy information about cell lines

Cell line source(s)	hTERT RPE-1 (female, human epithelial cells immortalized with hTERT), A-375 (female, human malignant melanoma epithelial), BJ-5ta (male, human fibroblasts immortalized with hTERT), HFF-1 (male, human primary fibroblasts), HeLa (female, human adenocarcinoma epithelial) , U-2 OS (female, human osteosarcoma epithelial), IMR90 (female, human diploid fibroblasts), and HEK293T(female, human kidney) cells were from ATCC.<br><br>hTERT RPE-1 cells stably expressing Cas9 were a gift from the Durocher lab (Nature. 2018 Jul; 559(7713): 285–289). The U-2 OS STIL CRISPR KO (Nat Commun. 2018. 9, 1731), hTERT RPE-1 PCM1 CRISPR KO (EMBO J. 2019 Jul 15; 38(14): e101109), hTERT RPE-1 FOP CRISPR KO (Open Biology. 2017 Jun; 7(6): 170114). have previously been published by the Pelletier lab. The hTERT RPE-1 CETN2 CRISPR KO (J. Cell Biol. 2015 Mar 16; 208(6): 693–701) was previously published by the Morrison lab. All other KO cell lines were generated in the course of this study, details of which are presented in the manuscript.
Authentication	None of the cell lines used were authenticated during the course of this study.
Mycoplasma contamination	Cell lines tested negative for mycoplasma contamination.
Commonly misidentified lines (See ICLAC register)	None of the cell lines used in this study are in the ICLAC registry.
