# Supplementary figures and images for: Aggresome assembly at the centrosome is driven by CP110–CEP97–CEP290 and centriolar satellites
Source: Nat Cell Biol. 2022 Apr 11;24(4):483–96. doi: 10.1038/s41556-022-00869-0 (PMC9033585; doi:10.1038/s41556-022-00869-0)

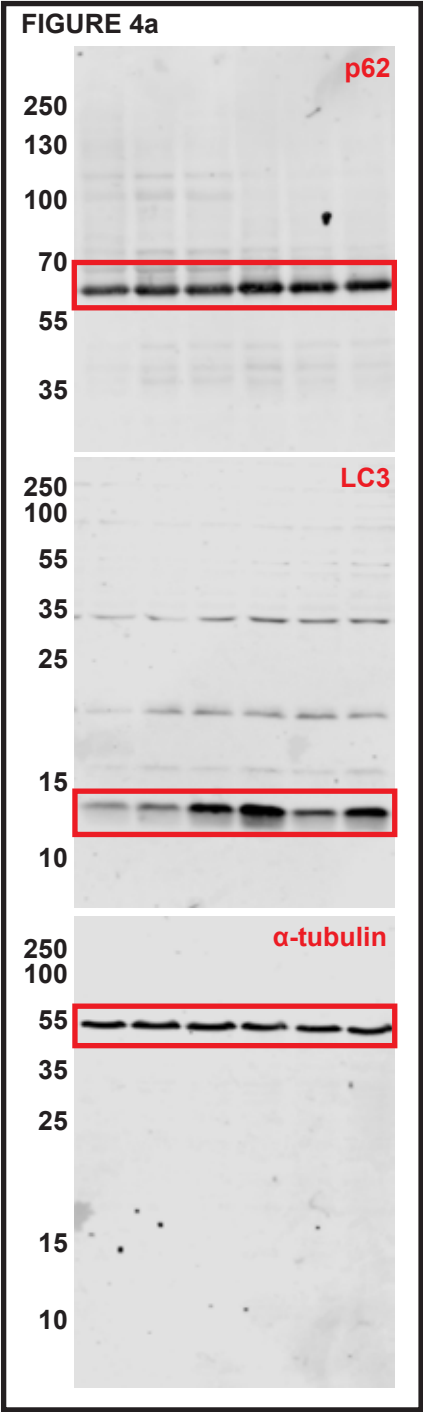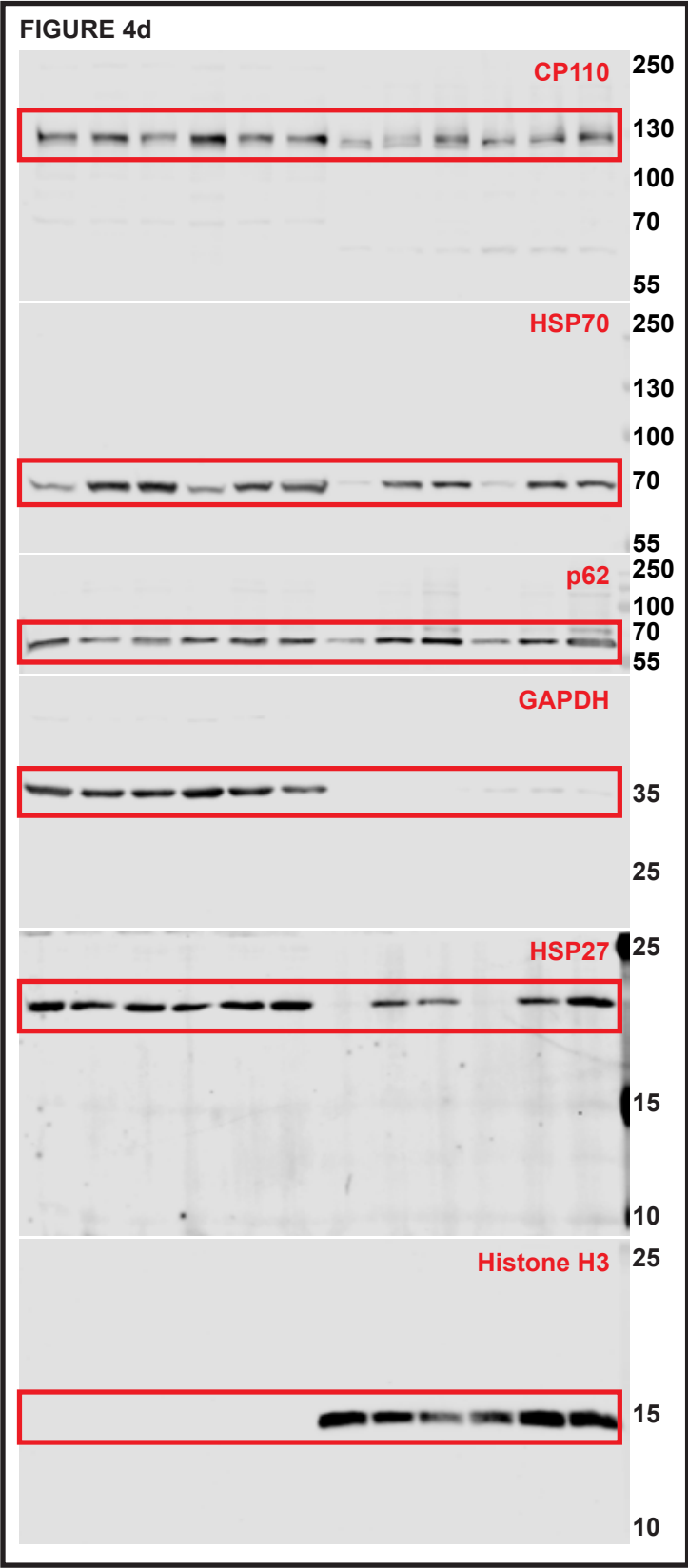

Supplement: Source Data Fig. 4 — Unprocessed western blots. [file 41556_2022_869_MOESM8_ESM.pdf]

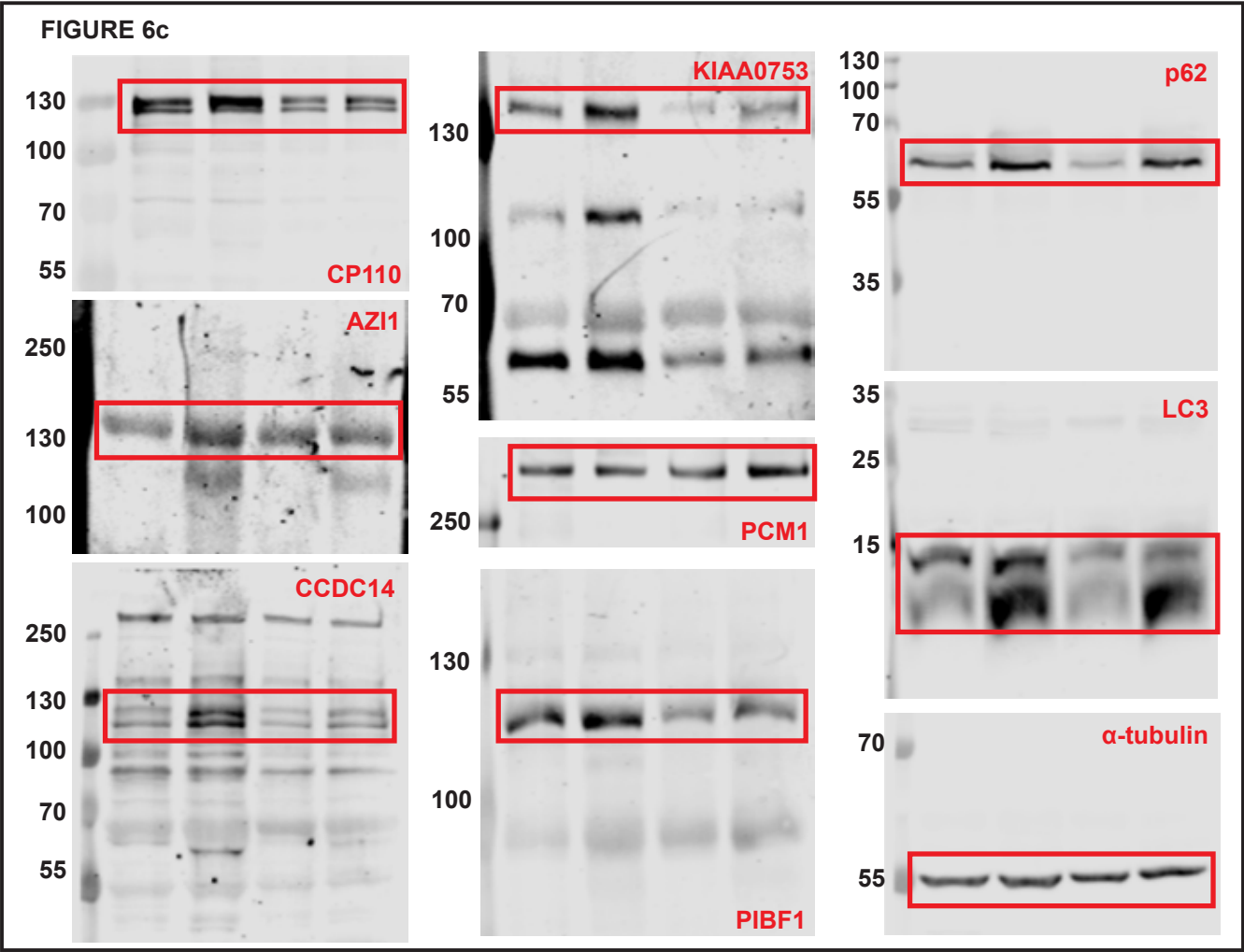

Supplement: Source Data Fig. 6 — Unprocessed western blots. [file 41556_2022_869_MOESM11_ESM.pdf]

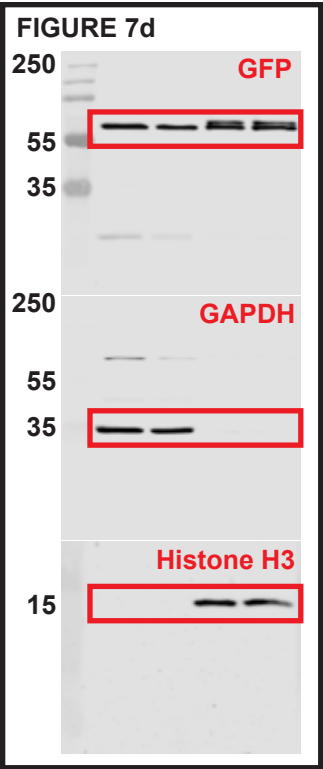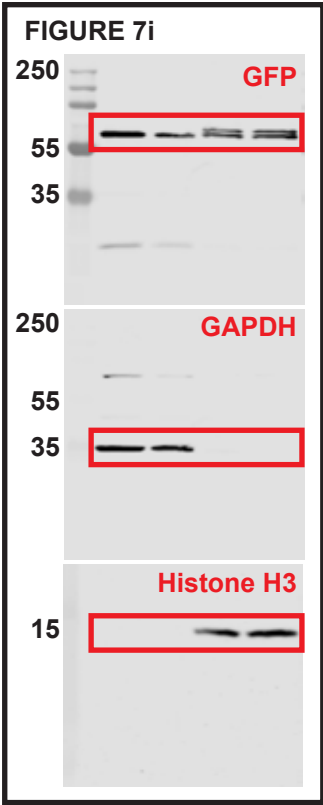

Supplement: Source Data Fig. 7 — Statistical source data. [file 41556_2022_869_MOESM12_ESM.pdf]

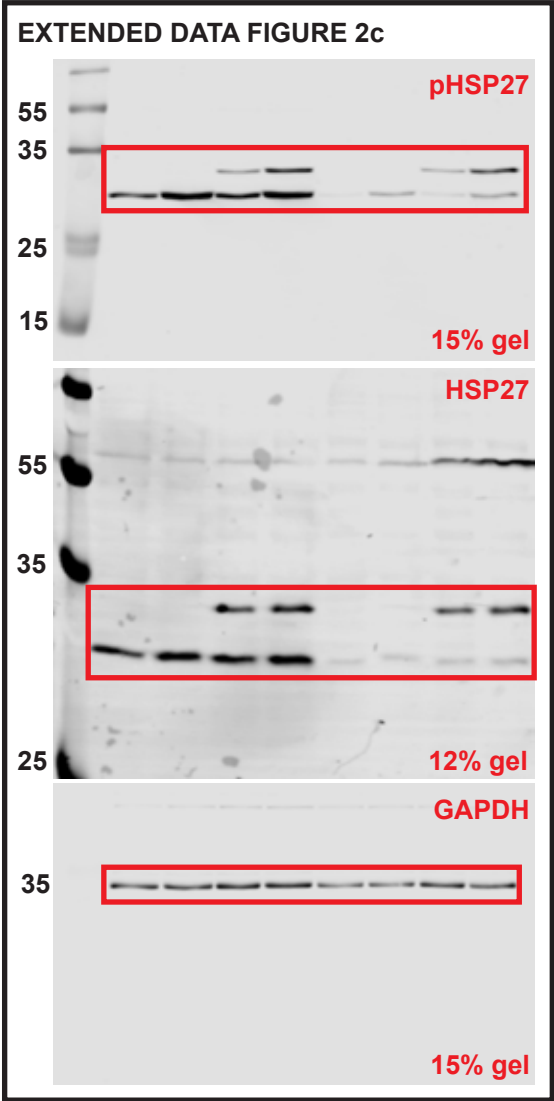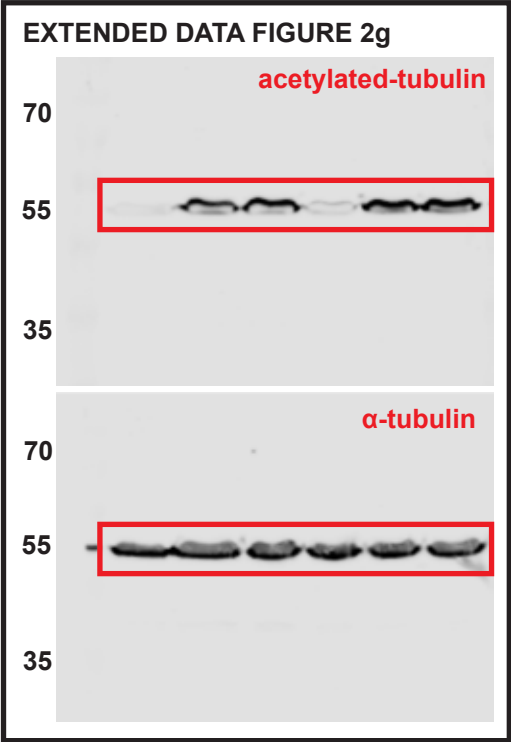

Supplement: Source Data Extended Data Fig. 2 — Unprocessed western blots. [file 41556_2022_869_MOESM16_ESM.pdf]

EXTENDED DATA FIGURE 3a

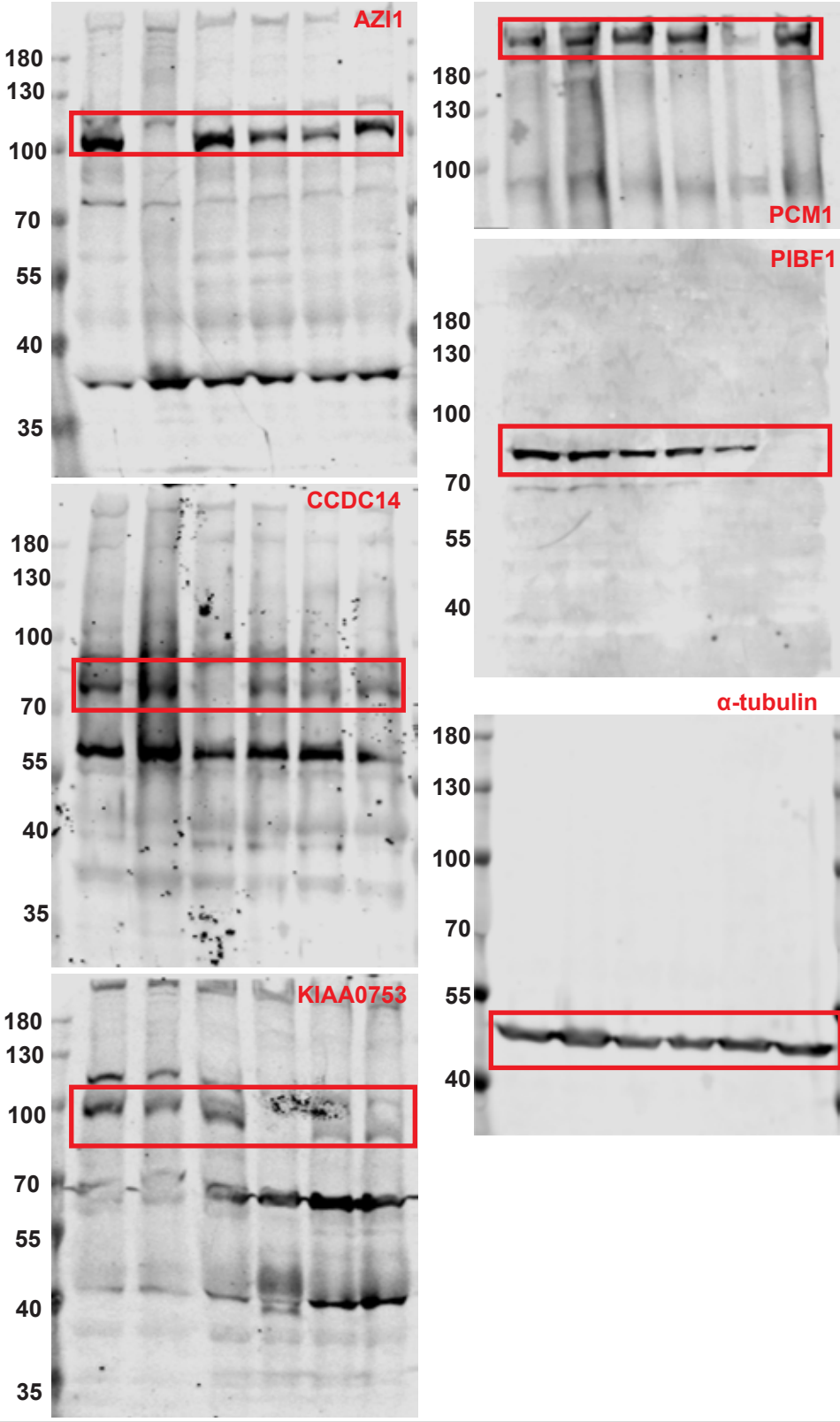

EXTENDED DATA FIGURE 3f

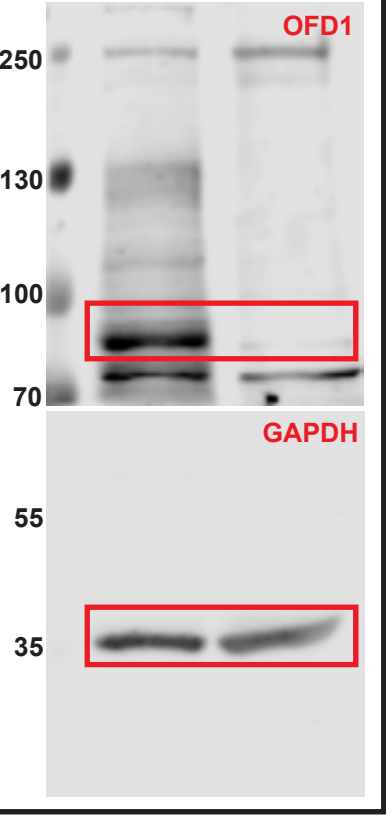

EXTENDED DATA FIGURE 3i

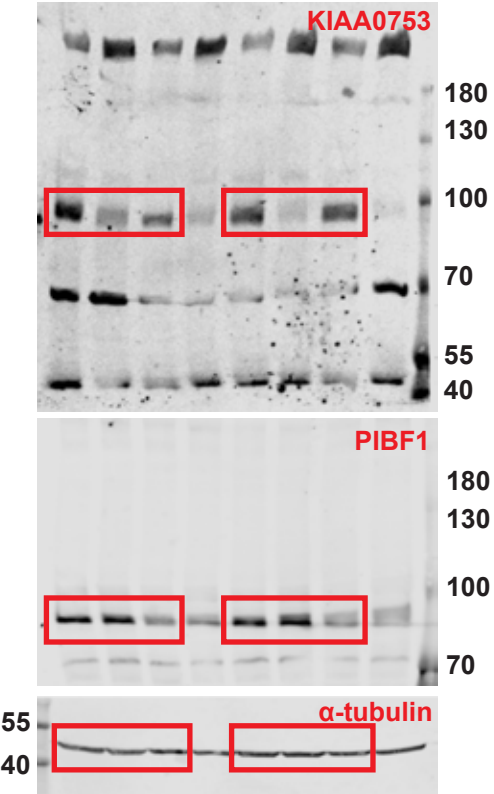

Supplement: Source Data Extended Data Fig. 3 — Unprocessed western blots. [file 41556_2022_869_MOESM18_ESM.pdf]

EXTENDED DATA FIGURE 4c

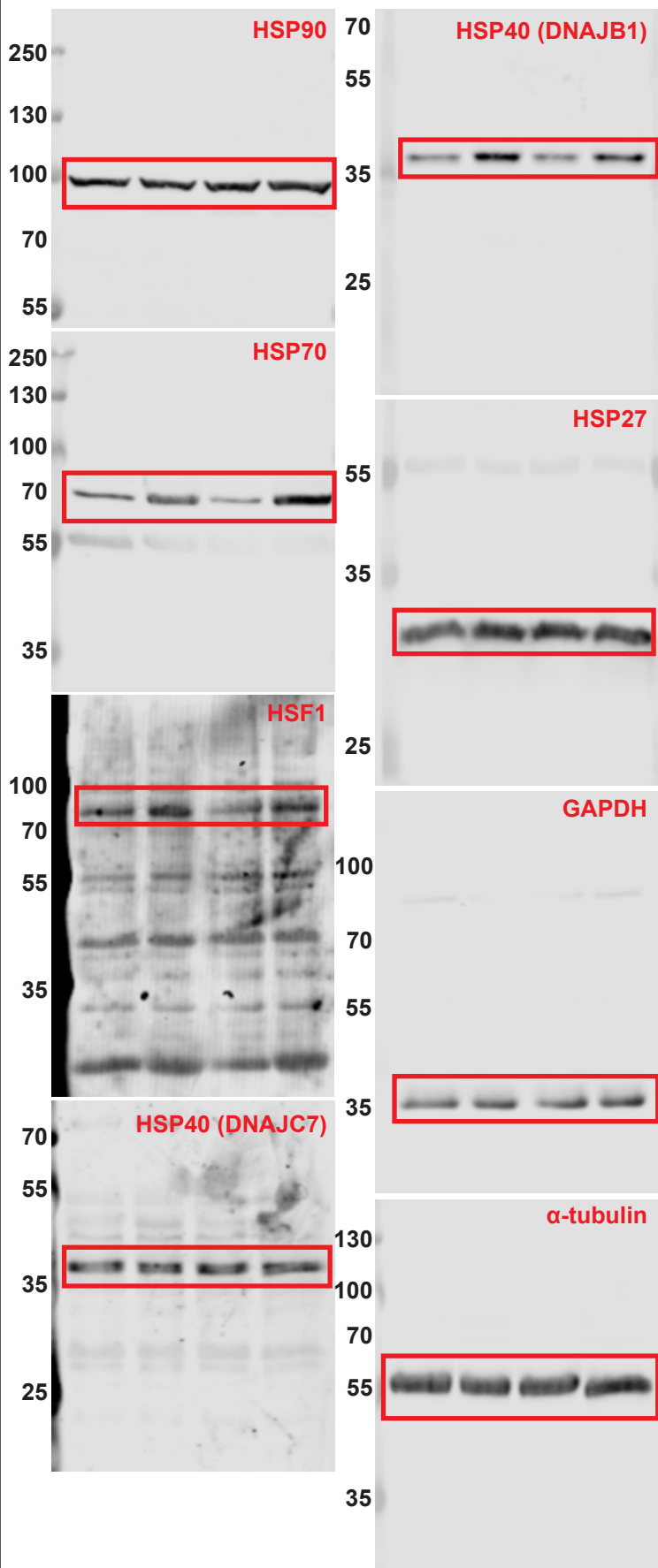

EXTENDED DATA FIGURE 4d

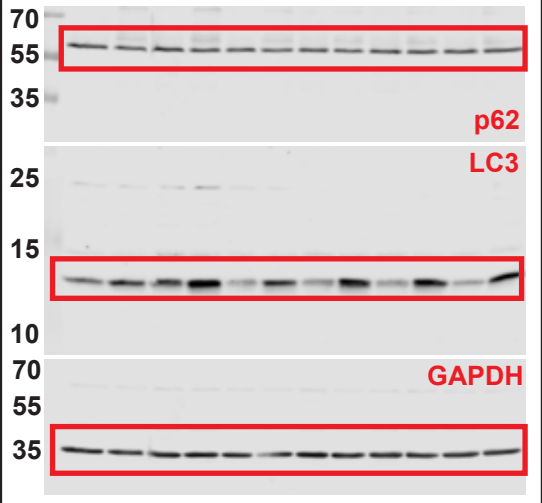

EXTENDED DATA FIGURE 4g

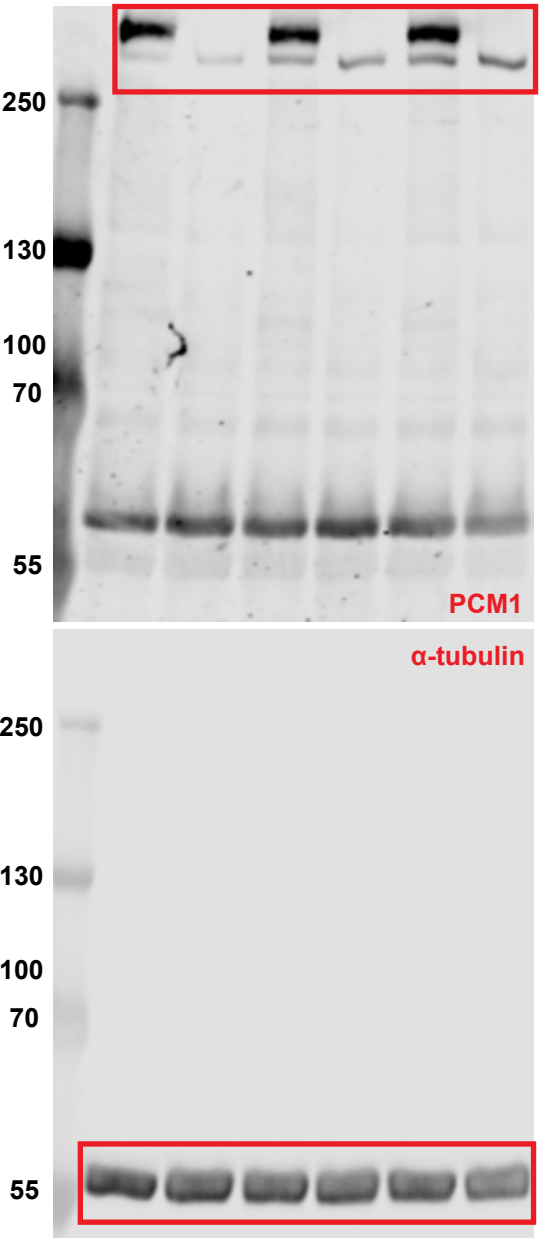

Supplement: Source Data Extended Data Fig. 4 — Unprocessed western blots. [file 41556_2022_869_MOESM20_ESM.pdf]

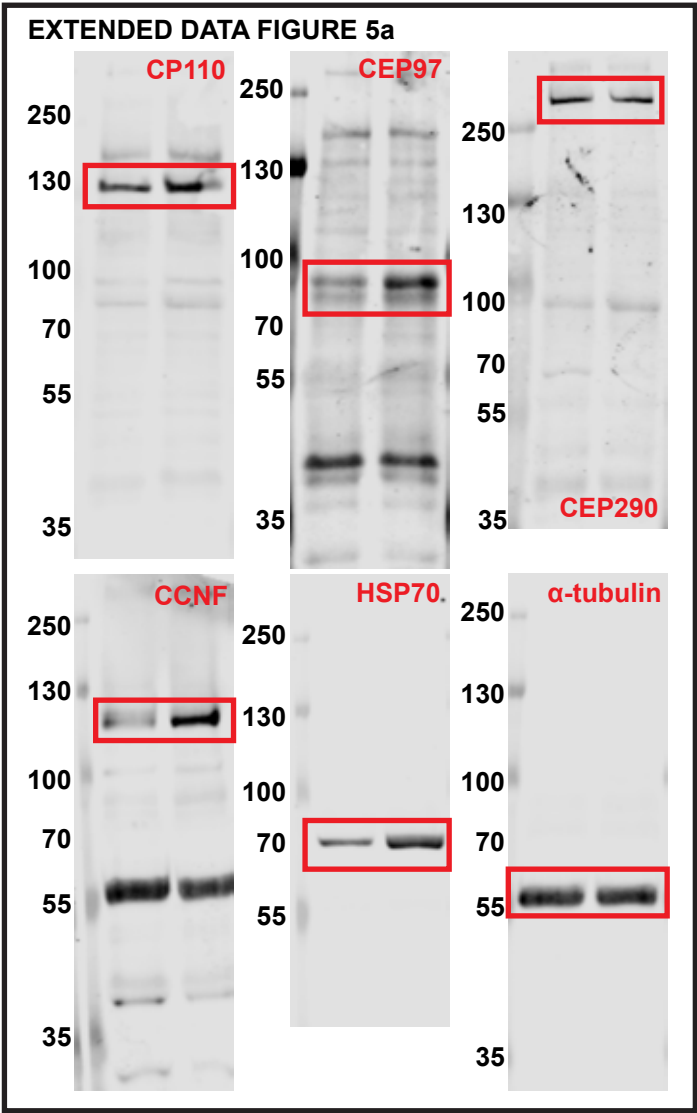

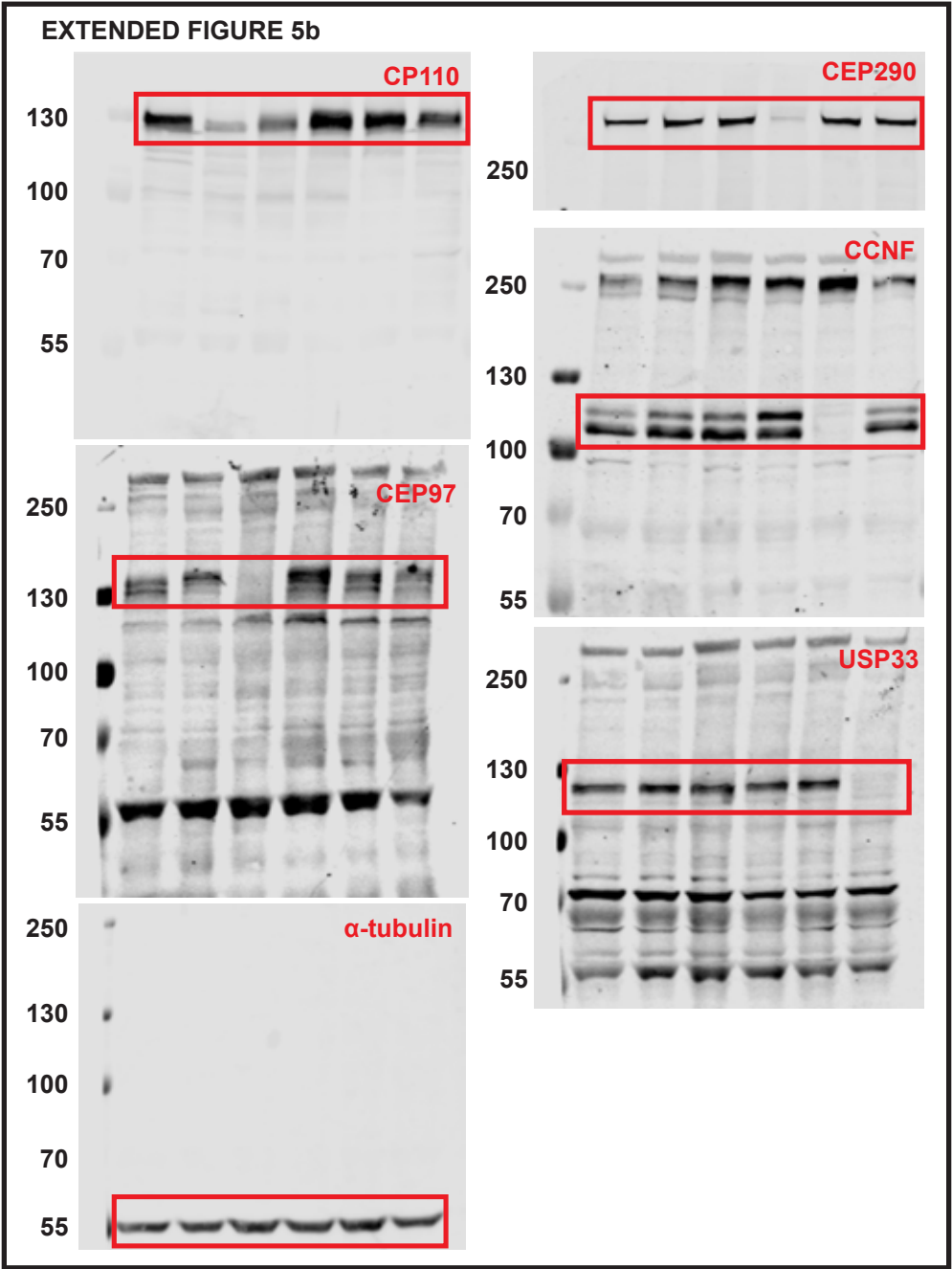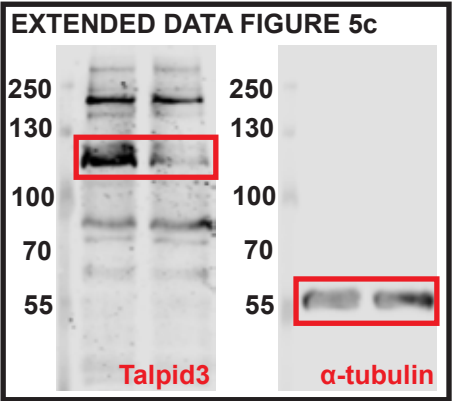

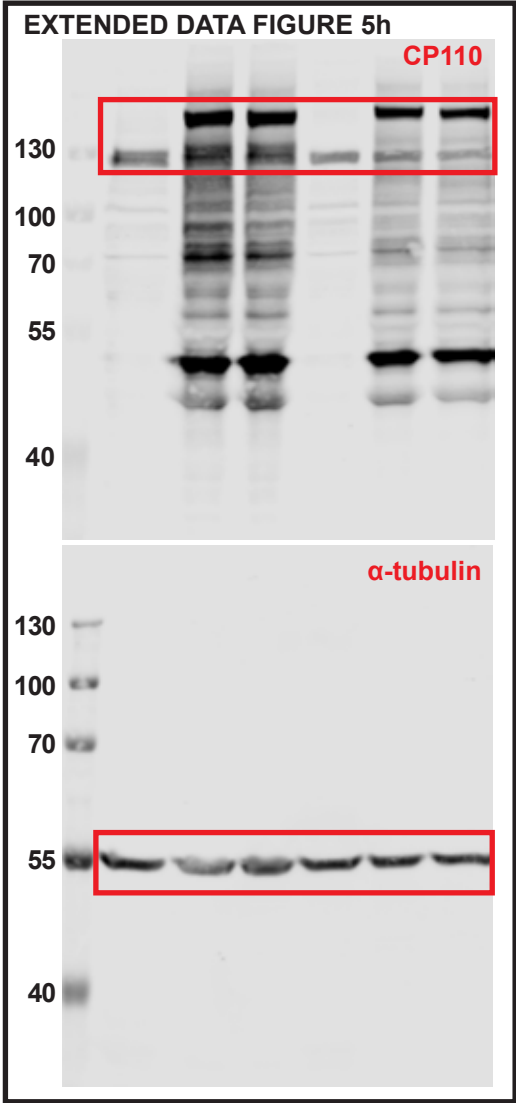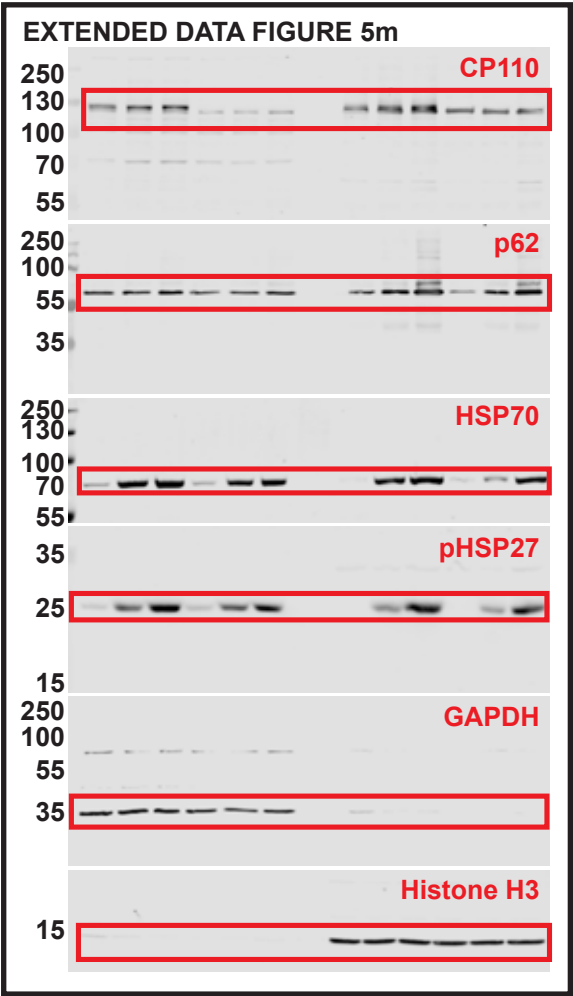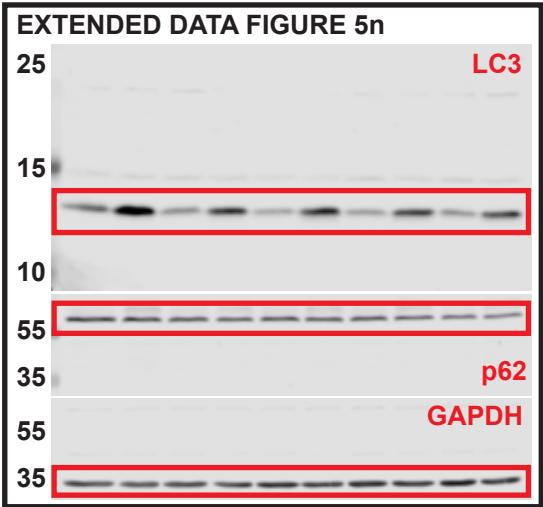

Supplement: Source Data Extended Data Fig. 5 — Unprocessed western blots. [file 41556_2022_869_MOESM22_ESM.pdf]

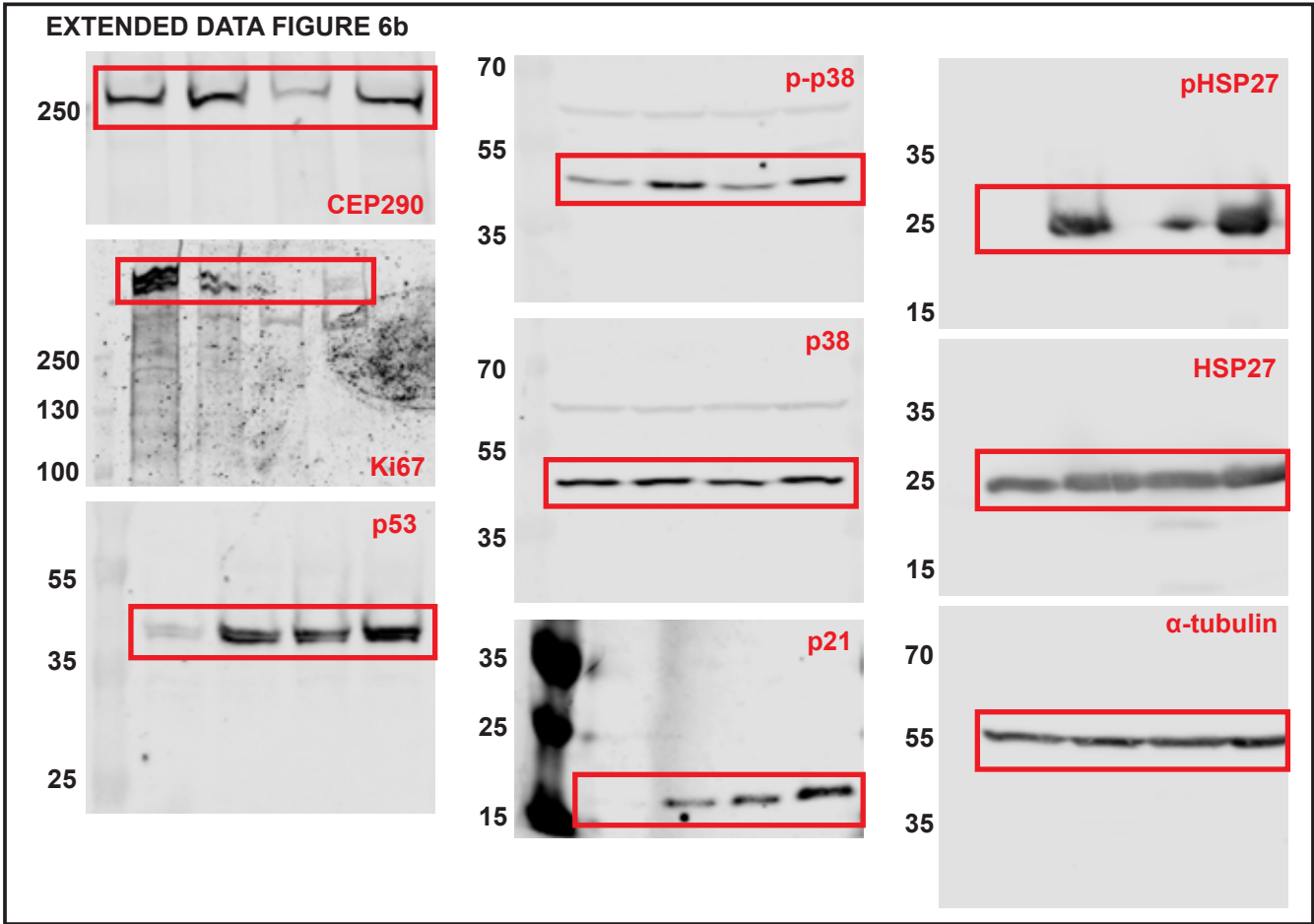

Supplement: Source Data Extended Data Fig. 6 — Unprocessed western blots. [file 41556_2022_869_MOESM24_ESM.pdf]

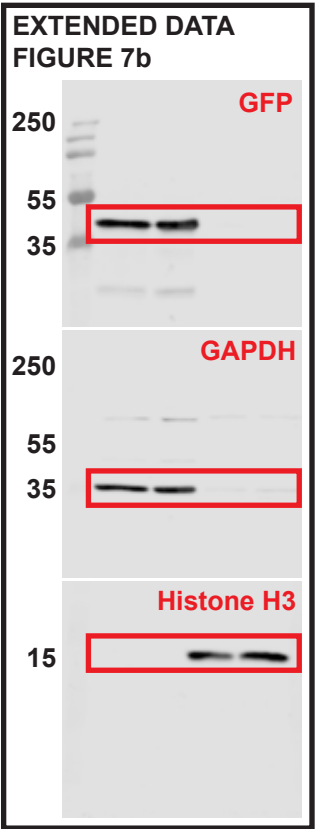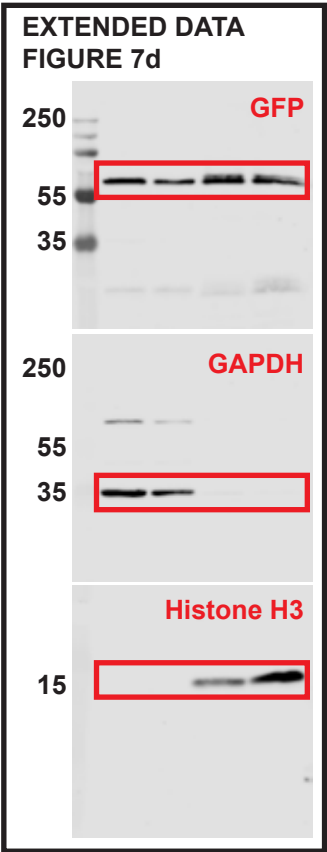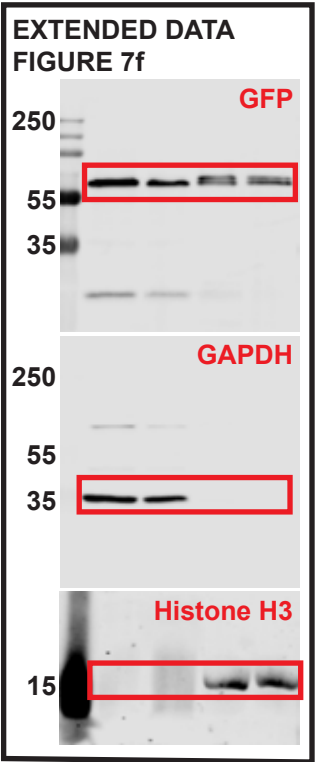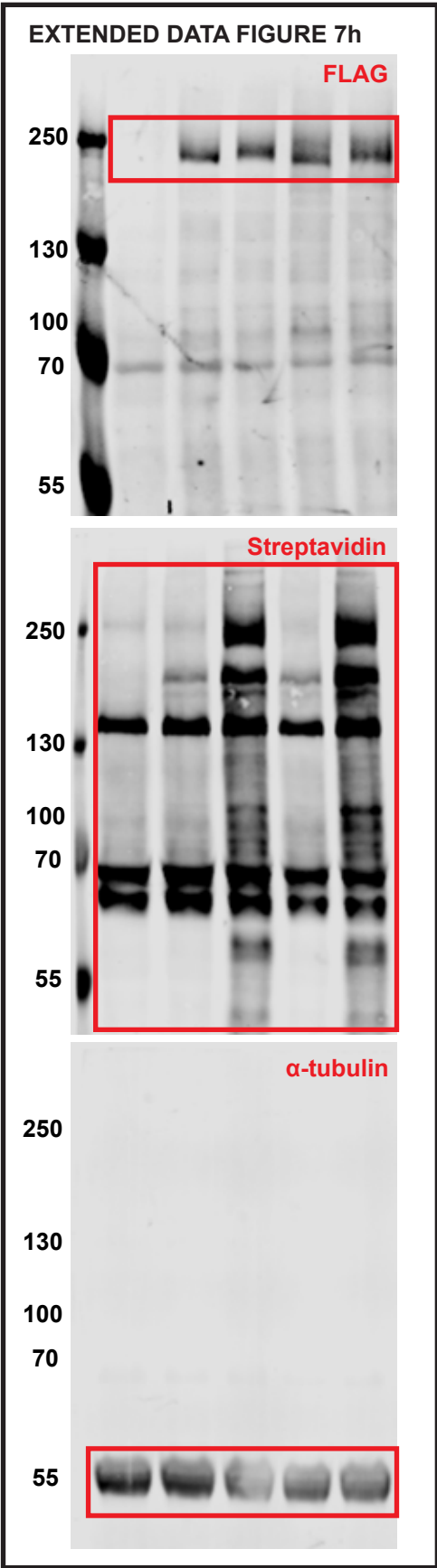

Supplement: Source Data Extended Data Fig. 7 — Unprocessed western blots. [file 41556_2022_869_MOESM25_ESM.pdf]
