# Supplementary material for: Aggresome assembly at the centrosome is driven by CP110–CEP97–CEP290 and centriolar satellites
Source: Nat Cell Biol. 2022 Apr 11;24(4):483–96. doi: 10.1038/s41556-022-00869-0 (PMC9033585; doi:10.1038/s41556-022-00869-0)
Supplement: Supplementary file 2 — Peer Review Information [file 41556_2022_869_MOESM2_ESM.pdf]

natureresearch

# Peer Review Information

**Journal:** Nature Cell Biology
**Manuscript Title:** Aggresome assembly at the centrosome is driven by CP110-CEP97-CEP290 and centriolar satellites
**Corresponding author name(s):** Laurence Pelletier

## Reviewer Comments & Decisions:

### Decision Letter, initial version:

Dear Dr Pelletier,

Your manuscript, "The CP110-CEP97-CEP290 module orchestrates a centriolar satellite dependent response to proteotoxic stress", has now been seen by 3 referees, who are experts in centrosomes (referee 1); aggresome (referee 2); and proteotoxic stress (referee 3). As you will see from their comments (attached below) they find this work of potential interest, but have raised substantial concerns, which in our view would need to be addressed with considerable revisions before we can consider publication in Nature Cell Biology.

Nature Cell Biology editors discuss the referee reports in detail within the editorial team, including the chief editor, to identify key referee points that should be addressed with priority, and requests that are overruled as being beyond the scope of the current study. To guide the scope of the revisions, I have listed these points below. We are committed to providing a fair and constructive peer-review process, so please feel free to contact me if you would like to discuss any of the referee comments further.

In particular, it would be essential to:

A) Demonstrate the specificity of satellite components in aggresome formation (reviewer #1)

B) Demonstrate the physiological relevance of aggresome formation (reviewer #1)

C) Better explain why CEP290/CP110/CEP97 module is responsible for aggresome assembly (reviewer #1)

D) Develop the mechanistic insight into aggresome formation (reviewer #2)

E) Improve the method to define aggresomes (reviewer #2)

F) All other referee concerns pertaining to strengthening existing data, providing controls, methodological details, clarifications and textual changes, should also be addressed.

G) Finally please pay close attention to our guidelines on statistical and methodological reporting (listed below) as failure to do so may delay the reconsideration of the revised manuscript. In particular please provide:

- a Supplementary Figure including unprocessed images of all gels/blots in the form of a multi-page pdf file. Please ensure that blots/gels are labeled and the sections presented in the figures are clearly indicated.

- a Supplementary Table including all numerical source data in Excel format, with data for different figures provided as different sheets within a single Excel file. The file should include source data giving rise to graphical representations and statistical descriptions in the paper and for all instances where the figures present representative experiments of multiple independent repeats, the source data of all repeats should be provided.

We would be happy to consider a revised manuscript that would satisfactorily address these points, unless a similar paper is published elsewhere, or is accepted for publication in Nature Cell Biology in the meantime.

When revising the manuscript please:

- ensure that it conforms to our format instructions and publication policies (see below and https://www.nature.com/nature/for-authors).

- provide a point-by-point rebuttal to the full referee reports verbatim, as provided at the end of this letter.

- provide the completed Reporting Summary (found here https://www.nature.com/documents/nr-reporting-summary.pdf). This is essential for reconsideration of the manuscript will be available to editors and referees in the event of peer review. For more information see http://www.nature.com/authors/policies/availability.html or contact me.

When submitting the revised version of your manuscript, please pay close attention to our href="https://www.nature.com/nature-research/editorial-policies/image-integrity">Digital Image Integrity Guidelines. and to the following points below:

-- that unprocessed scans are clearly labelled and match the gels and western blots presented in figures.
-- that control panels for gels and western blots are appropriately described as loading on sample processing controls
-- all images in the paper are checked for duplication of panels and for splicing of gel lanes.

Finally, please ensure that you retain unprocessed data and metadata files after publication, ideally

archiving data in perpetuity, as these may be requested during the peer review and production process or after publication if any issues arise.

Nature Cell Biology is committed to improving transparency in authorship. As part of our efforts in this direction, we are now requesting that all authors identified as ‘corresponding author’ on published papers create and link their Open Researcher and Contributor Identifier (ORCID) with their account on the Manuscript Tracking System (MTS), prior to acceptance. ORCID helps the scientific community achieve unambiguous attribution of all scholarly contributions. You can create and link your ORCID from the home page of the MTS by clicking on ‘Modify my Springer Nature account’. For more information please visit please visit www.springernature.com/orcid.

This journal strongly supports public availability of data. Please place the data used in your paper into a public data repository, or alternatively, present the data as Supplementary Information. If data can only be shared on request, please explain why in your Data Availability Statement, and also in the correspondence with your editor. Please note that for some data types, deposition in a public repository is mandatory - more information on our data deposition policies and available repositories appears below.

Please submit the revised manuscript files and the point-by-point rebuttal to the referee comments using this link:

[REDACTED]

*This url links to your confidential home page and associated information about manuscripts you may have submitted or be reviewing for us. If you wish to forward this email to co-authors, please delete the link to your homepage.

We would like to receive a revised submission within six months.

We hope that you will find our referees' comments, and editorial guidance helpful. Please do not hesitate to contact me if there is anything you would like to discuss.

Best wishes,

Ana Mateus

Ana Mateus
Editor
Nature Cell Biology

Reviewers' Comments:

Reviewer #1:
Remarks to the Author:

The manuscript by Prosser et al describes a new molecular pathway that facilitate aggresome formation in response to proteotoxic stress. This pathway utilises the centrosomal components CP110, CEP290 and CEP97 to seed the aggresome, which then expands via recruitment of centriolar satellites. Furthermore, the authors find that CP110 is rate-limiting for aggresome formation in senescent cells and that HTT-PolyQ inclusions also require the CP110-CEP97-CEP290 and PCM1-containing satellites. Altogether the results reported here expose a vital contribution of centrosomes and centriolar satellites to aggresome assembly.

The manuscript is written in a clear and concise manner with data quality and presentation being excellent. I anticipate the topic to be of interest to the wider cell biology community encompassing multiple fields and is thus well-suited for Nature Cell Biology.

Specific points:

1. I find the link between centriolar satellites and aggresome assembly compelling. However, it seems surprising that knockouts of PCM1, AZI1, CCDC14, PIBF1/CEP90, all impact aggresome formation to the same degree, despite affecting satellite formation and distribution in very different ways. Therefore, it would be important to identify one or two satellite components that do not influence aggresome formation.

2. What is the consequence of cells not being able to assemble larger aggresomes in the satellite knockout cell lines? Is there any data from such cell lines that baseline proteotoxic stress is elevated? Do PCM1 null cells exhibit signs of senescence or limited proliferative capacity?

3. Aggresome size is a function of its assembly and clearance. Could the authors show a time course as to how these aggresomes behave over time in cells that lack the CEP290/CP110 module or satellites? For instance, do they grow larger if MG132 treatment is continued beyond 5 hr, or is the size shown the limit? This does not need to be live, it would be interesting to see average sizes across the population at different timepoints.

4. The data on GFP-CP110 overexpression in senescent cells (Fig 6f) is certainly interesting but the conclusion that restoration of CP110 levels in senescence is sufficient for MG132-induced aggresome formation is not fully supported by the results. GFP-CP110 overexpression indeed leads to accumulation of pHSP27 around the centrosomal region. However, the scattered granular cytoplasmic GFP-CP110 signal largely co-localises with pHSP27 in absence of MG132, and therefore I wonder if what we see are GFP-CP110 aggregates that associate with pHSP27, rather than true aggresomes. This caveat should be mentioned. A similar co-localisation between pHSP27 with cytoplasmic CEP290 aggregates is also seen with FLAG-CEP290 overexpression in Sup Fig 5. Because many centrosomal proteins form large cytoplasmic aggregates/polymers when overexpressed the authors should be careful to distinguish these from aggresomes. Using additional aggresome markers could be useful in this regard. In addition, expression of many proteins is reduced in senescence and so a reduction in CP110 and CEP290 levels is not entirely unexpected. A more thorough analysis of centriolar/PCM/satellite protein levels would be necessary to support the hypothesis that CP110 levels are limiting in senescence for aggresome formation.

5. The authors should elaborate further in the discussion on why they think the CEP290/CP110/CEP97 module is responsible for aggresome assembly. Is it because they are still transported to centrosomes in a misfolded state, possibly in a complex? Do GFP-CP110 and FLAG-CEP290 aggregates contain

CEP290, CP110 and CEP97? Have the authors investigated contribution by Talpid3, which also interacts with CEP290? A western blot that shows changes in levels of these components after MG132 treatment would be useful to include. Perhaps, this complex drives aggresome assembly because stoichiometry of its components is particularly sensitive to MG132 treatment. It would be useful to include any centrosome protein knockout that does not impact on aggresome formation.

6. The paper contains a lot of new information and would benefit from a more detailed discussion.

7. Line 319: Thereby, assigning…
This seems to be an incomplete sentence.

Reviewer #2:
Remarks to the Author:
In this manuscript, Prosser et al. provide evidence that centriolar components are required to form the perinuclear inclusion, aggresomes. By employing higher resolution microscopy, it was shown that selective centriolar and pericentriolar (PCM) proteins are expanded from typical two centriolar spots into multiple foci within the areas occupied by aggresomes (marked by pHsp27) induced by a proteasome inhibitor, MG132. Systematic knockdown of known components of centrioles and centriolar satellite showed that selective centriolar proteins, including PCM-1 as well theCP110-CEP97-CEP290 module, are required for MG132-induced aggresome and mutant Htt103Q inclusion formation. In the last two decades, significant progress has been made on the origin of protein aggregates that constitute aggresomes and the machinery involved in the dynamic concentration and clearance of aggresome at the MTOC. The identification of centriolar satellite components as a new component involved in aggresome formation adds another level of understanding to cellular proteostasis. However, there are two main issues with the current report. First, although genetic knockdown of centrosome satellite components can affect the formation or size of aggresomes, there is no clear mechanistic picture of how these factors might affect the production, transport, or processing of protein aggregates associated with aggresome biogenesis. Second, the definition of aggresomes is some analysis would need more detailed analysis. For example, the conclusion that cyclin F (CCNF) inactivation leads to spontaneous aggresome formation requires more careful analysis. The appearance of Ub+ foci or CETN2 foci (> 4 in numbers) was interpreted as spontaneous aggresome formation upon CCNF knockdown. However, it is unclear whether this conclusion is accurate, as it is difficult to judge what the CETN2 foci are and whether they genuinely reflect a significant accumulation of protein aggregates. As aggresomes are generally thought to arise from peripheral aggregates tagged by p62, the identity of CETN2 foci needs additional protein aggregate/inclusion markers, such as p62. The definition of aggresomes is a potential issue throughout the report (See points 2 and 3 for details).
In conclusion, although the identification of centriolar machinery is in aggresome formation is novel, the lack of significant mechanistic insight and the unconventional method to define aggresomes makes it difficult to judge the key findings' importance or relevance. With these significant deficiencies, this report, in its current form, is not suitable for Nature Cell Biology.

Other comments:
1. The relationship between the "centriolar" foci and aggresome (protein aggregates) needs to be better defined. For example, in Fig 1, the relationship between satellite markers and pHsp70 is challenging to grasp from one representative image. Are satellite foci and pHsp27 foci always over-

lapping with each other? A more quantitative co-localization analysis would be helpful.
2. The size of aggresomes in this report appears to be small compared with previous reports. It was noted that aggresomes were induced by proteasome inhibitor treatment for 5 hrs. This is a relatively short treatment to induce aggresomes for standard imaging and biochemical analysis (typical treatment time is ~ 24 hrs). Because of the concern raised in point 1, it would be reassuring to induce large aggresomes by longer inhibitor treatment and then investigate the distribution of representative PCM and satellite markers and their knockdown effect.
3. Fig 6E and F have a similar issue of defining aggresomes in senescent cells transfected with GFP-CP110. The size of the aggresome is very small in both DMSO and MG132 treated transfected cells. Therefore, it is very difficult to conclude that GFP-CP110 can promote aggresome formation in senescent cells. Because transfected and overexpressed proteins could sometimes become aggregated upon MG132 treatment, a proper GFP-CP110 mutant control should be included to guard against this potential pitfall.
4. Almost all images presented in the study only show a small cell region corresponding to the MTOC, where aggresomes typically form. This presentation might lose some vital information as protein aggregates accumulate at the cell periphery before their concentration. It would be informative to present images covering the entire cells in key figures, particularly in figures where inclusion formation was suppressed by PCM1 or CP110/CEP97/CEP290 knockdown.
5. The effects of PCM-1 and CP110/CEP97/CEP290 knockdown on mutant HTT inclusion formation is interesting (fig 6h), but proper evaluation of the expression levels of GFP-HTT in both soluble and insoluble fractions is needed. The inclusion of independent biochemical analyses on protein aggregate processing would strengthen the conclusions deduced from image analyses.
6. The effect of Hsp27 knockdown on suppressing aggresome formation has not been described previously (Supplementary fig 2). To ensure the effect of knockdown is specific, a genetic rescue experiment with Hsp27 expression plasmid is recommended.

Reviewer #3:
Remarks to the Author:
In the manuscript Prosser an colleagues investigates mechanisms of aggresome formation. They use high resolution microscopy to understand how components of centrosome affect aggresome formation. They demonstrate that components of the satellites are critical for the process. Furthermore, they identified components of the centrosome that control aggresome formation in response to proteasome inhibitors. Finally the demonstrate that downregulation of these components in senescence is responsible for poor induction of aggresome under these conditions. Overall the work is very original and novel. The quality of data is superb, and appropriate statistics has been provided.
Overall the story is very interesting and significantly advances the field.
There are few minor problems that I would like to comment on.
1. Light yellow schemes as in Fig. 2D are difficult to see.
2. It seems that HDAC6 had relatively minor effect on aggresome. This point needs to be highlighted.
3. When talking about effects of CCNF, it would be important to show a cell with aggresome at lower resolution. Otherwise it is not clear if depletion of CCNF indeed cause aggresome formation in the absence of proteasome inhibitors or just increases the number of foci.
4. There is a controversy about the relevance of Htt inclusion body and aggresome. It would be important to test if microtubular transport is necessary for the process or centriole just forms a nucelation site for Htt and soluble molecules join the inclusion body by simple diffusion.
References provide appropriate credit to previous work.

The paper is very well written.

GUIDELINES FOR MANUSCRIPT SUBMISSION TO NATURE CELL BIOLOGY

READABILITY OF MANUSCRIPTS – Nature Cell Biology is read by cell biologists from diverse backgrounds, many of whom are not native English speakers. Authors should aim to communicate their findings clearly, explaining technical jargon that might be unfamiliar to non-specialists, and avoiding non-standard abbreviations. Titles and abstracts should concisely communicate the main findings of the study, and the background, rationale, results and conclusions should be clearly explained in the manuscript in a manner accessible to a broad cell biology audience. Nature Cell Biology uses British spelling.

MANUSCRIPT FORMAT – please follow the guidelines listed in our Guide to Authors regarding manuscript formats at Nature Cell Biology.

TITLE – should be no more than 100 characters including spaces, without punctuation and avoiding technical terms, abbreviations, and active verbs..

AUTHOR NAMES – should be given in full.

AUTHOR AFFILIATIONS – should be denoted with numerical superscripts (not symbols) preceding the names. Full addresses should be included, with US states in full and providing zip/post codes. The corresponding author is denoted by: "Correspondence should be addressed to [initials]."

ABSTRACT AND MAIN TEXT – please follow the guidelines that are specific to the format of your manuscript, as listed in our Guide to Authors (http://www.nature.com/ncb/pdf/ncb_gta.pdf) Briefly, Nature Cell Biology Articles, Resources and Technical Reports have 3500 words, including a 150 word abstract, and the main text is subdivided in Introduction, Results, and Discussion sections. Nature Cell Biology Letters have up to 2500 words, including a 180 word introductory paragraph (abstract), and the text is not subdivided in sections.

ACKNOWLEDGEMENTS – should be kept brief. Professional titles and affiliations are unnecessary. Grant numbers can be listed.

AUTHOR CONTRIBUTIONS – must be included after the Acknowledgements, detailing the contributions of each author to the paper (e.g. experimental work, project planning, data analysis etc.). Each author should be listed by his/her initials.

FINANCIAL AND NON-FINANCIAL COMPETING INTERESTS – the authors must include one of three

declarations: (1) that they have no financial and non-financial competing interests; (2) that they have financial and non-financial competing interests; or (3) that they decline to respond, after the Author Contributions section. This statement will be published with the article, and in cases where financial and non-financial competing interests are declared, these will be itemized in a web supplement to the article. For further details please see https://www.nature.com/licenceforms/nrg/competing-interests.pdf.

REFERENCES – are limited to a total of 70 for Articles, Resources, Technical Reports; and 40 for Letters. This includes references in the main text and Methods combined. References must be numbered sequentially as they appear in the main text, tables and figure legends and Methods and must follow the precise style of Nature Cell Biology references. References only cited in the Methods should be numbered consecutively following the last reference cited in the main text. References only associated with Supplementary Information (e.g. in supplementary legends) do not count toward the total reference limit and do not need to be cited in numerical continuity with references in the main text. Only published papers can be cited, and each publication cited should be included in the numbered reference list, which should include the manuscript titles. Footnotes are not permitted.

METHODS – Nature Cell Biology publishes methods online. The methods section should be provided as a separate Word document, which will be copyedited and appended to the manuscript PDF, and incorporated within the HTML format of the paper.

Methods should be written concisely, but should contain all elements necessary to allow interpretation and replication of the results. As a guideline, Methods sections typically do not exceed 3,000 words. The Methods should be divided into subsections listing reagents and techniques. When citing previous methods, accurate references should be provided and any alterations should be noted. Information must be provided about: antibody dilutions, company names, catalogue numbers and clone numbers for monoclonal antibodies; sequences of RNAi and cDNA probes/primers or company names and catalogue numbers if reagents are commercial; cell line names, sources and information on cell line identity and authentication. Animal studies and experiments involving human subjects must be reported in detail, identifying the committees approving the protocols. For studies involving human subjects/samples, a statement must be included confirming that informed consent was obtained. Statistical analyses and information on the reproducibility of experimental results should be provided in a section titled "Statistics and Reproducibility".

All Nature Cell Biology manuscripts submitted on or after March 21 2016 must include a Data availability statement as a separate section after Methods but before references, under the heading "Data Availability". . For Springer Nature policies on data availability see http://www.nature.com/authors/policies/availability.html; for more information on this particular policy see http://www.nature.com/authors/policies/data/data-availability-statements-data-citations.pdf. The Data availability statement should include:

• Accession codes for primary datasets (generated during the study under consideration and designated as "primary accessions") and secondary datasets (published datasets reanalysed during the study under consideration, designated as "referenced accessions"). For primary accessions data should be made public to coincide with publication of the manuscript. A list of data types for which submission to community-endorsed public repositories is mandated (including sequence, structure, microarray, deep sequencing data) can be found here http://www.nature.com/authors/policies/availability.html#data.

• Unique identifiers (accession codes, DOIs or other unique persistent identifier) and hyperlinks for datasets deposited in an approved repository, but for which data deposition is not mandated (see here for details http://www.nature.com/sdata/data-policies/repositories).

• At a minimum, please include a statement confirming that all relevant data are available from the authors, and/or are included with the manuscript (e.g. as source data or supplementary information), listing which data are included (e.g. by figure panels and data types) and mentioning any restrictions on availability.

• If a dataset has a Digital Object Identifier (DOI) as its unique identifier, we strongly encourage including this in the Reference list and citing the dataset in the Methods.

We recommend that you upload the step-by-step protocols used in this manuscript to the Protocol Exchange. More details can found at www.nature.com/protocolexchange/about.

DISPLAY ITEMS – main display items are limited to 6-8 main figures and/or main tables for Articles, Resources, Technical Reports; and 5 main figures and/or main tables for Letters. For Supplementary Information see below.

FIGURES – Colour figure publication costs $600 for the first, and $300 for each subsequent colour figure. All panels of a multi-panel figure must be logically connected and arranged as they would appear in the final version. Unnecessary figures and figure panels should be avoided (e.g. data presented in small tables could be stated briefly in the text instead).

All imaging data should be accompanied by scale bars, which should be defined in the legend. Cropped images of gels/blots are acceptable, but need to be accompanied by size markers, and to retain visible background signal within the linear range (i.e. should not be saturated). The boundaries of panels with low background have to be demarked with black lines. Splicing of panels should only be considered if unavoidable, and must be clearly marked on the figure, and noted in the legend with a statement on whether the samples were obtained and processed simultaneously. Quantitative comparisons between samples on different gels/blots are discouraged; if this is unavoidable, it should only be performed for samples derived from the same experiment with gels/blots were processed in parallel, which needs to be stated in the legend.

Figures should be provided at approximately the size that they are to be printed at (single column is 86 mm, double column is 170 mm) and should not exceed an A4 page (8.5 x 11"). Reduction to the scale that will be used on the page is not necessary, but multi-panel figures should be sized so that the whole figure can be reduced by the same amount at the smallest size at which essential details in each panel are visible. In the interest of our colour-blind readers we ask that you avoid using red and green for contrast in figures. Replacing red with magenta and green with turquoise are two possible colour-safe alternatives. Lines with widths of less than 1 point should be avoided. Sans serif typefaces, such as Helvetica (preferred) or Arial should be used. All text that forms part of a figure should be rewritable and removable.

We accept files from the following graphics packages in either PC or Macintosh format:

- For line art, graphs, charts and schematics we prefer Adobe Illustrator (.AI), Encapsulated PostScript (.EPS) or Portable Document Format (.PDF). Files should be saved or exported as such directly from the application in which they were made, to allow us to restyle them according to our journal house style.

- We accept PowerPoint (.PPT) files if they are fully editable. However, please refrain from adding PowerPoint graphical effects to objects, as this results in them outputting poor quality raster art. Text used for PowerPoint figures should be Helvetica (preferred) or Arial.

- We do not recommend using Adobe Photoshop for designing figures, but we can accept Photoshop generated (.PSD or .TIFF) files only if each element included in the figure (text, labels, pictures, graphs, arrows and scale bars) are on separate layers. All text should be editable in 'type layers' and line-art such as graphs and other simple schematics should be preserved and embedded within 'vector smart objects' - not flattened raster/bitmap graphics.

- Some programs can generate Postscript by 'printing to file' (found in the Print dialogue). If using an application not listed above, save the file in PostScript format or email our Art Editor, Allen Beattie for advice (a.beattie@nature.com).

Regardless of format, all figures must be vector graphic compatible files, not supplied in a flattened raster/bitmap graphics format, but should be fully editable, allowing us to highlight/copy/paste all text and move individual parts of the figures (i.e. arrows, lines, x and y axes, graphs, tick marks, scale bars etc.). The only parts of the figure that should be in pixel raster/bitmap format are photographic images or 3D rendered graphics/complex technical illustrations.

All placed images (i.e. a photo incorporated into a figure) should be on a separate layer and independent from any superimposed scale bars or text. Individual photographic images must be a minimum of 300+ DPI (at actual size) or kept constant from the original picture acquisition and not decreased in resolution post image acquisition. All colour artwork should be RGB format.

FIGURE LEGENDS – must not exceed 350 words for each figure to allow fit on a single printed NCB page together with the figure. They must include a brief title for the whole figure, and short descriptions of each panel with definitions of the symbols used, but without detailing methodology.

TABLES – main tables should be provided as individual Word files, together with a brief title and legend. For supplementary tables see below.

SUPPLEMENTARY INFORMATION – Supplementary information is material directly relevant to the conclusion of a paper, but which cannot be included in the printed version in order to keep the manuscript concise and accessible to the general reader. Supplementary information is an integral part of a Nature Cell Biology publication and should be prepared and presented with as much care as the main display item, but it must not include non-essential data or text, which may be removed at the editor's discretion. All supplementary material is fully peer-reviewed and published online as part of the HTML version of the manuscript. Supplementary Figures and Supplementary Notes are appended at the end of the main PDF of the published manuscript.

Supplementary items should relate to a main text figure, wherever possible, and should be mentioned sequentially in the main manuscript, designated as Supplementary Figure, Table, Video, or Note, and numbered continuously (e.g. Supplementary Figure 1, Supplementary Figure 2, Supplementary Table 1, Supplementary Table 2 etc.).

Unprocessed scans of all key data generated through electrophoretic separation techniques need to be presented in a supplementary figure that should be labelled and numbered as the final supplementary figure, and should be mentioned in every relevant figure legend. This figure does not count towards the total number of figures and is the only figure that can be displayed over multiple pages, but should be provided as a single file, in PDF or TIFF format. Data in this figure can be displayed in a relatively informal style, but size markers and the figures panels corresponding to the presented data must be indicated.

The total number of Supplementary Figures (not including the "unprocessed scans" Supplementary Figure) should not exceed the number of main display items (figures and/or tables (see our Guide to Authors and March 2012 editorial http://www.nature.com/ncb/authors/submit/index.html#suppinfo; http://www.nature.com/ncb/journal/v14/n3/index.html#ed). No restrictions apply to Supplementary Tables or Videos, but we advise authors to be selective in including supplemental data.

Each Supplementary Figure should be provided as a single page and as an individual file in one of our accepted figure formats and should be presented according to our figure guidelines (see above). Supplementary Tables should be provided as individual Excel files. Supplementary Videos should be provided as .avi or .mov files up to 50 MB in size. Supplementary Figures, Tables and Videos much be accompanied by a separate Word document including titles and legends.

GUIDELINES FOR EXPERIMENTAL AND STATISTICAL REPORTING

REPORTING REQUIREMENTS – We are trying to improve the quality of methods and statistics reporting in our papers. To that end, we are now asking authors to complete a reporting summary that collects information on experimental design and reagents. The Reporting Summary can be found here https://www.nature.com/documents/nr-reporting-summary.pdf)If you would like to reference the guidance text as you complete the template, please access these flattened versions at http://www.nature.com/authors/policies/availability.html.

STATISTICS – Wherever statistics have been derived the legend needs to provide the n number (i.e. the sample size used to derive statistics) as a precise value (not a range), and define what this value represents. Error bars need to be defined in the legends (e.g. SD, SEM) together with a measure of centre (e.g. mean, median). Box plots need to be defined in terms of minima, maxima, centre, and percentiles. Ranges are more appropriate than standard errors for small data sets. Wherever statistical significance has been derived, precise p values need to be provided and the statistical test used needs to be stated in the legend. Statistics such as error bars must not be derived from n<3. For sample sizes of n<5 please plot the individual data points rather than providing bar graphs. Deriving statistics from technical replicate samples, rather than biological replicates is strongly discouraged. Wherever statistical significance has been derived, precise p values need to be provided and the statistical test stated in the legend.

Information on how many times each experiment was repeated independently with similar results

needs to be provided in the legends and/or Methods for all experiments, and in particular wherever representative experiments are shown.

We strongly recommend the presentation of source data for graphical and statistical analyses as a separate Supplementary Table, and request that source data for all independent repeats are provided when representative experiments of multiple independent repeats, or averages of two independent experiments are presented. This supplementary table should be in Excel format, with data for different figures provided as different sheets within a single Excel file. It should be labelled and numbered as one of the supplementary tables, titled "Statistics Source Data", and mentioned in all relevant figure legends.

--------- Please don't hesitate to contact NCB@nature.com should you have queries about any of the above requirements ---------

## Author Rebuttal to Initial comments

We would like to thank the Reviewers for their enthusiasm about our work, judicious comments and many thoughtful suggestions. Below is our detailed point-by point response (our responses are in bold and the original comments in their entirety are in italics). The referee reports have been very helpful and we hope that the Reviewers will now find the revised version of our manuscript suitable for publication in Nature Cell Biology.

Reviewers' Comments:

*Reviewer #1:*
*Remarks to the Author:*
*The manuscript by Prosser et al describes a new molecular pathway that facilitate aggresome formation in response to proteotoxic stress. This pathway utilises the centrosomal components CP110, CEP290 and CEP97 to seed the aggresome, which then expands via recruitment of centriolar satellites. Furthermore, the authors find that CP110 is rate-limiting for aggresome formation in senescent cells and that HTTPolyQ inclusions also require the CP110-CEP97-CEP290 and PCM1-containing satellites. Altogether the results reported here expose a vital contribution of centrosomes and centriolar satellites to aggresome assembly.*

*The manuscript is written in a clear and concise manner with data quality and presentation being excellent. I anticipate the topic to be of interest to the wider cell biology community encompassing multiple fields and is thus well-suited for Nature Cell Biology.*

We thank the Reviewer for the positive feedback, it is very much appreciated.

*Specific points:*

*1. I find the link between centriolar satellites and aggresome assembly compelling. However, it seems surprising that knockouts of PCM1, AZI1, CCDC14, PIBF1/CEP90, all impact aggresome formation to the same degree, despite affecting satellite formation and distribution in very different ways. Therefore, it would be important to identify one or two satellite components that do not influence aggresome formation.*

This point is well taken. We now provide quantification of aggresome formation in FOP (Mojarad *et al*., 2017) and OFD1 (this study) KO cells. We found that aggresome formation in the absence of either of these satellite components was comparable to WT cells (new Supplementary Fig. 3g). These results are presented on Page 8 of the revised manuscript.

We were also surprised that KO of PCM1, AZI1, CCDC14, KIAA0753 and PIBF1 impacted aggresome assembly to the same extent. To examine this further, we questioned whether autophagy was altered in these cell lines when they were subjected to proteasome inhibition. Monitoring autophagic activity via LC3-II levels, we observed a stronger autophagy response in each of the KOs as compared to WT cells when treated with MG132 (new Supplementary Fig. 4d). Clearance of protein aggregates via autophagy would reduce their availability for accumulation into the aggresome, thereby reducing aggresome size. This is demonstrated in new Fig. 4b,c where activation of autophagy with an mTor inhibitor (KU-0063794) in WT cells reduced aggresome size to that seen in PCM1 KOs. Conversely, inhibition of autophagy in the AZI1, CCDC14, KIAA0753 and PIBF1 KOs significantly increased the size of the aggresome forming in these cells (Supplementary Fig. 4e,f). However, in the PCM1 KO, aggresome size was only partly rescued in the presence of an autophagy inhibitor (new Fig. 4b,c). This suggested that it is the presence of centriolar satellite structures (which are absent in the PCM1 KO) that is crucial to aggresome assembly. To support this, PCM1 was depleted in KIAA0753 and PIBF1 KO cells prior to treatment with autophagy inhibitors, which prevented the rescue of aggresome size seen in control cells (new Fig. 4e,f). From this we conclude that there is a requirement for PCM1-containing centriolar satellites in aggresome assembly. These results are discussed on Page 10 of the revised manuscript.

*2. What is the consequence of cells not being able to assemble larger aggresomes in the satellite knockout cell lines?*

To look at the consequence of cells not forming larger aggresomes in the satellite KO lines, we used clonogenic survival assays as a method to quantify cell death and fitness in response to proteasome inhibition. Untreated cells had a similar proliferative capacity as WT cells (new Supplementary Fig. 4a,b), however the PCM1 KO line had a lower survival rate following treatment with MG132 for 5 hours as compared to the other cell lines. Aggresomes are cleared, at least in part, by autophagy (Hao et al., 2013) and the increased rate of autophagy seen in the satellite KO lines (as described under Point 1, above) potentially aids the clearance of toxic protein aggregates that would otherwise reduce cell survival following proteasome inhibition. The PCM1 KO line similarly displays increased autophagy during proteasome inhibition, therefore its reduced survival is interesting. PCM1 has previously been

reported to regulate autophagy via GABARAP (Joachim et al., 2017), therefore it is plausible to imagine that autophagic clearance of aggresomes is somewhat disrupted in the PCM1 KO as compared to the other KOs that still contain PCM1-positive satellites. These findings are now discussed on Page 9 of the manuscript.

*Is there any data from such cell lines that baseline proteotoxic stress is elevated?*

This is a good question and to our knowledge no such data exists. Therefore, to examine baseline proteotoxic stress, we assessed the levels of heat shock proteins (HSPs) in WT and PCM1 KO cells treated with DMSO or MG132 (new Supplementary Fig. 4c). None of the HSPs examined were elevated in the PCM1 KO as compared to the DMSO control. In MG132 treated cells, HSP70 and HSP40 (DNAJB1) levels were elevated in both WT and PCM1 KO cells, indicative of proteotoxic stress in these cells in response to proteasome inhibition. These results are presented on Page 9 of the manuscript.

*Do PCM1 null cells exhibit signs of senescence or limited proliferative capacity?*

RPE-1 cells are immortalized so it was not possible to examine senescence in our existing PCM1 KO line. To circumvent this, we turned to the human primary fibroblast cell line IMR-90 and generated PCM1 and FAM83G (control; Steinhart *et al*., 2017) KO populations using a lentiCRISPR system. By monitoring the onset of senescence using the senescence-associated βgalactosidase activity assay we saw no difference between the two cell lines as shown on Page 14 of the revised manuscript (new Supplementary Fig. 7e,f). Additionally, the DMSO treated controls from the clonogenic assays (new Supplementary Fig. 4a,b) support that there is no difference in proliferative capacity between WT and PCM1 KO cells.

*3. Aggresome size is a function of its assembly and clearance. Could the authors show a time course as to how these aggresomes behave over time in cells that lack the CEP290/CP110 module or satellites? For instance, do they grow larger if MG132 treatment is continued beyond 5 hr, or is the size shown the limit? This does not need to be live, it would be interesting to see average sizes across the population at different timepoints.*

This was a great suggestion. We now show a timecourse of aggresome size in WT and PCM1 KO RPE-1 cells in new Fig. 4g,h and the same in siGL2 and siCP110 treated RPE-1 cells in new Fig. 6h,i. In both the PCM1 KO and siCP110 cells we see that the aggresome reaches its maximum size by 3-5 hours and does not increase in size beyond this timepoint. This contrasts with control cells where it continues to grow up until 10 hours of MG132 treatment.

Further to this, we looked at the assembly of larger aggregates in malignant melanoma A-375 cells with 16 hours of MG132 treatment (treatments beyond 10 hours were not well tolerated by RPE-1 cells, leading to extensive cell death). In both siPCM1 cells (new Fig. 4i,j) and siCP110 cells (new Fig. 6j,k), large aggresomes did not accumulate with 16 hours of MG132 treatment, despite the clear accumulation of protein aggregates in these cells. Together, these results presented on Pages 10 and 13, support that

disruption of PCM1 and CP110 blocks aggresome assembly at the centrosome at an early point in the pathway.

*4. The data on GFP-CP110 overexpression in senescent cells (Fig 6f) is certainly interesting but the conclusion that restoration of CP110 levels in senescence is sufficient for MG132-induced aggresome formation is not fully supported by the results. GFP-CP110 overexpression indeed leads to accumulation of pHSP27 around the centrosomal region. However, the scattered granular cytoplasmic GFP-CP110 signal largely co-localises with pHSP27 in absence of MG132, and therefore I wonder if what we see are GFP-CP110 aggregates that associate with pHSP27, rather than true aggresomes. This caveat should be mentioned.*

We have mentioned this caveat as requested by the Reviewer on Page 14. To support that the GFPCP110 aggregates are indeed aggresomes we now provide images of p62, HSP70 and $Ub^+$ proteins co-localizing with the GFP-CP110 aggregates in senescent cells (new Fig. 7f).

To provide additional support and mechanistic insight for CP110 overexpression in senescent cells restoring aggresome formation, we examined the ability of two mutant forms of CP110 to rescue aggresome assembly in these cells. The R586A,L588A mutation is reported to abolish the interaction between CP110 and CCNF (D'Angiolella et al., 2010), while deletion of amino acids 6782 disrupts the interaction between CP110 and CEP290 (Tsang et al., 2008). For the R586A,L588A mutant, aggresome assembly was restored to a similar rate as the WT CP110, however pHSP27 was restricted to the centrioles in cells expressing the CEP290-binding mutant (new Fig. 7g,h). This suggests CP110 overexpression can only restore aggresome assembly in senescent cells if it is able to interact with CEP290. These data are discussed on Page 15 of the revised manuscript.

*A similar co-localisation between pHSP27 with cytoplasmic CEP290 aggregates is also seen with FLAGCEP290 overexpression in Sup Fig 5. Because many centrosomal proteins form large cytoplasmic aggregates/polymers when overexpressed the authors should be careful to distinguish these from aggresomes. Using additional aggresome markers could be useful in this regard.*

This is a good point and we now provide images that show co-localization between the MG132induced FLAG-CEP290 aggregates and HSP70, p62 and $Ub^+$ proteins (new Supplementary Fig. 6k). Additionally, vimentin staining revealed the rearrangement of intermediate filaments around some of the aggregates, a characteristic that has been reported in instances of aggresome formation (Johnston et al., 1998). These results can be found on Page 13 of the revised manuscript.

*In addition, expression of many proteins is reduced in senescence and so a reduction in CP110 and CEP290 levels is not entirely unexpected. A more thorough analysis of centriolar/PCM/satellite protein levels would be necessary to support the hypothesis that CP110 levels are limiting in senescence for aggresome formation.*

This is a very good point. Indeed, both the transcriptome and proteome are reported to change when cells enter senescence (Gorgoulis et al., 2019; Delfarah et al., 2021), with the levels of many proteins increasing or decreasing. We now provide western blots of AZI1, CCDC14, KIAA0753, PCM1 and PIBF1 levels in cycling and senescent cells (new Fig. 7c). The level of many of these proteins are reduced in senescent cells, with the exception of AZI and PCM1. PCM1 staining confirms the presence of centriolar satellites in these cells (Fig. 7a). Given that loss of AZI1, CCDC14, KIAA0753 and PIBF1 influence aggresome size through increased autophagy (see Point 1 above), and that LC3-II levels appeared comparable between cycling and senescent HFF-1 cells (new Fig. 7c), we focused on the impact of CP110 in senescent cells.

We believe that our results further support the notion that CP110 levels are limiting with regard to aggresome formation in senescent cells. Indeed, we show that overexpression of CP110 is able to rescue the accumulation of pHSP27, p62, HSP70, CEP97, CEP290 and PCM1 (new Fig. 7f and new Supplementary Fig. 7i). Furthermore, as discussed above, a CP110 mutant lacking the CEP290 interacting domain was unable to rescue aggresome assembly in senescent cells (new Fig. 7 g,h), suggesting that CP110 can only promote aggresome formation if it is able to bind to CEP290. This is discussed on Page 14 of the revised manuscript.

*5. The authors should elaborate further in the discussion on why they think the CEP290/CP110/CEP97 module is responsible for aggresome assembly. Is it because they are still transported to centrosomes in a misfolded state, possibly in a complex?*

This is a good point and we have provided further discussion on the role the CP110-module might play in aggresome assembly, including the possibility that it is transported to the centrosome in a misfolded state that potentiates aggresome formation. We also raise the possibility that they form a site on centrioles that is required for the initial assembly of aggresomal proteins, forming a foundation onto which further proteins accumulate, or that they act as adaptors between centriolar satellites and aggresomal cargo to direct their transport to the centrosome. This is elaborated in the discussion on Page 17 and incorporated into our revised model (Fig. 8k).

*Do GFP-CP110 and FLAG-CEP290 aggregates contain CEP290, CP110 and CEP97?*

This is an interesting question which we have now pursued. The GFP-CP110 aggresomes do indeed contain CEP290 and CEP97 (new Supplementary Fig. 7i). For FLAG-CEP290, we could not detect CP110 nor CEP97 in the cytoplasmic aggregates of FLAG-CEP290 that accumulated during proteasome inhibition (new Supplementary Fig. 6k). However, the aggregates do accumulate aggresome markers, such as HSP70, p62 and ubiquitinated proteins (new Supplementary Fig. 6k). Additionally, FLAG-CEP290 expression acts in a dominant-negative manner on the accumulation of CP110, CEP97 at the centrosome during proteasome inhibition, suggesting that overexpression of CEP290 disrupts the CP110-CEP97-CEP290 module, preventing aggresome formation at the centrosome and sequestering aggresomal

components in the cytoplasm (new Supplementary Fig. 6k). This data is included on Page 13 of the revised manuscript.

*Have the authors investigated contribution by Talpid3, which also interacts with CEP290?*

We had not previously looked at Talpid3, but now include quantification for aggresome formation in cells depleted of Talpid3 in new Supplementary Fig. 6d. In line with the other CP110-module components, we found that depletion of Talpid3 also reduced aggresome size. This data is discussed on Page 13 of the revised manuscript.

*A western blot that shows changes in levels of these components after MG132 treatment would be useful to include. Perhaps, this complex drives aggresome assembly because stoichiometry of its components is particularly sensitive to MG132 treatment.*

We now provide a western blot of total CP110, CEP97 and CEP290 levels in control and MG132 treated cells in new Supplementary Fig. 6a.

*It would be useful to include any centrosome protein knockout that does not impact on aggresome formation.*

As requested under Point 1 above, we now provide quantification of aggresome formation in FOP (Mojarad *et al*., 2017) and OFD1 (this study) KO cells, which in addition to being centriolar satellite proteins also localize to the centrioles. Alongside this we also include quantification from CETN2 KOs (Prosser and Morrison, 2015), a centriolar protein that has been reported to interact with CP110 (Tsang et al., 2006). Each of these proteins (FOP, OFD1 and CETN2) localize to the aggresome (Fig. 1d,f), however their KO does not impede aggresome formation (new Supplementary Fig. 3g). These results are presented on Page 8 of the revised manuscript.

*6. The paper contains a lot of new information and would benefit from a more detailed discussion.*

We agree and have now provided a more detailed discussion.

*7. Line 319: Thereby, assigning…*
*This seems to be an incomplete sentence.*

We thank the Reviewer for pointing this out, we have now corrected this in the text.

*Reviewer #2:*
*Remarks to the Author:*

*In this manuscript, Prosser et al. provide evidence that centriolar components are required to form the perinuclear inclusion, aggresomes. By employing higher resolution microscopy, it was shown that selective centriolar and pericentriolar (PCM) proteins are expanded from typical two centriolar spots into multiple foci within the areas occupied by aggresomes (marked by pHsp27) induced by a proteasome inhibitor, MG132. Systematic knockdown of known components of centrioles and centriolar satellite showed that selective centriolar proteins, including PCM-1 as well the CP110-CEP97-CEP290 module, are required for MG132-induced aggresome and mutant Htt103Q inclusion formation.*
*In the last two decades, significant progress has been made on the origin of protein aggregates that constitute aggresomes and the machinery involved in the dynamic concentration and clearance of aggresome at the MTOC.*

*The identification of centriolar satellite components as a new component involved in aggresome formation adds another level of understanding to cellular proteostasis. However, there are two main issues with the current report. First, although genetic knockdown of centrosome satellite components can affect the formation or size of aggresomes, there is no clear mechanistic picture of how these factors might affect the production, transport, or processing of protein aggregates associated with aggresome biogenesis.*

We thank the Reviewer for making this point and for encouraging us to investigate this. Focusing our attention on the production, transport and processing of protein aggregates in the KO cell lines has allowed us to significantly advance our study in this area. By performing cellular fractionation to ascertain which proteins reside in the soluble and insoluble fractions of WT and PCM1 KO cell lines treated with MG132 has allowed us to deduce that insoluble protein aggregates are produced in the PCM1 KO during proteasome inhibition (new Fig. 4d), with HSP70, p62 and HSP27 being found in the insoluble fraction of MG132 treated cells.  This is supported by the appearance of pHSP27 and p62 positive aggregates in the cytoplasm of PCM1 KO cells treated with MG132 (new Fig. 4c,f,g,j). This is included on Page 10 of the revised manuscript.

Further to this, we observed a stronger induction of autophagy in the AZI1, CCDC14, KIAA0753 and PIBF1 KO cells treated with MG132 when compared to WT cells (new Supplementary Fig. 4d). This led us to investigate whether inhibition of autophagy would rescue aggresome formation in these cells. Indeed, we found this to be the case for the AZI1, CCDC14, KIAA0753 and PIBF1 KO cell lines (new Supplementary Fig. 4e,f). This suggests that the processing of protein aggregates in these cells is directed to autophagy rather than aggresome formation. In contrast, there was only a moderate rescue of aggresome size in the PCM1 KO line when autophagy was inhibited (new Fig. 4b,c), while activation of autophagy in WT cells reduced aggresome size to that seen in the PCM1 KO. These new data are presented on Page 10 of the revised manuscript.

As the AZI1, CCDC14, KIAA0753 and PIBF1 KO cell lines contain centriolar satellites, as compared to the PCM1 KO that does not, we wondered whether satellites contributed to the recovery of aggresome size seen when autophagy was inhibited in these cell lines. To address that, we depleted PCM1 in the

KIAA0753 and PIBF1 KOs and found that this blocked the increase in aggresome size seen in these cells when autophagy was inhibited (new Fig. 4e,f). This new data is discussed on Page 10 of the revised manuscript. Taken together, this supports a role for centriolar satellites in the transport of protein aggregates to the aggresome. This is strengthened by the observation that centriolar satellites associate with $Ub^+$ aggregates in the cytoplasm of cells with depolymerized microtubules (Fig. 2l) and the localization of centriolar satellites to the outside of the pHSP27 ring structures that assemble during aggresome formation, as revealed by super-resolution microscopy (new Fig. 3d).

Similar analysis in cells depleted of CP110-module components revealed that insoluble protein aggregates form in these cells (new Fig. 6h,j and new Supplementary Fig. 6l). However, autophagy was not increased (new Supplementary Fig. 6m) and inhibition of autophagy did not rescue aggresome size in cells depleted of CP110 (new Supplementary Fig. n,o). This suggests that the CP110-module is either involved in the transport of protein aggregates to the centrosome or functions at the centrosome to assemble protein aggregates into an aggresome. To expand on this, we identified the proximity interactors of CP110 under conditions of proteasome inhibition, revealing an enrichment of pathways associated with the proteasome and protein aggregation diseases (new Supplementary Fig. 8 g-k; full protein list provided in new Supplementary Table 2). This new data is discussed on Page 16 of the revised manuscript.

These important lines of investigation proposed by this Reviewer have allowed us to build a clearer mechanistic picture of the intersect between the CP110 module, centriolar satellites and autophagy, and how this contributes to the processing of protein aggregates during aggresome biogenesis.  These finding are now elaborated in the discussion on Pages 16-19 and incorporated into a revised model (Fig. 8k).

*Second, the definition of aggresomes is some analysis would need more detailed analysis. For example, the conclusion that cyclin F (CCNF) inactivation leads to spontaneous aggresome formation requires more careful analysis. The appearance of Ub+ foci or CETN2 foci (> 4 in numbers) was interpreted as spontaneous aggresome formation upon CCNF knockdown. However, it is unclear whether this conclusion is accurate, as it is difficult to judge what the CETN2 foci are and whether they genuinely reflect a significant accumulation of protein aggregates. As aggresomes are generally thought to arise from peripheral aggregates tagged by p62, the identity of CETN2 foci needs additional protein aggregate/inclusion markers, such as p62. The definition of aggresomes is a potential issue throughout the report (See points 2 and 3 for details).*

We apologize to the Reviewer for our misuse of terminology which led to us failing to make ourselves clear as to what we think the additional foci forming in CCNF-depleted cells represents. Our intention was to suggest that depletion of CCNF leads to the accumulation of proteins onto centriolar satellites, which might act as aggresome precursors. We have cleared this confusion up in the text on Pages 11-12. Additionally, we now include full cell images of both $Ub^+$ and p62 staining in cells depleted of CCNF (new

Fig. 5h), showing an increase in foci of these markers throughout the cell and in the vicinity of the centrosome. For Points 2 and 3, please see our specific responses below, which we hope satisfactorily address the concerns the Reviewer has with regard to our molecular definition of aggresomes in these instances.

*In conclusion, although the identification of centriolar machinery is in aggresome formation is novel, the lack of significant mechanistic insight and the unconventional method to define aggresomes makes it difficult to judge the key findings' importance or relevance. With these significant deficiencies, this report, in its current form, is not suitable for Nature Cell Biology.*

We are glad the Reviewer views our findings as novel. We have made significant advances in providing mechanistic insight on the role played by the CP110 module and centriolar satellites in aggresome assembly and have addressed the concerns with regard to aggresome definition as detailed above and in the specific points below. We hope the Reviewer now finds our work suitable for publication.

*Other comments:*
*1. The relationship between the "centriolar" foci and aggresome (protein aggregates) needs to be better defined. For example, in Fig 1, the relationship between satellite markers and pHsp70 is challenging to grasp from one representative image. Are satellite foci and pHsp27 foci always over-lapping with each other? A more quantitative co-localization analysis would be helpful.*

We agree that it is hard to decipher the precise localization of individual proteins in the aggresome using conventional microscopy. To provide more information on the localization of centriolar, satellite and aggresome proteins within the same structure we performed super-resolution imaging on the centriole markers CEP135, CP110 and CEP120, satellite markers PCM1, PIBF1 and CEP290 and the aggresome markers pHSP27, p62 and $Ub^{+}$ (new Fig. 1g and new Supplementary Fig. 1e). From these images, we performed pair-wise analysis of co-localization between proteins by calculating the Pearson correlation coefficient (new Fig. 1h and new Supplementary Fig. 1f). From these results we see a high-degree of co-localization between the aggresome proteins and centriolar satellites, while the proteins that remain restricted to the centrioles (such as CEP135) have much lower overlap. These results are discussed on Page 6 of the revised manuscript.

*2. The size of aggresomes in this report appears to be small compared with previous reports. It was noted that aggresomes were induced by proteasome inhibitor treatment for 5 hrs. This is a relatively short treatment to induce aggresomes for standard imaging and biochemical analysis (typical treatment time is ~ 24 hrs). Because of the concern raised in point 1, it would be reassuring to induce large aggresomes by longer inhibitor treatment and then investigate the distribution of representative PCM and satellite markers and their knockdown effect.*

To address this issue, we tried subjecting RPE-1 cells to longer MG132 treatment but found that it was not well tolerated beyond 10 hours, leading to extensive cell death. We postulate that, as a

nontransformed cell line, RPE-1 cells display greater sensitivity to proteasome inhibition than the cancer-derived cell lines more routinely used to study aggresomes. Despite this, we did examine RPE-1 cells in timecourse experiments up to 10 hours in duration and observed an increase in aggresome size in control cells up to this timepoint (new Fig. 4g,h and new Fig. 6 h,i). In contrast, aggresomes in PCM1 KO cells (new Fig. 4g,h) and cells treated with siCP110 (new Fig. 6h,i) reached their maximum size by 3-5 hours and did not expand in size beyond this point.

To induce the formation of larger aggresomes, we turned to the A-375 human melanoma cell line, which we treated for 5 and 16 hours with MG132. As presented in new Fig. 1e, at 5 hours aggresomes containing pHSP27, PCM1, p62 and CP110 assembled to a similar size as those in RPE1 cells at the same timepoint. At 16 hours, larger accumulations of pHSP27 and p62 formed and were concentrated around smaller assemblies of PCM1 and CP110 at the centrosome. These smaller assemblies were comparable in size to those formed at 5 hours treatment, suggesting that the smaller inclusions form first and, as more protein aggregates accumulate, they assemble around this original structure. Strikingly, when PCM1 or CP110 was depleted in A-375 cells, the assembly of both these smaller and larger structures was impeded, despite protein aggregates accumulating in these cells (new Fig. 4 i,j and new Fig. 6 j,k). These observations support the notion that the smaller inclusion forms first to facilitate accumulation of protein aggregates and are now presented on Pages 6, 11, and 14 of the manuscript.

*3. Fig 6E and F have a similar issue of defining aggresomes in senescent cells transfected with GFPCP110. The size of the aggresome is very small in both DMSO and MG132 treated transfected cells. Therefore, it is very difficult to conclude that GFP-CP110 can promote aggresome formation in senescent cells. Because transfected and overexpressed proteins could sometimes become aggregated upon MG132 treatment, a proper GFP-CP110 mutant control should be included to guard against this potential pitfall.*

We appreciate the Reviewer raising this concern. To provide further support for CP110 overexpression in senescent cells restoring aggresome formation, we examined the ability of two mutant forms of CP110 to rescue aggresome assembly in these cells. The R586A,L588A mutation is reported to abolish the interaction between CP110 and CCNF (D'Angiolella et al., 2010), while deletion of amino acids 67-82 disrupts the interaction between CP110 and CEP290 (Tsang et al., 2008). For the R586A,L588A mutant, aggresome assembly was restored to a similar rate as the WT construct, however pHSP27 was restricted to the centrioles in cells expressing the CEP290-binding mutant (new Fig. 7g,h). This suggests CP110 overexpression can only restore aggresome assembly in senescent cells if it is able to interact with CEP290. These new data are presented on Page 13 of the revised manuscript.

Additionally, to better define the aggresome as suggested by this Reviewer, we now provide images of p62, HSP70 and $Ub^{+}$ proteins co-localizing with the GFP-CP110 aggregates in senescent cells (new Fig. 7f). This is presented on Page 12 of the revised manuscript.

*4. Almost all images presented in the study only show a small cell region corresponding to the MTOC, where aggresomes typically form. This presentation might lose some vital information as protein aggregates accumulate at the cell periphery before their concentration. It would be informative to present images covering the entire cells in key figures, particularly in figures where inclusion formation was suppressed by PCM1 or CP110/CEP97/CEP290 knockdown.*

We thank the Reviewer for highlighting this issue. We now present images that capture the full cell in many figures in the manuscript, drawing particular attention to those illustrating key experiments in new Fig. 4 c,f,g,i; new Fig. 6 h,j; new Supplementary Fig. 4f; and new Supplementary Fig. 6o. Furthermore, we have included both pHSP27 and p62 staining in these full cell images to provide a better molecular definition and insight into the accumulation of protein aggregates in the cytoplasm of cells depleted of PCM1 or components of the CP110-module. This has allowed us to ascertain that aggregates do indeed form in these cells and that it is their concentration at the centrosome that is impeded. We would like to include full cell images for everything in the manuscript, but this is not possible due to space restrictions. We hope what we now provide is sufficient to satisfy the Reviewer.

*5. The effects of PCM-1 and CP110/CEP97/CEP290 knockdown on mutant HTT inclusion formation is interesting (fig 6h), but proper evaluation of the expression levels of GFP-HTT in both soluble and insoluble fractions is needed. The inclusion of independent biochemical analyses on protein aggregate processing would strengthen the conclusions deduced from image analyses.*

We agree that this is a very important experiment to include so, to that end, we performed biochemical fractionation studies that allow us to discriminate between the soluble and insoluble proteins from PCM1 KO cells and siCP110-treated cells transfected with GFP-HTT97Q (new Fig. 8 d and i). Short polyQ domains (<35 Qs) are soluble, while longer polyQ domains (>36 Qs) tend to aggregate (Adegbuyiro *et al*., 2017). Our analysis revealed GFP-HTT97Q separated comparably to the soluble and insoluble fractions in the KO and KD cells as compared to controls. In contrast, GFP-HTT25Q was restricted to the soluble fraction in both WT and PCM1 KO cells (new Supplementary Fig. 8b). These results suggest that GFP-HTT97Q is able to form insoluble aggregates in PCM1 KO and siCP110 cells, but their incorporation into a single inclusion is impeded. To support this, we examined PCM1 KO and siCP110 cells expressing GFP-HTT97Q with saturating imaging conditions and could now observe the formation of many small cytoplasmic aggregates in a proportion of these cells (new Fig. 8 e and j). These results are presented on Page 15 and reflected in our revised model (new Fig. 8l).

*6. The effect of Hsp27 knockdown on suppressing aggresome formation has not been described previously (Supplementary fig 2). To ensure the effect of knockdown is specific, a genetic rescue experiment with Hsp27 expression plasmid is recommended.*

We thank the Reviewer for bringing this to our attention. To address this, we generated an inducible cell line that would express an siRNA resistant version of HSP27 upon addition of tetracycline to the media. We depleted HSP27 by siRNA for 24 hours before induction for a further 24 hours. For the last 5 hours, cells were treated with MG132. siRNA resistant HSP27 was able to restore aggresome formation as measured by pHSP27 incorporation and the accumulation of $Ub^{+}$ at the centrosome (new Supplementary Fig. 2 c-e) and is now discussed on Page 6 of the revised manuscript.

*Reviewer #3:*
*Remarks to the Author:*
*In the manuscript Prosser an colleagues investigates mechanisms of aggresome formation. They use high resolution microscopy to understand how components of centrosome affect aggresome formation. They demonstrate that components of the satellites are critical for the process. Furthermore, they identified components of the centrosome that control aggresome formation in response to proteasome inhibitors. Finally the demonstrate that downregulation of these components in senescence is responsible for poor induction of aggresome under these conditions. Overall the work is very original and novel. The quality of data is superb, and appropriate statistics has been provided.*
*Overall the story is very interesting and significantly advances the field.*

We thank the Reviewer for the positive feedback, it is very much appreciated.

*There are few minor problems that I would like to comment on.*
*1. Light yellow schemes as in Fig. 2D are difficult to see.*

We agree with the Reviewer and have now replaced all such plots with an alternative colour scheme which makes it easier to see all the data points.

2. *It seems that HDAC6 had relatively minor effect on aggresome. This point needs to be highlighted.* We

have added this point to the text on Page 7.

3. *When talking about effects of CCNF, it would be important to show a cell with aggresome at lower resolution. Otherwise it is not clear if depletion of CCNF indeed cause aggresome formation in the absence of proteasome inhibitors or just increases the number of foci.*

We now include, in new Fig. 5h, whole cell views of siCCNF and control treated cells stained for ubiquitinated proteins and p62. There is indeed an increased number of foci with these two markers as compared to control cells, some of which co-localize with centriolar satellites in the centrosomal region. This is discussed on Page 12.

*4. There is a controversy about the relevance of Htt inclusion body and aggresome. It would be important to test if microtubular transport is necessary for the process or centriole just forms a nucelation site for Htt and soluble molecules join the inclusion body by simple diffusion.*

This point is well taken. In line with previous studies (Muchowski et al., 2002; Kaminosono et al., 2008), we found that microtubule depolymerization prevents the accumulation of Htt aggregates into a single inclusion body, with smaller aggregates seen throughout the cytoplasm (new Supplementary Fig. 8c). By contrast, multiple larger inclusions were seen in cells which lack a functional centrosome (STIL KO; new Supplementary Fig. 8e). Together, these results suggest that both microtubules and centrosomes are important in the focusing of HTT aggregates into a single, larger inclusion. These results are presented on Page 16 of the revised manuscript and incorporated into our revised model (Fig. 8l).

*References provide appropriate credit to previous work. The paper is very well written.*

We thank the Reviewer for these positive comments.

References

Adegbuyiro, A. *et al*. Proteins Containing Expanded Polyglutamine Tracts and Neurodegenerative Disease. *Biochemistry* 56, 1199–1217 (2017).

D'Angiolella, V. *et al.* SCFCyclin F controls centrosome homeostasis and mitotic fidelity through CP110 degradation. *Nature* 466, 138–142 (2010).

Delfarah, A. *et al*. Identification of a Proteomic Signature of Senescence in Primary Human Mammary Epithelial Cells, *bioRxiv*, 2020.09.22.309351 (2021).

Gorgoulis, V. *et al*., Cellular Senescence: Defining a Path Forward. *Cell* 179, 813–27 (2019).

Hao, R. *et al.* Proteasomes Activate Aggresome Disassembly and Clearance by Producing Unanchored Ubiquitin Chains. *Molecular Cell* 51, 819–828 (2013).

Joachim, J. *et al.* Centriolar Satellites Control GABARAP Ubiquitination and GABARAP-Mediated Autophagy. *Curr. Biol.* 27, 2123–2136.e7 (2017).

Johnston, J. A., Ward, C. L. & Kopito, R. R. Aggresomes: a cellular response to misfolded proteins. *J. Cell Biol.* 143, 1883–1898 (1998).

Kaminosono, S. *et al.* Suppression of mutant Huntingtin aggregate formation by Cdk5/p35 through the effect on microtubule stability. *J Neurosci* 28, 8747–8755 (2008).

Mojarad, B.A. *et al*. CEP19 Cooperates with FOP and CEP350 to Drive Early Steps in the Ciliogenesis Programme. *Open Biology* 7, 170114 (2017).

Muchowski, P. J., Ning, K., D'Souza-Schorey, C. & Fields, S. Requirement of an intact microtubule cytoskeleton for aggregation and inclusion body formation by a mutant huntingtin fragment. *Proc Natl Acad Sci USA* 99, 727–732 (2002).

Prosser, S.L. and Morrison, C.G. Centrin2 Regulates CP110 Removal in Primary Cilium Formation. *The Journal of Cell Biology* 208, 693–701 (2015).

Steinhart, Z. *et al*. Genome-Wide CRISPR Screens Reveal a Wnt-FZD5 Signaling Circuit as a Druggable Vulnerability of RNF43-Mutant Pancreatic Tumors. *Nature Medicine* 23, 60-68 (2017).

Tsang, W. Y. *et al.* CP110 cooperates with two calcium-binding proteins to regulate cytokinesis and genome stability. *Mol. Biol. Cell* 17, 3423–3434 (2006).

Tsang, W. Y. *et al.* CP110 Suppresses Primary Cilia Formation through Its Interaction with CEP290, a Protein Deficient in Human Ciliary Disease. *Dev. Cell* 15, 187–197 (2008).

## Decision Letter, first revision:

Dear Dr Pelletier,

I apologize for the delay. Your manuscript, "The CP110-CEP97-CEP290 module orchestrates a centriolar satellite-dependent response to proteotoxic stress", has now been seen by our original referees, who are experts in centrosomes (referee 1); aggresome (referee 2); and proteotoxic stress (referee 3). As you will see from their comments (attached below) they find this work of interest, but have raised some important points. Although we are also very interested in this study, we believe that their concerns should be addressed before we can consider publication in Nature Cell Biology.

Nature Cell Biology editors discuss the referee reports in detail within the editorial team, including the chief editor, to identify key referee points that should be addressed with priority, and requests that

are overruled as being beyond the scope of the current study. To guide the scope of the revisions, I have listed these points below. We are committed to providing a fair and constructive peer-review process, so please feel free to contact me if you would like to discuss any of the referee comments further.

In particular, it would be essential to:

A) Investigate the impact of PCM components depletion upon longer MG132 treatments and comment on the proposed mechanism of aggregation (Reviewer #2)

B) All other referee concerns pertaining to strengthening existing data, providing controls, methodological details, clarifications and textual changes, should also be addressed.

C) Finally please pay close attention to our guidelines on statistical and methodological reporting (listed below) as failure to do so may delay the reconsideration of the revised manuscript. In particular please provide:

- a Supplementary Figure including unprocessed images of all gels/blots in the form of a multi-page pdf file. Please ensure that blots/gels are labeled and the sections presented in the figures are clearly indicated.

- a Supplementary Table including all numerical source data in Excel format, with data for different figures provided as different sheets within a single Excel file. The file should include source data giving rise to graphical representations and statistical descriptions in the paper and for all instances where the figures present representative experiments of multiple independent repeats, the source data of all repeats should be provided.

We therefore invite you to take these points into account when revising the manuscript. In addition, when preparing the revision please:

- ensure that it conforms to our format instructions and publication policies (see below and www.nature.com/nature/authors/).

- provide a point-by-point rebuttal to the full referee reports verbatim, as provided at the end of this letter.

- provide the completed Editorial Policy Checklist (found here https://www.nature.com/authors/policies/Policy.pdf),and Reporting Summary (found here https://www.nature.com/authors/policies/ReportingSummary.pdf). This is essential for reconsideration of the manuscript and these documents will be available to editors and referees in the event of peer review. For more information see http://www.nature.com/authors/policies/availability.html or contact me.

Nature Cell Biology is committed to improving transparency in authorship. As part of our efforts in this direction, we are now requesting that all authors identified as 'corresponding author' on published papers create and link their Open Researcher and Contributor Identifier (ORCID) with their account on the Manuscript Tracking System (MTS), prior to acceptance. ORCID helps the scientific community achieve unambiguous attribution of all scholarly contributions. You can create and link your ORCID

from the home page of the MTS by clicking on ‘Modify my Springer Nature account’. For more information please visit please visit www.springernature.com/orcid.

Please submit the revised manuscript files and the point-by-point rebuttal to the referee comments using this link:

[REDACTED]

*This url links to your confidential home page and associated information about manuscripts you may have submitted or be reviewing for us. If you wish to forward this email to co-authors, please delete the link to your homepage.

We would like to receive the revision within four weeks. If submitted within this time period, reconsideration of the revised manuscript will not be affected by related studies published elsewhere, or accepted for publication in Nature Cell Biology in the meantime. We would be happy to consider a revision even after this timeframe, but in that case we will consider the published literature at the time of resubmission when assessing the file.

We hope that you will find our referees' comments, and editorial guidance helpful. Please do not hesitate to contact me if there is anything you would like to discuss.

Best wishes,

Daryl Jason David

-----

Daryl J.V. David, PhD

Senior Editor, Nature Cell Biology
Consulting Editor, Nature Communications
Nature Portfolio

Heidelberger Platz 3, 14197 Berlin, Germany
Email: daryl.david@nature.com
ORCID: https://orcid.org/0000-0002-9253-4805

Reviewers' Comments:

Reviewer #1:
Remarks to the Author:
The authors have provided a thorough response and in-depth revision. While I feel the additional data has benefited the manuscript in many ways, it has also made the overall message rather complicated. In particular, I would recommend simplifying the abstract to make it more accessible to non-experts.

Specific comments on abstract:
i. Calling centriolar satellites "a subset of centrosome proteins" is confusing.
ii. It would help the reader if the abstract contained a clear statement as to what exactly satellites do in aggresome formation instead of listing the loss of function phenotypes and rescues. Terms such as autophagy and senescence should be better introduced. iii.There is much focus on the CP110-CEP97-CEP290 module (both in abstract and title); however, there is no explanation whether this module acts at centrosome or satellites (indeed, no mention at all of these being centrosomal proteins). We learn that this module recruits aggresome components 'early' in the pathway. Could the authors clarify which pathway? Early in the aggresome assembly pathway? I wonder if seed and grow are better terms, especially if comparisons with satellites are made.

Further points:

1. The extent of autophagy increase in satellite KO differs between figures; increase in LC3-II in PCM1-KO is less obvious in Fig 4 than Supp Fig 4.

2. There appears to be a large increase pHSP27 area in PCMKO in Fig 4C when autophagy is blocked by chloroquine; I realise it is still not a complete rescue but there is more than just a ring of pHSP27 (as described on p18), which to me suggests that both aggresome seeding and expansion can occur in absence of PCM1/satellites.

3. The authors suggest that Tor inhibitor treatment of WT cells reduce aggresome sizes to same as PCM1-KO; this is an important conclusion but corresponding datasets were not tested for significance (WT-MG132-KU vs DeltaPCM1-MG132), although by eye I agree that these groups look similar.

4. Model in Fig 8: The arrow with protein translation suggests that aggresome formation driven by AZI1/CCDC14/PIBF1 occurs without protein translation. Is that so?

Reviewer #2:
Remarks to the Author:
In this manuscript, Prosser et al. provide evidence that centriolar components are required to form the perinuclear inclusion, aggresomes. By employing higher resolution microscopy, it was shown that selective centriolar and pericentriolar (PCM) proteins are expanded from typical two centriolar spots into multiple foci within the areas occupied by aggresomes (marked by pHsp27) induced by a proteasome inhibitor, MG132. Systematic knockdown of known components of centrioles and centriolar satellite showed that selective centriolar proteins, including PCM-1 as well theCP110-CEP97-CEP290 module, are required for MG132-induced aggresome and mutant Htt103Q inclusion formation. In the last two decades, significant progress has been made on the origin of protein aggregates that constitute aggresomes and the machinery involved in the dynamic concentration and clearance of aggresome at the MTOC. The identification of centriolar satellite components as a new component involved in aggresome formation adds another level of understanding to cellular proteostasis. However, there are two main issues with the current report. First, although genetic knockdown of centrosome satellite components can affect the formation or size of aggresomes, there is no clear mechanistic picture of how these factors might affect the production, transport, or processing of protein aggregates associated with aggresome biogenesis. Second, the definition of aggresomes is some analysis would need more detailed analysis. For example, the conclusion that cyclin F (CCNF)

inactivation leads to spontaneous aggresome formation requires more careful analysis. The appearance of Ub+ foci or CETN2 foci (> 4 in numbers) was interpreted as spontaneous aggresome formation upon CCNF knockdown. However, it is unclear whether this conclusion is accurate, as it is difficult to judge what the CETN2 foci are and whether they genuinely reflect a significant accumulation of protein aggregates. As aggresomes are generally thought to arise from peripheral aggregates tagged by p62, the identity of CETN2 foci needs additional protein aggregate/inclusion markers, such as p62. The definition of aggresomes is a potential issue throughout the report (See points 2 and 3 for details).
In conclusion, although the identification of centriolar machinery is in aggresome formation is novel, the lack of significant mechanistic insight and the unconventional method to define aggresomes makes it difficult to judge the key findings' importance or relevance. With these significant deficiencies, this report is not suitable for Nature Cell Biology.

Other comments:
1. The relationship between the "centriolar" foci and aggresome (protein aggregates) needs to be better defined. For example, in Fig 1, the relationship between satellite markers and pHsp70 is challenging to grasp from one representative image. Are satellite foci and pHsp27 foci always over-lapping with each other? A more quantitative co-localization analysis would be helpful.
2. The size of aggresomes in this report appears to be small compared with previous reports. It was noted that aggresomes were induced by proteasome inhibitor treatment for 5 hrs. This is a relatively short treatment to induce aggresomes for standard imaging and biochemical analysis (typical treatment time is ~ 24 hrs). Because of the concern raised in point 1, it would be reassuring to induce large aggresomes by longer inhibitor treatment and then investigate the distribution of representative PCM and satellite markers and their knockdown effect.
3. Fig 6E and F have a similar issue of defining aggresomes in senescent cells transfected with GFP-CP110. The size of the aggresome is very small in both DMSO and MG132 treated transfected cells. Therefore, it is very difficult to conclude that GFP-CP110 can promote aggresome formation in senescent cells. Because transfected and overexpressed proteins could sometimes become aggregated upon MG132 treatment, a proper GFP-CP110 mutant control should be included to guard against this potential pitfall.
4. Almost all images presented in the study only show a small cell region corresponding to the MTOC, where aggresomes typically form. This presentation might lose some vital information as protein aggregates accumulate at the cell periphery before their concentration. It would be informative to present images covering the entire cells in key figures, particularly in figures where inclusion formation was suppressed by PCM1 or CP110/CEP97/CEP290 knockdown.
5. The effects of PCM-1 and CP110/CEP97/CEP290 knockdown on mutant HTT inclusion formation is interesting (fig 6h), but proper evaluation of the expression levels of GFP-HTT in both soluble and insoluble fractions is needed. The inclusion of independent biochemical analyses on protein aggregate processing would strengthen the conclusions deduced from image analyses.
6. The effect of Hsp27 knockdown on suppressing aggresome formation has not been described previously (Supplementary fig 2). To ensure the effect of knockdown is specific, a genetic rescue experiment with Hsp27 expression plasmid is recommended.

For the revision:
In this revision, Prosser et al. have performed many experiments to address the comments. The manuscript is much improved; however, there are still unresolved issues, as discussed below:

1. In response to the concern over the small “aggresomes” induced by a short (5 hr) MG132

treatment, the authors have assessed aggresomes generated by a typical time course of 16 hrs MG132 treatment. Unlike short MG132 treatment, immunostaining shows that PCM1 signal is limited to the small center of a much larger aggresome defined by p62 or pHsp27- an interesting fact also noted by the authors (Fig 1E). Thus, PCM1 distribution within the aggresome is different from other aggresome regulators, such as HDAC6, Hsp90, BAG3, and dynein motors, which largely occupy the entire aggresome. The pattern of PCM1 is, in fact, similar to another PCM marker, γ-tubulin, which undergoes amplification in response to MG132 but remains at the center of the aggresomes (shown by several studies). Because aggresome formation requires retrograde dynein motor transport along the microtubules toward the centrosome region (MTOC), the distribution of PCM1 and γ-tubulin raises the possibility that PCM components might affect aggresome formation by interfering with the homing of protein aggregate-loaded dynein motors- an effect like that of nocodazole treatment. This conclusion is not insignificant and is consistent with the impact of PCM1 KO after prolonged MG132 treatment (Fig 4G). However, to ensure this conclusion, it would require an assessment of the impact of PCM components ablation (CP110-CEP97-CEP290 module) on aggresome formation under longer MG132 treatment conditions.
2. Fig S2 indicates that the perinuclear concentration of PCM components, like protein aggregates, is microtubule-dependent. Further, PCM1 partially co-localizes with dispersed Ub+ aggregates caused by nocodazole treatment. Because MG132 can cause the accumulation of some PCM components, could the colocalization reflect the aggregation of PCM components in response to MG132? If PCM components indeed facilitate Ub+ aggregate transport by dynein motors toward MTOC (as discussed in Point 1), what would be the mechanism?
3. Fig 5h aims to show that CCNF-depletion, which leads to the accumulation of centriole components, induces protein aggregates. However, the induction of p62 and Ub within the CP110 and PCM1 enriched area is not obvious. A line-scan might help to delineate their relationship. This portion of the study is quite difficult to understand and might not be essential for the study.

Other minor points.
1. It is known that ACY compounds do not recapitulate all phenotypes of HDAC6 genetic ablation. Unlike genetic HDAC6 knockdown, ACY738 had little effect on aggresome formation.
2. The manuscript is very long. Removal of a portion of the study (for example, the part that involves CCNF) should be considered.

Reviewer #3:
Remarks to the Author:
Revision by the authors is quite satisfactory. I recommend publishing.

READABILITY OF MANUSCRIPTS – Nature Cell Biology is read by cell biologists from diverse backgrounds, many of whom are not native English speakers. Authors should aim to communicate their findings clearly, explaining technical jargon that might be unfamiliar to non-specialists, and avoiding non-standard abbreviations. Titles and abstracts should concisely communicate the main findings of the study, and the background, rationale, results and conclusions should be clearly explained in the manuscript in a manner accessible to a broad cell biology audience. Nature Cell Biology uses British spelling.

ARTICLE FORMAT

TITLE – should be no more than 100 characters including spaces, without punctuation and avoiding technical terms, abbreviations, and active verbs..

AUTHOR NAMES – should be given in full.

AUTHOR AFFILIATIONS – should be denoted with numerical superscripts (not symbols) preceding the names. Full addresses should be included, with US states in full and providing zip/post codes. The corresponding author is denoted by: "Correspondence should be addressed to [initials]."

ABSTRACT – should not exceed 150 words and should be unreferenced. This paragraph is the most visible part of the paper and should briefly outline the background and rationale for the work, and accurately summarize the main results and conclusions. Key genes, proteins and organisms should be specified to ensure discoverability of the paper in online searches.

TEXT – the main text consists of the Introduction, Results, and Discussion sections and must not exceed 3500 words including the abstract. The Introduction should expand on the background relating to the work. The Results should be divided in subsections with subheadings, and should provide a concise and accurate description of the experimental findings. The Discussion should expand on the findings and their implications. All relevant primary literature should be cited, in particular when discussing the background and specific findings.

ACKNOWLEDGEMENTS – should be kept brief. Professional titles and affiliations are unnecessary. Grant numbers can be listed.

AUTHOR CONTRIBUTIONS – must be included after the Acknowledgements, detailing the contributions of each author to the paper (e.g. experimental work, project planning, data analysis etc.). Each author should be listed by his/her initials.

FINANCIAL AND NON-FINANCIAL COMPETING INTERESTS – the authors must include one of three declarations: (1) that they have no financial and non-financial competing interests; (2) that they have financial and non-financial competing interests; or (3) that they decline to respond, after the Author Contributions section. This statement will be published with the article, and in cases where financial and non-financial competing interests are declared, these will be itemized in a web supplement to the article. For further details please see https://www.nature.com/licenceforms/nrg/competing-interests.pdf.

REFERENCES – are limited to a total of 70 in the main text and Methods combined,. They must be

numbered sequentially as they appear in the main text, tables and figure legends and Methods and must follow the precise style of Nature Cell Biology references. References only cited in the Methods should be numbered consecutively following the last reference cited in the main text. References only associated with Supplementary Information (e.g. in supplementary legends) do not count toward the total reference limit and do not need to be cited in numerical continuity with references in the main text. Only published papers can be cited, and each publication cited should be included in the numbered reference list, which should include the manuscript titles. Footnotes are not permitted.

METHODS – Nature Cell Biology publishes methods online. The methods section should be provided as a separate Word document, which will be copyedited and appended to the manuscript PDF, and incorporated within the HTML format of the paper.

Methods should be written concisely, but should contain all elements necessary to allow interpretation and replication of the results. As a guideline, Methods sections typically do not exceed 3,000 words. The Methods should be divided into subsections listing reagents and techniques. When citing previous methods, accurate references should be provided and any alterations should be noted. Information must be provided about: antibody dilutions, company names, catalogue numbers and clone numbers for monoclonal antibodies; sequences of RNAi and cDNA probes/primers or company names and catalogue numbers if reagents are commercial; cell line names, sources and information on cell line identity and authentication. Animal studies and experiments involving human subjects must be reported in detail, identifying the committees approving the protocols. For studies involving human subjects/samples, a statement must be included confirming that informed consent was obtained. Statistical analyses and information on the reproducibility of experimental results should be provided in a section titled "Statistics and Reproducibility".

All Nature Cell Biology manuscripts submitted on or after March 21 2016, must include a Data availability statement as a separate section after Methods but before references, under the heading "Data Availability". For Springer Nature policies on data availability see http://www.nature.com/authors/policies/availability.html; for more information on this particular policy see http://www.nature.com/authors/policies/data/data-availability-statements-data-citations.pdf. The Data availability statement should include:

• Accession codes for primary datasets (generated during the study under consideration and designated as "primary accessions") and secondary datasets (published datasets reanalysed during the study under consideration, designated as "referenced accessions"). For primary accessions data should be made public to coincide with publication of the manuscript. A list of data types for which submission to community-endorsed public repositories is mandated (including sequence, structure, microarray, deep sequencing data) can be found here http://www.nature.com/authors/policies/availability.html#data.

• Unique identifiers (accession codes, DOIs or other unique persistent identifier) and hyperlinks for datasets deposited in an approved repository, but for which data deposition is not mandated (see here for details http://www.nature.com/sdata/data-policies/repositories).

• At a minimum, please include a statement confirming that all relevant data are available from the authors, and/or are included with the manuscript (e.g. as source data or supplementary information), listing which data are included (e.g. by figure panels and data types) and mentioning any restrictions on availability.

• If a dataset has a Digital Object Identifier (DOI) as its unique identifier, we strongly encourage including this in the Reference list and citing the dataset in the Methods.

We recommend that you upload the step-by-step protocols used in this manuscript to the Protocol Exchange. More details can found at www.nature.com/protocolexchange/about.

DISPLAY ITEMS – main display items are limited to 6-8 main figures and/or main tables. For Supplementary Information see below.

FIGURES – Colour figure publication costs $395 per colour figure. All panels of a multi-panel figure must be logically connected and arranged as they would appear in the final version. Unnecessary figures and figure panels should be avoided (e.g. data presented in small tables could be stated briefly in the text instead).

All imaging data should be accompanied by scale bars, which should be defined in the legend. Cropped images of gels/blots are acceptable, but need to be accompanied by size markers, and to retain visible background signal within the linear range (i.e. should not be saturated). The boundaries of panels with low background have to be demarked with black lines. Splicing of panels should only be considered if unavoidable, and must be clearly marked on the figure, and noted in the legend with a statement on whether the samples were obtained and processed simultaneously. Quantitative comparisons between samples on different gels/blots are discouraged; if this is unavoidable, it has be performed for samples derived from the same experiment with gels/blots were processed in parallel, which needs to be stated in the legend.

Figures should be provided at approximately the size that they are to be printed at (single column is 86 mm, double column is 170 mm) and should not exceed an A4 page (8.5 x 11"). Reduction to the scale that will be used on the page is not necessary, but multi-panel figures should be sized so that the whole figure can be reduced by the same amount at the smallest size at which essential details in each panel are visible. In the interest of our colour-blind readers we ask that you avoid using red and green for contrast in figures. Replacing red with magenta and green with turquoise are two possible colour-safe alternatives. Lines with widths of less than 1 point should be avoided. Sans serif typefaces, such as Helvetica (preferred) or Arial should be used. All text that forms part of a figure should be rewritable and removable.

We accept files from the following graphics packages in either PC or Macintosh format:

- For line art, graphs, charts and schematics we prefer Adobe Illustrator (.AI), Encapsulated PostScript (.EPS) or Portable Document Format (.PDF). Files should be saved or exported as such directly from the application in which they were made, to allow us to restyle them according to our journal house style.

- We accept PowerPoint (.PPT) files if they are fully editable. However, please refrain from adding PowerPoint graphical effects to objects, as this results in them outputting poor quality raster art. Text used for PowerPoint figures should be Helvetica (preferred) or Arial.

- We do not recommend using Adobe Photoshop for designing figures, but we can accept Photoshop

generated (.PSD or .TIFF) files only if each element included in the figure (text, labels, pictures, graphs, arrows and scale bars) are on separate layers. All text should be editable in ‘type layers’ and line-art such as graphs and other simple schematics should be preserved and embedded within 'vector smart objects’ - not flattened raster/bitmap graphics.

- Some programs can generate Postscript by 'printing to file' (found in the Print dialogue). If using an application not listed above, save the file in PostScript format or email our Art Editor, Allen Beattie for advice (a.beattie@nature.com).

Regardless of format, all figures must be vector graphic compatible files, not supplied in a flattened raster/bitmap graphics format, but should be fully editable, allowing us to highlight/copy/paste all text and move individual parts of the figures (i.e. arrows, lines, x and y axes, graphs, tick marks, scale bars etc). The only parts of the figure that should be in pixel raster/bitmap format are photographic images or 3D rendered graphics/complex technical illustrations.

All placed images (i.e. a photo incorporated into a figure) should be on a separate layer and independent from any superimposed scale bars or text. Individual photographic images must be a minimum of 300+ DPI (at actual size) or kept constant from the original picture acquisition and not decreased in resolution post image acquisition. All colour artwork should be RGB format.

FIGURE LEGENDS – must not exceed 350 words for each figure to allow fit on a single printed NCB page together with the figure. They must include a brief title for the whole figure, and short descriptions of each panel with definitions of the symbols used, but without detailing methodology.

TABLES – main tables should be provided as individual Word files, together with a brief title and legend. For supplementary tables see below.

SUPPLEMENTARY INFORMATION – Supplementary information is material directly relevant to the conclusion of a paper, but which cannot be included in the printed version in order to keep the manuscript concise and accessible to the general reader. Supplementary information is an integral part of a Nature Cell Biology publication and should be prepared and presented with as much care as the main display item, but it must not include non-essential data or text, which may be removed at the editor's discretion. All supplementary material is fully peer-reviewed and published online as part of the HTML version of the manuscript. Supplementary Figures and Supplementary Notes are appended at the end of the main PDF of the published manuscript.

Supplementary items should relate to a main text figure, wherever possible, and should be mentioned sequentially in the main manuscript, designated as Supplementary Figure, Table, Video, or Note, and numbered continuously (e.g. Supplementary Figure 1, Supplementary Figure 2, Supplementary Table 1, Supplementary Table 2 etc.).

Unprocessed scans of all key data generated through electrophoretic separation techniques need to be presented in a supplementary figure that should be labeled and numbered as the final supplementary figure, and should be mentioned in every relevant figure legend. This figure does not count towards the total number of figures and is the only figure that can be displayed over multiple pages, but should be provided as a single file, in PDF or TIFF format. Data in this figure can be displayed in a

relatively informal style, but size markers and the figures panels corresponding to the presented data must be indicated.

The total number of Supplementary Figures (not including the "unprocessed scans" Supplementary Figure) should not exceed the number of main display items (figures and/or tables (see our Guide to Authors and March 2012 editorial http://www.nature.com/ncb/authors/submit/index.html#suppinfo; http://www.nature.com/ncb/journal/v14/n3/index.html#ed). No restrictions apply to Supplementary Tables or Videos, but we advise authors to be selective in including supplemental data.

Each Supplementary Figure should be provided as a single page and as an individual file in one of our accepted figure formats and should be presented according to our figure guidelines (see above). Supplementary Tables should be provided as individual Excel files. Supplementary Videos should be provided as .avi or .mov files up to 50 MB in size. Supplementary Figures, Tables and Videos much be accompanied by a separate Word document including titles and legends.

GUIDELINES FOR EXPERIMENTAL AND STATISTICAL REPORTING

REPORTING REQUIREMENTS – To improve the quality of methods and statistics reporting in our papers we have recently revised the reporting checklist we introduced in 2013. We are now asking all life sciences authors to complete two items: an Editorial Policy Checklist (found here https://www.nature.com/authors/policies/Policy.pdf) that verifies compliance with all required editorial policies and a Reporting Summary (found here https://www.nature.com/authors/policies/ReportingSummary.pdf) that collects information on experimental design and reagents. These documents are available to referees to aid the evaluation of the manuscript. Please note that these forms are dynamic 'smart pdfs' and must therefore be downloaded and completed in Adobe Reader. We will then flatten them for ease of use by the reviewers. If you would like to reference the guidance text as you complete the template, please access these flattened versions at http://www.nature.com/authors/policies/availability.html.

STATISTICS – Wherever statistics have been derived the legend needs to provide the n number (i.e. the sample size used to derive statistics) as a precise value (not a range), and define what this value represents. Error bars need to be defined in the legends (e.g. SD, SEM) together with a measure of centre (e.g. mean, median). Box plots need to be defined in terms of minima, maxima, centre, and percentiles. Ranges are more appropriate than standard errors for small data sets. Wherever statistical significance has been derived, precise p values need to be provided and the statistical test used needs to be stated in the legend. Statistics such as error bars must not be derived from n<3. For sample sizes of n<5 please plot the individual data points rather than providing bar graphs. Deriving statistics from technical replicate samples, rather than biological replicates is strongly discouraged. Wherever statistical significance has been derived, precise p values need to be provided and the statistical test stated in the legend.

Information on how many times each experiment was repeated independently with similar results needs to be provided in the legends and/or Methods for all experiments, and in particular wherever representative experiments are shown.

We strongly recommend the presentation of source data for graphical and statistical analyses as a separate Supplementary Table, and request that source data for all independent repeats are provided

when representative experiments of multiple independent repeats, or averages of two independent experiments are presented. This supplementary table should be in Excel format, with data for different figures provided as different sheets within a single Excel file. It should be labelled and numbered as one of the supplementary tables, titled “Statistics Source Data”, and mentioned in all relevant figure legends.

**Author Rebuttal, first revision:**

We would like to thank the Reviewers for their judicious comments and thoughtful suggestions. Below is our detailed point-by point response (our responses are in bold and the original comments in their entirety are in italics). The referee reports have been very helpful, and we hope that the Reviewers will now find the revised version of our manuscript suitable for publication in Nature Cell Biology.

Reviewers' Comments:
Reviewer #1:
Remarks to the Author:
The authors have provided a thorough response and in-depth revision. While I feel the additional data has benefited the manuscript in many ways, it has also made the overall message rather complicated. In particular, I would recommend simplifying the abstract to make it more accessible to non-experts.

Specific comments on abstract:
i. Calling centriolar satellites "a subset of centrosome proteins" is confusing.
ii. It would help the reader if the abstract contained a clear statement as to what exactly satellites do in aggresome formation instead of listing the loss of function phenotypes and rescues. Terms such as autophagy and senescence should be better introduced. iii.There is much focus on the CP110-CEP97-CEP290 module (both in abstract and title); however, there is no explanation whether this module acts at centrosome or satellites (indeed, no mention at all of these being centrosomal proteins). We learn that this module recruits aggresome components 'early' in the pathway. Could the authors clarify which pathway? Early in the aggresome assembly pathway? I wonder if seed and grow are better terms, especially if comparisons with satellites are made.

We appreciate the Reviewer raising these concerns over the clarity of the abstract. We have rewritten the abstract with these points in mind to simplify it and improve its accessibility. We have also modified the title.

Further points:

1. The extent of autophagy increase in satellite KO differs between figures; increase in LC3-II in PCM1-KO is less obvious in Fig 4 than Supp Fig 4.

We thank the Reviewer for pointing this out. We have now replaced the immunoblot in Fig. 4a for one that more clearly shows the difference in LC3-II levels in WT and PCM1 KO cells during MG132. We also now provide the fold change in LC3-II levels for this blot.

2. There appears to be a large increase pHSP27 area in PCMKO in Fig 4C when autophagy is blocked by chloroquine; I realise it is still not a complete rescue but there is more than just a ring of pHSP27 (as described on p18), which to me suggests that both aggresome seeding and expansion can occur in absence of PCM1/satellites.
We thank the Reviewer for bringing this to our attention. There was indeed a moderate degree of rescue in pHSP27 in the PCM1 KO when autophagy was inhibited with chloroquine. This does suggest that a degree of aggresome expansion can occur in the absence of satellites, but that the extent of this is dependent upon the status of autophagic activity. We have added this point to the discussion on Page 18.

3. The authors suggest that Tor inhibitor treatment of WT cells reduce aggresome sizes to same as PCM1-KO; this is an important conclusion but corresponding datasets were not tested for significance (WT-MG132-KU vs DeltaPCM1-MG132), although by eye I agree that these groups look similar.
We apologize for not including this comparison in the previous revision. The median aggresome size in the WT MG132 KU sample was 28 pixels2, while in the PCM1 KO MG132 it was 54 pixels2, with a p value of 1.64 x10-5. This means that the inhibition of autophagy reduced aggresome size in WT cells below that in the PCM1 KO, consequently we have amended this point in the text on Page 10. The difference is likely due to the experimental setup, with KU being added to the cells two hours before the addition of MG132 resulting in pre-activation of autophagy before proteasomal inhibition. In PCM1 KO cells that were treated comparably to WT cells with KU (the PCM1 KO MG132 KU sample), the median aggresome size was 28.5, which is not significantly different from the size in WT cells. The p values are available in the Source Data file and have been indicated on the plot in Fig. 4b.

4. Model in Fig 8: The arrow with protein translation suggests that aggresome formation driven by AZI1/CCDC14/PIBF1 occurs without protein translation. Is that so?
We thank the Reviewer for highlighting this issue. We have now modified the model (new Fig. 8a) to make it clear that no aggresome formation occurs in the absence of active protein translation.

Reviewer #2: For the revision:
In this revision, Prosser et al. have performed many experiments to address the comments. The manuscript is much improved; however, there are still unresolved issues, as discussed below:

1. In response to the concern over the small “aggresomes” induced by a short (5 hr) MG132 treatment, the authors have assessed aggresomes generated by a typical time course of 16 hrs MG132 treatment. Unlike short MG132 treatment, immunostaining shows that PCM1 signal is limited to the small center of

a much larger aggresome defined by p62 or pHsp27- an interesting fact also noted by the authors (Fig 1E). Thus, PCM1 distribution within the aggresome is different from other aggresome regulators, such as HDAC6, Hsp90, BAG3, and dynein motors, which largely occupy the entire aggresome. The pattern of PCM1 is, in fact, similar to another PCM marker, ⍰- tubulin, which undergoes amplification in response to MG132 but remains at the center of the aggresomes (shown by several studies). Because aggresome formation requires retrograde dynein motor transport along the microtubules toward the centrosome region (MTOC), the distribution of PCM1 and ⍰-tubulin raises the possibility that PCM components might affect aggresome formation by interfering with the homing of protein aggregate-loaded dynein motors- an effect like that of nocodazole treatment. This conclusion is not insignificant and is consistent with the impact of PCM1 KO after prolonged MG132 treatment (Fig 4G).
We agree with the Reviewer that satellites might affect aggresome formation by interfering with the homing of protein aggregates to the centrosome and have incorporated this point into our revised discussion on Page 17.

However, to ensure this conclusion, it would require an assessment of the impact of PCM components ablation (CP110-CEP97-CEP290 module) on aggresome formation under longer MG132 treatment conditions.

The assessment of aggresome formation during longer MG132 treatment in cells depleted on the CP110-CEP97-CEP290 module components is an important experiment to include. In the previous revision, data for CP110 depletion under these conditions was presented in Fig. 6j,k. We had performed and quantified CEP97 and CEP290 depletion in the same experiments but had excluded the results from the figure for space considerations. In this current version, we now present the assessment of aggresome formation in cells depleted of CP110, CEP97 and CEP290 depletion during longer MG132 treatment conditions (new Fig. 5k). These results support the conclusion that the homing of protein aggregates to the centrosome is impeded in the absence of CP110, CEP97 and CEP290.

2. Fig S2 indicates that the perinuclear concentration of PCM components, like protein aggregates, is microtubule-dependent. Further, PCM1 partially co-localizes with dispersed Ub+ aggregates caused by nocodazole treatment. Because MG132 can cause the accumulation of some PCM components, could the colocalization reflect the aggregation of PCM components in response to MG132?
We agree that the colocalization of PCM1 with Ub+ aggregates in cells treated with nocodazole could reflect the aggregation of PCM1 in response to MG132. However, co- localization between PCM1 and Ub+ was not observed in cells treated with the HDAC6 inhibitor Acy-1215(Supplementary Fig. 2k), suggesting that the interaction of PCM1 with Ub+ aggregates depends on HDAC6 activity. This is mentioned in the text on Page 8.

If PCM components indeed facilitate Ub+ aggregate transport by dynein motors toward MTOC (as discussed in Point 1), what would be the mechanism?

Like aggresome assembly, the movement of centriolar satellites to the centrosome depends on retrograde dynein-dependent transport along microtubules. A key function of satellites is the recruitment of proteins to the centrosome via the dynein-dynactin motor system and several satellite proteins have been shown to interact directly with dynein-dynactin. We thus postulate that satellites facilitate the trafficking of protein aggregates to the centrosome via dynein-mediated transport. Whether they act as adaptors between aggregates and dynein-dynactin or function with HDAC6 in aggregate recruitment will be the focus of future studies. We have added these points to our revised discussion on Page 17.

3. Fig 5h aims to show that CCNF-depletion, which leads to the accumulation of centriole components, induces protein aggregates. However, the induction of p62 and Ub within the CP110 and PCM1 enriched area is not obvious. A line-scan might help to delineate their relationship. This portion of the study is quite difficult to understand and might not be essential for the study.
We agree with the Reviewer that, while interesting, the CCNF-depletion portion detracts from the overall study. In line with minor point 2 below, we have removed the data and text relating to CCNF-depletion from the revised manuscript. However, we have moved the data that pertained to centriole number within the aggresome from former Fig. 5a-c to Supplementary Fig. 1d-f as we felt it was important to include clarification that the additional foci of CETN2, CP110 and CEP97 we observed within the aggresome did not represent centriole amplification.

Other minor points.

1. It is known that ACY compounds do not recapitulate all phenotypes of HDAC6 genetic ablation. Unlike genetic HDAC6 knockdown, ACY738 had little effect on aggresome formation.
We thank the Reviewer for highlighting this point which we have now added to the text on Page 7.

2. The manuscript is very long. Removal of a portion of the study (for example, the part that involves CCNF) should be considered.
We agree with the Reviewer and have removed the portion of the study involving CCNF- depletion as suggested.

Reviewer #3:
Remarks to the Author:
Revision by the authors is quite satisfactory. I recommend publishing.
We thank the Reviewer for their positive comments.

**Decision Letter, second revision:**

13th December 2021

Dear Dr. Pelletier,

Thank you for submitting your revised manuscript "Aggresome assembly at the centrosome is driven by CP110-CEP97-CEP290 and centriolar satellites" (NCB-P44740B). It has now been seen by the original referees and their comments are below. The reviewers find that the paper has improved in revision, and therefore we'll be happy in principle to publish it in Nature Cell Biology, pending minor revisions to satisfy the referees' final requests and to comply with our editorial and formatting guidelines.

**As the current version of your manuscript is in a PDF format, please email us a copy of the file in an editable format (Microsoft Word or LaTex)-- we can not proceed with PDFs at this stage.**

We are now performing detailed checks on your paper and will send you a checklist detailing our editorial and formatting requirements in about a week. Please do not upload the final materials and make any revisions until you receive this additional information from us.

Thank you again for your interest in Nature Cell Biology Please do not hesitate to contact me if you have any questions.

Sincerely,

-----

Daryl J.V. David, PhD

Senior Editor, Nature Cell Biology
Consulting Editor, Nature Communications
Nature Portfolio

Heidelberger Platz 3, 14197 Berlin, Germany
Email: daryl.david@nature.com
ORCID: https://orcid.org/0000-0002-9253-4805

Reviewer #1 (Remarks to the Author):

The authors have improved the clarity of the abstract and I recommend the paper for publication.

Reviewer #2 (Remarks to the Author):

The authors have provided reasonable anwers to key issues.

-----------

23rd December 2021

Dear Dr. Pelletier,

Thank you for your patience as we've prepared the guidelines for final submission of your Nature Cell Biology manuscript, "Aggresome assembly at the centrosome is driven by CP110-CEP97-CEP290 and centriolar satellites" (NCB-P44740B). Please carefully follow the step-by-step instructions provided in the attached file, and add a response in each row of the table to indicate the changes that you have made. Please also check and comment on any additional marked-up edits we have proposed within the text. Ensuring that each point is addressed will help to ensure that your revised manuscript can be swiftly handed over to our production team.

We would like to start working on your revised paper, with all of the requested files and forms, as soon as possible (preferably within one week). Please get in contact with us if you anticipate delays.

When you upload your final materials, please include a point-by-point response to any remaining reviewer comments.

If you have not done so already, please alert us to any related manuscripts from your group that are under consideration or in press at other journals, or are being written up for submission to other journals (see: https://www.nature.com/nature-research/editorial-policies/plagiarism#policy-on-duplicate-publication for details).

In recognition of the time and expertise our reviewers provide to Nature Cell Biology's editorial process, we would like to formally acknowledge their contribution to the external peer review of your manuscript entitled "Aggresome assembly at the centrosome is driven by CP110-CEP97-CEP290 and centriolar satellites". For those reviewers who give their assent, we will be publishing their names alongside the published article.

Nature Cell Biology offers a Transparent Peer Review option for new original research manuscripts submitted after December 1st, 2019. As part of this initiative, we encourage our authors to support increased transparency into the peer review process by agreeing to have the reviewer comments, author rebuttal letters, and editorial decision letters published as a Supplementary item. When you submit your final files please clearly state in your cover letter whether or not you would like to participate in this initiative. Please note that failure to state your preference will result in delays in accepting your manuscript for publication.

**Cover suggestions**

As you prepare your final files we encourage you to consider whether you have any images or illustrations that may be appropriate for use on the cover of Nature Cell Biology.

Covers should be both aesthetically appealing and scientifically relevant, and should be supplied at the best quality available. Due to the prominence of these images, we do not generally select images featuring faces, children, text, graphs, schematic drawings, or collages on our covers.

We accept TIFF, JPEG, PNG or PSD file formats (a layered PSD file would be ideal), and the image should be at least 300ppi resolution (preferably 600-1200 ppi), in CMYK colour mode.

If your image is selected, we may also use it on the journal website as a banner image, and may need to make artistic alterations to fit our journal style.

Please submit your suggestions, clearly labeled, along with your final files. We'll be in touch if more information is needed.

Nature Cell Biology has now transitioned to a unified Rights Collection system which will allow our Author Services team to quickly and easily collect the rights and permissions required to publish your work. Approximately 10 days after your paper is formally accepted, you will receive an email in providing you with a link to complete the grant of rights. If your paper is eligible for Open Access, our Author Services team will also be in touch regarding any additional information that may be required to arrange payment for your article.

Please note that *Nature Cell Biology* is a Transformative Journal (TJ). Authors may publish their research with us through the traditional subscription access route or make their paper immediately open access through payment of an article-processing charge (APC). Authors will not be required to make a final decision about access to their article until it has been accepted. Find out more about Transformative Journals

**Authors may need to take specific actions to achieve compliance with funder and institutional open access mandates.** For submissions from January 2021, if your research is supported by a funder that requires immediate open access (e.g. according to Plan S principles) then you should select the gold OA route, and we will direct you to the compliant route where possible. For authors selecting the subscription publication route our standard licensing terms will need to be accepted, including our self-archiving policies. Those standard licensing terms will supersede any other terms that the author or any third party may assert apply to any version of the manuscript.

Please note that you will not receive your proofs until the publishing agreement has been received through our system.

For information regarding our different publishing models please see our Transformative Journals page. If you have any questions about costs, Open Access requirements, or our legal forms, please contact ASJournals@springernature.com.

Please use the following link for uploading these materials:
[REDACTED]

If you have any further questions, please feel free to contact me.

Best regards,

Nyx Hills
Staff

Nature Cell Biology

On behalf of

-----

Daryl J.V. David, PhD

Senior Editor, Nature Cell Biology
Consulting Editor, Nature Communications
Nature Portfolio

Heidelberger Platz 3, 14197 Berlin, Germany
Email: daryl.david@nature.com
ORCID: https://orcid.org/0000-0002-9253-4805

Reviewer #1:
Remarks to the Author:
The authors have improved the clarity of the abstract and I recommend the paper for publication.

Reviewer #2:
Remarks to the Author:
The authors have provided reasonable anwers to key issues.

**Final Decision Letter:**

Dear Dr Pelletier,

I am pleased to inform you that your manuscript, "Aggresome assembly at the centrosome is driven by CP110-CEP97-CEP290 and centriolar satellites", has now been accepted for publication in Nature Cell Biology.

Thank you for sending us the final manuscript files to be processed for print and online production, and for returning the manuscript checklists and other forms. Your manuscript will now be passed to our production team who will be in contact with you if there are any questions with the production quality of supplied figures and text.

Over the next few weeks, your paper will be copyedited to ensure that it conforms to Nature Cell Biology style. Once your paper is typeset, you will receive an email with a link to choose the appropriate publishing options for your paper and our Author Services team will be in touch regarding any additional information that may be required.

After the grant of rights is completed, you will receive a link to your electronic proof via email with a

request to make any corrections within 48 hours. If, when you receive your proof, you cannot meet this deadline, please inform us at rjsproduction@springernature.com immediately.

You will not receive your proofs until the publishing agreement has been received through our system.

Due to the importance of these deadlines, we ask that you please let us know now whether you will be difficult to contact over the next month. If this is the case, we ask you provide us with the contact information (email, phone and fax) of someone who will be able to check the proofs on your behalf, and who will be available to address any last-minute problems.

If you have any questions about our publishing options, costs, Open Access requirements, or our legal forms, please contact ASJournals@springernature.com

Once your paper has been scheduled for online publication, the Nature press office will be in touch to confirm the details. An online order form for reprints of your paper is available at https://www.nature.com/reprints/author-reprints.html. All co-authors, authors' institutions and authors' funding agencies can order reprints using the form appropriate to their geographical region.

Publication is conditional on the manuscript not being published elsewhere and on there being no announcement of this work to any media outlet until the online publication date in Nature Cell Biology.

Please note that *Nature Cell Biology* is a Transformative Journal (TJ). Authors may publish their research with us through the traditional subscription access route or make their paper immediately open access through payment of an article-processing charge (APC). Authors will not be required to make a final decision about access to their article until it has been accepted. Find out more about Transformative Journals

**Authors may need to take specific actions to achieve compliance with funder and institutional open access mandates.** For submissions from January 2021, if your research is supported by a funder that requires immediate open access (e.g. according to Plan S principles) then you should select the gold OA route, and we will direct you to the compliant route where possible. For authors selecting the subscription publication route our standard licensing terms will need to be accepted, including our self-archiving policies. Those standard licensing terms will supersede any other terms that the author or any third party may assert apply to any version of the manuscript.

To assist our authors in disseminating their research to the broader community, our SharedIt initiative provides you with a unique shareable link that will allow anyone (with or without a subscription) to read the published article. Recipients of the link with a subscription will also be able to download and print the PDF.

If your paper includes color figures, please be aware that in order to help cover some of the additional cost of four-color reproduction, Nature Research charges our authors a fee for the printing of their color figures. Please contact our offices for exact pricing and details.

As soon as your article is published, you will receive an automated email with your shareable link.

If you have not already done so, we strongly recommend that you upload the step-by-step protocols used in this manuscript to the Protocol Exchange (www.nature.com/protocolexchange), an open online

resource established by Nature Protocols that allows researchers to share their detailed experimental know-how. All uploaded protocols are made freely available, assigned DOIs for ease of citation and are fully searchable through nature.com. Protocols and the Nature and Nature research journal papers in which they are used can be linked to one another, and this link is clearly and prominently visible in the online versions of both papers. Authors who performed the specific experiments can act as primary authors for the Protocol as they will be best placed to share the methodology details, but the Corresponding Author of the present research paper should be included as one of the authors. By uploading your Protocols to Protocol Exchange, you are enabling researchers to more readily reproduce or adapt the methodology you use, as well as increasing the visibility of your protocols and papers. You can also establish a dedicated page to collect your lab Protocols. Further information can be found at www.nature.com/protocolexchange/about

You can use a single sign-on for all your accounts, view the status of all your manuscript submissions and reviews, access usage statistics for your published articles and download a record of your refereeing activity for the Nature journals.

Please feel free to contact us if you have any questions.

With kind regards,
Daryl

-----
Daryl J.V. David, PhD

Senior Editor, Nature Cell Biology
Consulting Editor, Nature Communications
Nature Portfolio

Heidelberger Platz 3, 14197 Berlin, Germany
Email: daryl.david@nature.com
ORCID: https://orcid.org/0000-0002-9253-4805

Click here if you would like to recommend Nature Cell Biology to your librarian
http://www.nature.com/subscriptions/recommend.html#forms
